# One-pot Diels–Alder cycloaddition/gold(I)-catalyzed 6-*endo-dig* cyclization for the synthesis of the complex bicyclo[3.3.1]alkenone framework

**DOI:** 10.3762/bjoc.7.114

**Published:** 2011-07-22

**Authors:** Boubacar Sow, Gabriel Bellavance, Francis Barabé, Louis Barriault

**Affiliations:** 1 Department of Chemistry, 10 Marie Curie, University of Ottawa, Ottawa, Canada, K1N 6N5

**Keywords:** bicyclo[3.3.1]nonenone, carbocyclization, Diels–Alder, gold catalysis, one-pot process

## Abstract

The rapid synthesis of bicyclo[*m*.*n*.1]alkanone cores possessing quaternary carbon centers adjacent to a bridged ketone represents a significant synthetic challenge. This type of architectural feature is embedded in various complex biologically active compounds such as hyperforin and garsubellin A. Herein, we report a highly diastereoselective one-pot Diels–Alder reaction/Au(I)-catalyzed carbocyclization to generate bicyclo[3.3.1]alkanones in yields ranging from 48–93%.

## Introduction

**Figure 1:**
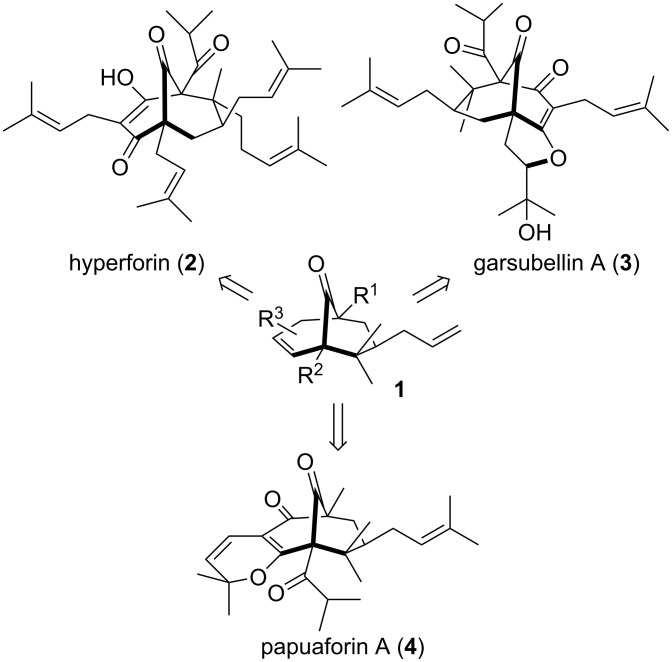
Structures of naturally occurring PPAPs.

Highly oxygenated and densely substituted carbon-bridged medium sized rings such as **1** are commonly found in nature as structural frameworks of many important bioactive natural products, and in particular, polycyclic polyprenylated acetylphloroglucinols (PPAPs) (Figure 1) [1]. In the past decades, more than 100 PPAPs exhibiting a wide variety of biological activities (antibiotic, anti-HIV, anti-oxidant, etc.) have been isolated from *Guttiferrea* plants such as hyperforin (**2**) [2–6] and garsubellin A (**3**) [7–8]. The challenging synthesis of PPAP structures combined with their promising therapeutic potential has drawn attention from several research groups [9–12].

**Scheme 1:**
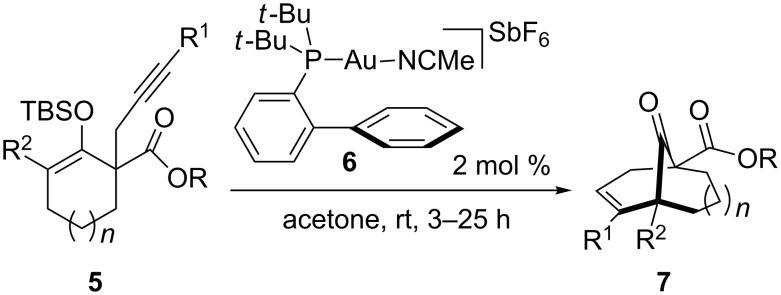
Gold(I)-catalyzed 6-*endo-dig* cyclization.

In 2009, we reported a mild and highly efficient method to generate carbon-bridged frameworks of various sizes through a gold(I)-catalyzed carbocyclization [13]. Although the cyclization of enol ether **5** can produce 5-*exo* and 6-*endo* products, we found that gold complexes **6**, having bulky phosphine ligands such as 2-bis(*tert*-butylphosphino)biphenyl, gave exclusively the 6-*endo-dig* cyclized products **7** (Scheme 1). In the course of our studies directed towards the synthesis of naturally occurring PPAPs and related carbon-bridged ketone scaffolds, we envisioned that PPAP framework **1** could be generated via a Au(I)-catalyzed cyclization [14–22].

## Results and Discussion

**Scheme 2:**
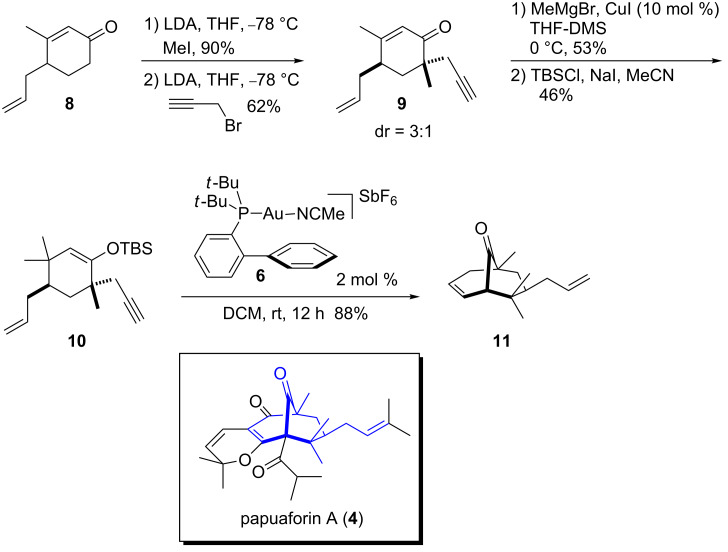
Synthesis of papuaforin A core **4**.

The synthesis began by a *C*-alkylation of enone **8** [23] using LDA and MeI to give the corresponding ketone in 90% yield (Scheme 2). A second alkylation to add the propargyl chain was carried out using LDA and propargyl bromide to afford **9** in 62% yield as an inseparable mixture of diastereomers (dr = 3:1). Subsequently, conjugate addition of methylmagnesium bromide in the presence of a catalytic amount of CuI provided the corresponding ketone in 53% yield. The ketone was then treated with TBSCl, NaI and triethylamine to give the desired silylenol ether **10** in 46% yield, which upon exposure to the Au(I) complex **6** (2 mol %) provided the desired bicyclo[3.3.1]nonenone **11** in 88% yield. It is important to note that the Au(I)-catalyzed cyclization proceeds in high yields in a sterically congested environment. The synthesis of the core of papuaforin (**11**) was achieved in five steps from enone **8**.

**Scheme 3:**
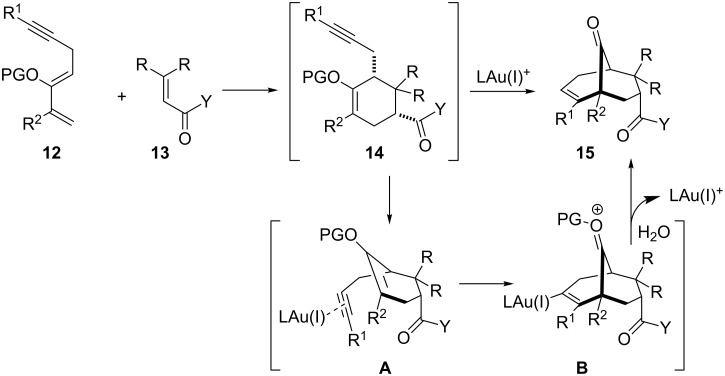
Proposed domino Diels–Alder reaction/gold(I)-catalyzed cyclization.

However, one might recognize that the low chemical yields encountered in some steps undermine the efficacy of the Au(I)-catalyzed cyclization approach. In order to solve this issue, we assumed that bicyclo[3.3.1]nonenone scaffolds can be directly obtained through an intermolecular Diels–Alder reaction/Au(I)-catalyzed 6-*endo-dig* carbocyclization (Scheme 3). Cycloaddition between diene **12** and dienophile **13** should provide the endo cycloadduct **14**, which, in the presence of a gold(I) catalyst, would form the gold complex **A**. This undergoes a carbocyclization of enol ether [24–31] to afford intermediate **B**, which after proto-deauration and hydrolysis affords the bridgehead ketone **15**. The attractive feature of this process resides in the ability to generate four new stereogenic centers and three new C–C bonds in one single operation.

**Scheme 4:**
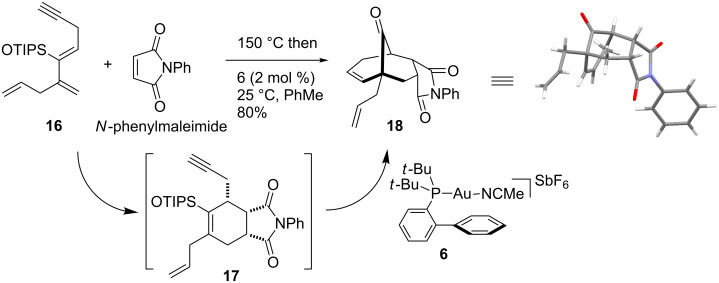
One-pot Diels–Alder cycloaddition/gold(I) catalyzed carbocyclization.

To validate the above hypothesis, diene **16** (*Z*-isomer) was heated with *N*-phenylmaleimide in toluene at 150 °C, for two hours, by microwave irradiation (Scheme 4) (see Supporting Information File 1 for experimental procedures). The solution containing the Diels–Alder adduct **17** was cooled down to room temperature and 2 mol % of Au(I) complex **6** was added. The bridgedhead ketone **18** was obtained in 80% yield as a single diastereomer. The relative stereochemistry of **18** was unambiguously established by X-ray analysis (see Supporting Information File 2). With this result in hand, we explored the scope of this sequential reaction (Table 1).

**Table 1:** Results of the one-pot Diels–Alder reaction/Au(I)-catalyzed cyclization.

entry	diene	dienophile	product (yield)^a^

1	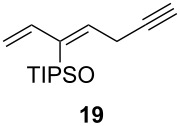	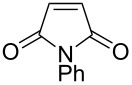	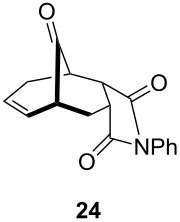 (93%)
2	**19**	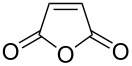	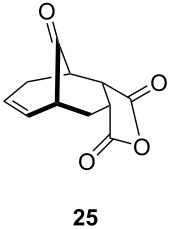 (51%)
3	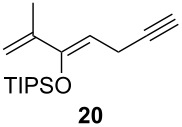	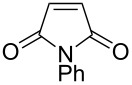	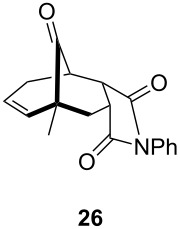 (88%)
4	**20**	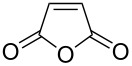	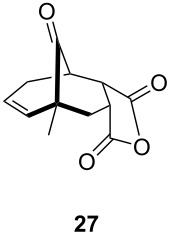 (50%)
5	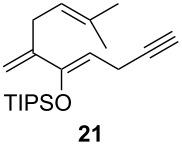	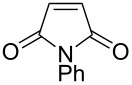	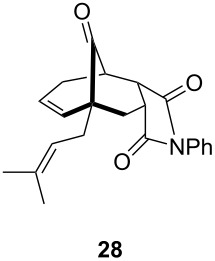 (81%)
6	**21**	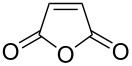	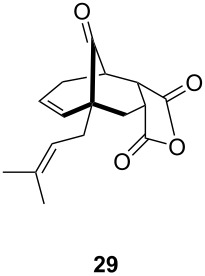 (78%)
7	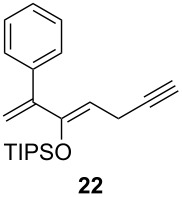	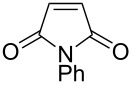	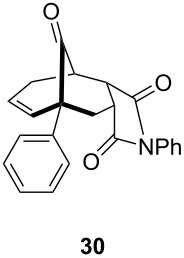 (77%)
8	**22**	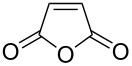	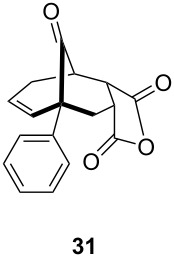 (48%)
9	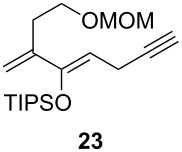	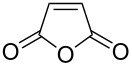	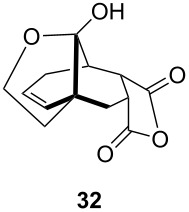 (56%)

^a^Isolated yield and dr > 25:1 in all cases.

One-pot cycloaddition/cyclization of dienes **19** and **20** (*Z*/*E* = 6:1 ca.) with *N*-phenylmaleimide gave ketones **24** and **26** in 93 and 88% yield, respectively, as the sole diastereomers (Table 1, entries 1 and 3). The use of maleic anhydride as the dienophile also provided the desired products **25** and **27**, albeit in lower yields of 51 and 50%, respectively (Table 1, entries 2 and 4). Prenylated diene **21** was smoothly converted to ketones **28** and **29** in 81 and 78% yield, respectively (Table 1, entries 5 and 6). Table 1, entries 7 and 8 reveal that the diene **22**, bearing a phenyl group at C2, can be stereoselectively transformed into the desired bridgehead ketones **30** and **31** in 77 and 48% yields, respectively. Interestingly, hemiketal **32** was isolated in 56% yield, which suggests that the MOM group was cleaved during the Au(I)-catalyzed carbocyclization. It is important to note that the *E*-isomer of dienes **19**–**23** (minor compound) do not react with the dienophiles, but rather isomerized to the *Z*-form under the reaction conditions, thus, ensuring the formation of a single diastereomer.

To extend the scope of the reaction, other dienes possessing internal alkynes were also investigated (Table 2). It can be seen that large substituents at the alkyne terminal position did not affect the efficacy of the reaction. Intermolecular cycloaddition/Au(I)-catalyzed cyclization of aryl acetylene dienes **33**–**35** provided the desired ketones **37**–**39** in yields ranging from 68 to 91% (Table 2, entries 1–3). Remarkably, enyne **36** was converted to **40** in 79% yield (Table 2, entry 4).

**Table 2:** One-pot Diels–Alder cycloaddition/Au(I)-catalyzed carbocyclization of internal alkynes.

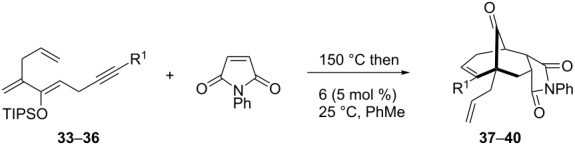			

entry	substituent R^1^	product	yield (%)^a^

1	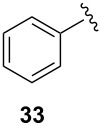	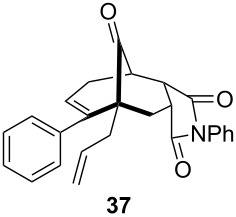	68
2	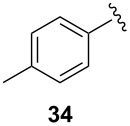	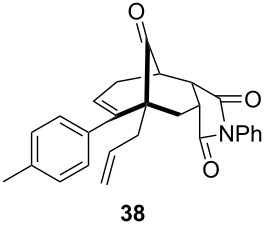	91
3	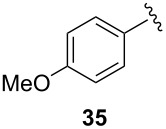	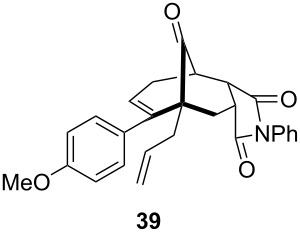	74
4	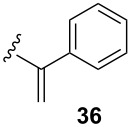	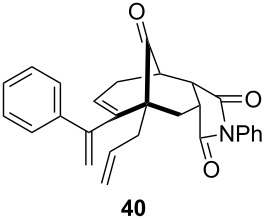	79

^a^Isolated yield and dr >25:1 in all cases.

## Conclusion

In summary, we have developed an efficient stereoselective method for the construction of bicyclic[3.3.1]nonenone frameworks. This one-pot Diels–Alder/Au(I)-catalyzed carbocyclization process provides access to synthetically useful motifs that are found in numerous naturally occurring PPAPs. In addition, the Au(I)-catalyzed cyclization proved to be tolerant of a sterically crowded environment. Further studies to develop an enantioselective version of this reaction and its application to the total synthesis of hyperforin (**2**) and garsubellin A (**3**) are underway and will be reported in due course.

## Supporting Information

File 1 Experimental procedures, characterization data, ^1^H NMR and ^13^C NMR spectra.

File 2 X-ray data of compound **18**.
