# Supplementary material for: One-pot Diels–Alder cycloaddition/gold(I)-catalyzed 6-endo-dig cyclization for the synthesis of the complex bicyclo[3.3.1]alkenone framework
Source: Beilstein J Org Chem. 2011 Jul 22;7:1007–13. doi: 10.3762/bjoc.7.114 (PMC3167837; doi:10.3762/bjoc.7.114)

Supporting Information

for

One-pot Diels–Alder cycloaddition/gold(I)-catalyzed 6-*endo-dig* cyclization for the synthesis of complex bicyclo[3.3.1]alkenone framework

Boubacar Sow, Gabriel Bellavance, Francis Barabé and Louis Barriault*§

Address: Department of Chemistry, 10 Marie Curie, University of Ottawa, Ottawa, Canada, K1N 6N5

Email: Louis Barriault* - lbarriau@uottawa.ca

*Corresponding author
§Phone: 1-613-562-5800; Fax 1-613-562-5170

Experimental procedures and characterization data

General remarks

Unless otherwise indicated, all reactions were performed under either an argon or nitrogen atmosphere in flame-dried glassware equipped with a Teflon coated magnetic stir bar and a rubber septum. Where no temperature is specified, the reactions were run at ambient temperature (23 °C). Reagent quantities (mmol) were calculated based on their reported purities. Anhydrous THF and Et_2O were obtained by distillation over sodium/benzophenone under nitrogen and used as freshly distilled. Et_3N and CH_2Cl_2 were distilled from CaH_2. Commercially available reagents were used as received unless otherwise stated. *n*-Butyllithium and *tert*-butyllithium were titrated using 2,6-di-*tert*-butyl-4-methylphenol and fluorene. Grignard reagents were titrated according to Love's protocol. Microwave reactions were performed

using a CEM Model ESP-1500 Plus microwave oven equipped with a pressure monitoring device and an EST-300 Plus fiber optic temperature probe. The reaction vessel was a quartz tube to which was added the reaction mixture as well as a carboflonTM to aid in the absorption of microwave radiation. Reactions were monitored by TLC analysis using glass plates pre-coated (250 μm thickness) with ultra pure silica gel (60A, SiliCycle). TLC plates were viewed using UV light and stained with either *p*-anisaldehyde, potassium permanganate, or phosphomolybdic acid staining solutions. Flash chromatography was carried out on 230–400 mesh silica gel (60A, SiliCycle). When mentioned, triethylamine was added to the slurry of silica gel until a persistent odor was maintained. Once the basified slurry was loaded on the column, an equal volume of eluent (without triethylamine) was passed through prior to substrate loading.

^{1}H and ^{13}C NMR, spectra were recorded on either Bruker Avance 300 MHz, Bruker Avance 500 MHz, Bruker AMX 500 or Varian INOVA 500 MHz spectrometers in the specified deuterated solvents. IR spectra were recorded on a Bomen Michaelson 100 FTIR spectrometer. HRMS spectra were obtained using a Kratos Analytical Concept spectrometer. Melting points were recorded using a Gallenkamp P1106G Melting Point Apparatus.

Experimental procedures

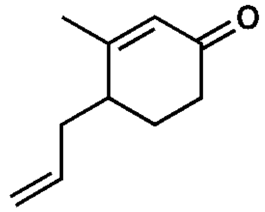

4-Allyl-3-methylcyclohex-2-enone (**8**)

A solution of methyllithium (1.0 M in hexanes, 139.5 mL, 139.5 mmol) was added dropwise to a solution of 6-allyl-3-methoxycyclohex-2-enone (19.31 g, 116.2 mmol) in dry ether at −78 °C for 30 min. The mixture was then stirred at rt for 1 h. An aqueous solution of 1 N HCl was added. After stirring for 1 h at rt, water was added. The

aqueous layer was extracted with ethyl acetate (3x), and the combined organic phases were dried over anhydrous magnesium sulphate, filtered and concentrated. The crude oil was distilled under reduced pressure and purified by chromatography (10% EtOAc:hexanes) to give 14.48 g (83%) of **8** as a clear yellow oil. Spectral data is in accordance with reported data and full characterization is available through the literature [1].

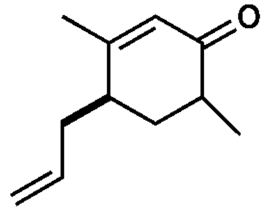

4-Allyl-3,6-dimethylcyclohex-2-enone

A solution of *n*-BuLi (1.6 M in hexanes, 21.8 mL, 34.95 mmol) was added slowly to diisopropylamine (5.17 mL, 36.61 mmol) in THF(150 mL) at −78 °C for 45 min. 4-Allyl-3-methylcyclohex-2-enone (**8**) (5 g, 33.28 mmol) was added at −78 °C for 60 min and then iodomethane (2.49 mL, 39.94 mmol). The mixture was stirred at rt for 3 h. An aqueous saturated solution of NH_4Cl was added and the aqueous layers were extracted with ethyl acetate (3x). The combined organic phases were dried over anhydrous magnesium sulfate, filtered and the solvent was evaporated. The crude residue was purified by flash chromatography (10% EtOAc:hexanes) to give 4.92 g (90%) of a yellow-orange oil of 4-allyl-3,6-dimethylcyclohex-2-enone. IR (neat, cm^{-1}) ν_{max}: 3077, 2964, 2930, 2872, 1673, 1639, 1443, 1378; ^1H NMR, (400 MHz, $CDCl_3$) δ 5.81(m, 1H), 5.79(s, 1H), 5.11(m,2H), 2.43 (m,2H), 2.27 (m,2H), 1.99 (ddd, J = 13.5 Hz, 4.8 Hz, 2.8 Hz, 1H), 1.95(s, 3H), 1.76(m, 1H), 1.09 (d, J = 6.9 Hz, 3H); ^{13}C NMR (400 MHz, $CDCl_3$) δ 201.9 (C), 164.0 (C), 136.5(CH), 126.4 (CH), 117.1 (CH_2), 39.2 (CH), 35.9 (CH), 35.6 (CH_2), 34.5 (CH_2), 22.9 (CH_3), 15.4 (CH_3); HRMS (EI) *m/z* calcd for $C_{11}H_{16}O$ [M^+] 164.1201, found: 164.1205.

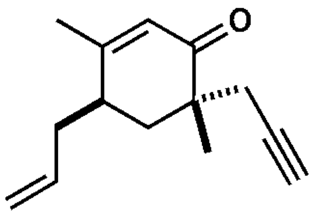

4-Allyl-3,6-dimethyl-6-(prop-2-ynyl)cyclohex-2-enone (**9**)

A solution of *n*-BuLi (1.6 M in hexanes, 1.59 mL, 2.55 mmol) was added slowly to a solution of diisopropylamine (0.38 mL, 2.67 mmol) in THF(15 mL) at −78 °C for 45 min. 4-Allyl-3,6-dimethylcyclohex-2-enone (0.4 g, 2.44 mmol) was added at −78 °C. After stirring for 60 min, propargyl bromide (0.33 mL, 2.92 mmol) was added and the mixture was stirred at rt for 3 h. The resulting mixture was quenched with a saturated solution of NH_4Cl. The aqueous layer was extracted with ethyl acetate (3x). The combined organic phases were dried over anhydrous magnesium sulfate, filtered and concentrated. The crude residue was purified by chromatography (10% EtOAc:hexanes) to give **9** 0.31 g (62%) as a yellow-orange oil. IR (neat, cm^{-1}) ν_{max} 3305, 3077, 2976, 2930, 1674; ^1H NMR, (400 MHz, $CDCl_3$) δ 5.78 (s,1H), 5.69 (m,1H), 5.10 (m,2H), 2.49 (m,2H), 2.35 (m, 1H), 2.15 (m,3H), 2.01 (t, *J* = 2.6 Hz, 1H), 1.93 (s, 3H), 1.57 (dd, *J* = 13.8, 10.3 Hz, 1H), 1.16 (s, 3H); ^{13}C NMR (400 MHz, $CDCl_3$) δ 202.6 (C), 162.9 (C), 135.3 (CH), 126.5 (CH), 118.1 (CH_2), 80.5 (C), 71.4 (CH), 43.9 (C), 38.5 (CH_2), 36.9 (CH), 36.7 (CH_2), 26.8 (CH_2), 22.3 (2xCH_3); HRMS (EI) *m/z* calcd for $C_{14}H_{18}O$ [M^+] 202.1358, found: 202.1379.

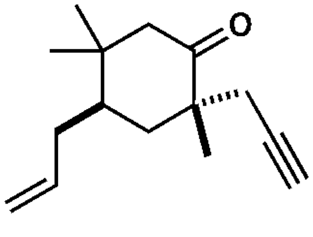

4-Allyl-2,5,5-trimethyl-2-(prop-2-ynyl)cyclohexanone

To a solution of CuI (63 mg, 0.33 mmol) and **9** (306.7 mg, 1.52 mmol) in THF (15 mL) and Me_2S (1.5 mL) at 0 °C was slowly added a solution of MeMgBr (1.11 mL, 3.0 M in Et_2O, 3.33 mmol) over 1 h. The mixture was then stirred for 1 h at 0 °C. An

aqueous saturated solution of NH_4Cl was added. The mixture was extracted with Et_2O (3x). The organic phase was dried over $MgSO_4$ and concentrated in vacuo. The residue was purified by column chromatography (10% EtOAc:hexanes) to provide the desired ketone (175.9 mg, 53%) as a dark orange oil (mixture of two conformers). IR (neat, cm^{-1}) ν_{max} 3310, 3076, 2965, 2931, 2871, 2124, 1716, 1640, 1436; ^1H NMR, (400 MHz, $CDCl_3$) δ 5.76 (m, 1H), 5.02 (m, 2H), 2.49 (0.5H), 2.45 (m, 1H), 2.37 (m, 2H), 2.33 (m, 0.5H), 2.00 (m, 2.5), 1.70 (m, 2.5 H), 1.26 (t, 1H), 1.09 (s,3H), 1.04 (s, 3H), 0.96 (m, 1H), 0.73 (m, 3H); ^{13}C NMR (400 MHz, $CDCl_3$) δ 213.2 (C), 137.6 (CH), 116.3 (CH_2), 79.5 (C), 71.5 (CH), 52.9 (CH_2), 48.0 (C), 41.5 (CH), 40.0 (CH_2), 39.3 (C), 34.2 (CH_2), 29.8 (CH_3), 27.8 (CH_2), 22.2 (CH_3), 20.1 (CH_3); HRMS (EI) *m/z* calcd for $C_{15}H_{22}O$ [M^+] 218.1671, found: 218.1659.

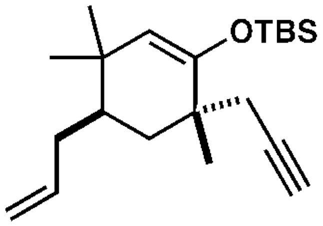

(4-Allyl-3,3,6-trimethyl-6-(prop-2-ynyl)cyclohex-1-enyloxy)(*tert*-butyl)dimethylsilane (**10**)

4-Allyl-2,5,5-trimethyl-2-(prop-2-ynyl)cyclohexanone (300 mg, 1.37 mmol) was dissolved in acetonitrile (30 mL), and Et_3N (0.383 mL, 2.74 mmol) was added. Then flame-dried NaI (0.309 g, 2.04 mmol) and TBSCl (310 mg, 2.04 mmol) were added. The reaction mixture was allowed to reflux overnight and was then quenched with $NaHCO_3$ saturated solution, and the aqueous phase was extracted with DCM (3x). The organic phases were then combined and concentrated. The resulting mixture was filtered through a small silica pad and washed with solution of 7% EtOAc in hexanes and concentrated again. The crude enol ether **10** (209 mg, 46%) was then directly used for the next reaction.

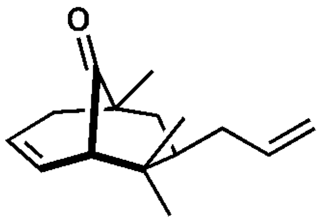

7-Allyl-5,8,8-trimethylbicyclo[3.3.1]non-2-en-9-one(**11**)

A solution of silyl enol ether **10** (20 mg, 0.060 mmol) and (acetonitrile)[(2-biphenyl)di-*tert*-butylphosphine]gold(I) (0.92 mg, 0.0012 mmol) in DCM (1 mL) was stirred for 6 h at rt. The solvent was evaporated and the residue was purified by flash column chromatography on silica (10% EtOAc:hexanes) to give 88% of **11** as a white solid. IR (neat, cm^{-1}) ν_{max} 3081, 3039, 2967, 2921, 1709; ^1H NMR, (400 MHz, $CDCl_3$) δ 5.82 (m, 1H), 5.76 (m, 1H), 5.63 (dddd, *J* = 9.5, 6, 1.9 Hz, 1.9 Hz, 1H), 5.03 (m, 1H), 2.41 (m, 3H), 2.25 (m, 1H), 2.03 (m, 1H), 1.82 (dd, *J* = 13.9 Hz, 4.5 Hz, 1H), 1.59 (ddd, *J* = 13.7, 10.8, 8.6 Hz, 2H), 1.28 (m, 4H), 1.02 (s, 3H), 0.99 (s, 3H), 0.79 (s, 3H); ^{13}C NMR (400 MHz, $CDCl_3$) δ 216.50 (C), 138.03 (CH), 129.80 (CH), 126.55 (CH), 115.96 (CH_2), 60.27 (CH), 45.96 (CH_2), 45.61 (CH_2), 42.35 (C), 38.09 (CH), 34.09 (C), 29.71 (CH_2), 26.09 (CH_3), 23.50 (CH_3), 20.79 (CH_3); HRMS (EI) *m/z* calcd for $C_{15}H_{22}O$ [M+] 218.1671, found: 218.1652.

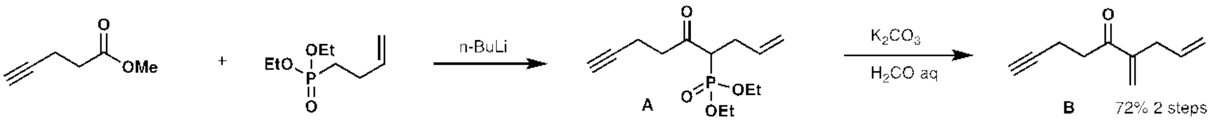

4-Methylenenon-1-en-8-yn-5-one (**B**)

To a solution of homoallylphosphonate (5 g, 26 mmol,) in THF (50 mL) at −78 °C, under an argon atmosphere, was added dropwise a solution of *n*-BuLi (2.45 M in hexanes, 12.74 mL, 31.2 mmol). The reaction mixture was stirred for 3 h and then a solution of methyl pent-4-ynoate (4.38 g, 39 mmol) in THF (10mL) was added to the prepared mixture. After 1 h, the temperature was raised to rt. The reaction was quenched with a saturated solution of NH_4Cl. The mixture was extracted three times with Et_2O, washed with water and concentrated at reduced pressure to furnish the

diethyl 5-oxonon-1-en-8-yn-4-ylphosphonate (**A**) as a brown oil. The crude product was taken into the next step without further purification.

A mixture of diethyl 5-oxonon-1-en-8-yn-4-ylphosphonate **A** (7.0 g, 24.4 mmol), water (100 mL), potassium carbonate (10.1 g, 73.3 mmol) and aqueous 37% formaldehyde (2.18 mL, 29.3 mmol) was stirred at rt for 3 h. The mixture was then extracted with Et_2O, washed with water, saturated solution of NaCl and concentrated under reduced pressure to furnish a yellow oil. Purification by flash chromatography over silica gel (hexanes/EtOAc in gradient 5–10% of EtOAc) gave the 4-methylenenon-1-en-8-yn-5-one **B** (2.6 g, 72%) as a colorless oil. IR (neat, cm^{-1}) ν_{max} 3300, 2919, 2120, 1642, 1628, 1415. ^1H NMR (300 MHz, $CDCl_3$) δ 6.06 (s, 1 H), 5.87 - 5.70 (m, 2 H), 5.09 - 5.05 (m, 1 H), 5.05 - 4.99 (m, 1 H), 3.04 - 2.98 (m, 2 H), 2.97 - 2.90 (m, 2 H), 2.52 - 2.42 (m, 2 H), 1.94 (t, J = 2.7 Hz, 1 H). ^{13}C NMR (75 MHz, $CDCl_3$) δ 198.8, 146.8, 135.3, 125.2, 117.0, 83.4, 68.8, 36.7, 34.9, 13.4. HRMS (ESI) *m/z* cald for $C_{10}H_{12}ONa^+$ 148.0888, found: 148.1206.

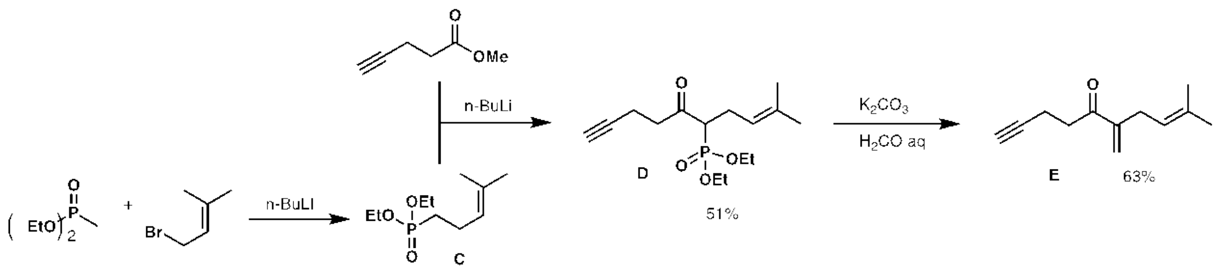

9-Methyl-6-methylenedec-8-en-1-yn-5-one (**E**)

To a solution of diethyl 4-methylpent-3-enylphosphonate [2] **C** (2.43 g, 11.03 mmol) in THF (20 mL) at −78 °C, under an argon atmosphere, was added dropwise *n*-BuLi (1.7 M in hexanes, 7.79 mL, 13.24 mmol). The reaction mixture was stirred for 3 h and then a solution of methyl pent-4-ynoate (1.86 g, 16.5 mmol) in THF (5 mL) was added. After stirring for 1 h, the temperature was raised to rt. The reaction was quenched with a saturated solution of NH_4Cl. The mixture was extracted three times with Et_2O, washed with water and concentrated at reduced pressure to furnish the diethyl 5-oxonon-1-en-8-yn-4-ylphosphonate **D** as a brown oil. The crude product was taken into the next step without further purification.

A mixture of diethyl 2-methyl-6-oxodec-2-en-9-yn-5-ylphosphonate **D** (1.7 g, 5.66 mmol, 1 equiv), water (25 mL), potassium/carbonate (2.35 g, 17 mmol, 3 equiv) and aqueous 37% formaldehyde (0.46 mL, 6.23 mmol, 1.1 equiv) was stirred at rt for 3 h. The mixture was then extracted with Et_2O, washed with water and a sat. solution of NaCl and concentrated at reduced pressure to furnish a yellow oil. Purification by flash chromatography over silica gel (Hexanes/EtOAc in gradient 5 - 10% of EtOAc) gave the 9-methyl-6-methylenedec-8-en-1-yn-5-one **E** (0.63 g, 63%) as colorless oil. IR (neat, cm^{-1}) ν_{max} 3295, 2968, 2918, 2124, 1680, 1623. ^1H NMR (300 MHz ,$CDCl_3$) δ 6.01 (s, 1 H), 5.75 (dt, *J* = 1.6 Hz, 0.3, 1 H), 5.12 (sptt, *J* = 1.4, 7.3 Hz, 1 H), 2.95 (d, *J* = 7.6 Hz, 2 H), 2.97 (t, *J* = 6.4 Hz, 2 H), 2.56 - 2.43 (m, 2 H), 1.95 (t, *J* = 2.7 Hz, 1H), 1.73 (s, 3 H), 1.61 (s, 3 H). ^{13}C NMR (75 MHz, $CDCl_3$) δ 199.4, 147.6, 134.4, 124.4, 120.7, 83.6, 68.8, 36.9, 29.3, 25.9, 17.8, 13.5. HRMS (ESI) m/z calcd for $C_{12}H_{16}ONa^+$ 176.1201, found: 176.1196.

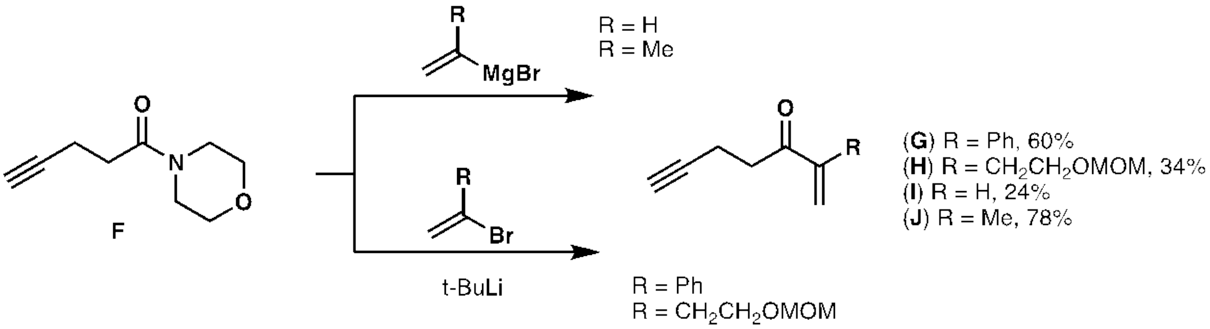

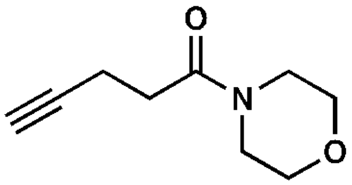

1-Morpholinopent-4-yn-1-one (**F**)

Pent-4-ynoic acid (17.5 g, 176 mmol), morpholine (12.9 mL, 149 mmol) and DMAP (1.82 g, 149 mmol) were dissolved in DCM (325 mL). The solution was cooled to 0 °C and DCC (36.8 g, 178 mmol) was added slowly and the solution warmed up to rt. The reaction was stirred overnight. The resulting mixture is filtered over silica to remove 1,3-dicyclohexylurea, which was insoluble in DCM, and then washed with DCM. The filtrate was concentrated and the residue was purified by flash chromatography (40% EtOAc/hexanes). The yellow solid was then recrystallized from

diisopropyl ether to afford 1-morpholinopent-4-yn-1-one **F** (19 g, 0.114 mol) a fluffy white solid in 85% yield. IR (neat, cm^{-1}) ν_{max} 3250, 2925, 2857, 1644, 1436; ^{1}H NMR (400 MHz, $CDCl_3$) δ ppm 2.0 (m, 1 H), 2.5 (d, J = 1.2 Hz, 4 H), 3.5 (m, 2 H), 3.6 (m, 6 H); ^{13}C NMR (101 MHz, $CDCl_3$) δ ppm 169.6(C), 83.4(C), 68.9 (CH), 67.0 (CH_2), 66.6 (CH_2), 45.9 (CH_2), 42.1(CH_2), 32.0 (CH_2), 14.5 (CH_2); HRMS (EI) *m/z* calculated for $C_9H_{13}NO_2$ [M+] 167.0946, found: 167.0934. mp = 81–82 °C.

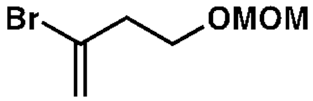

2-Bromo-4-(methoxymethoxy)butene

Methoxymethyl chloride (7.21 mL, 95 mmol) was added to 3-bromobut-3-en-1-ol (3.14 mL, 31.7 mmol) in DCM (150 mL). N,N-diisopropylethylamine (22.6 mL, 158 mmol) was added to the mixture. The resulting mixture was stirred at rt for 3 h after which all the starting material was consumed. The solution was concentrated and the residue was purified by chromatography using 10% EtOAc in hexanes to afford 2-bromo-4-(methoxymethoxy)but-1-ene as a clear oil in 92% yield. IR (neat, cm^{-1}) ν_{max} 2925, 2852, 2336, 1729, 1279; ^{1}H NMR (400 MHz, $CDCl_3$) δ ppm 2.69 (td, J = 6.3, 0.8 Hz, 2 H) 3.36 (s, 3 H) 3.72 (t, J = 6.3 Hz, 2 H) 4.63 (s, 2 H) 5.49 (d, J = 1.6 Hz, 1 H) 5.68 (q, J = 1.2 Hz, 1 H); ^{13}C NMR (101 MHz, $CDCl_3$) δ ppm 41.7 (CH_2), 55.3 (CH_3), 65.1 (CH_2), 96.5 (CH_2), 118.5 (CH_2), 130.7 (C); HRMS (EI) *m/z* calcd for C_4H_4OBr [M+ (-CH_2OCH_3)] 148.9602, found: 149.0249.

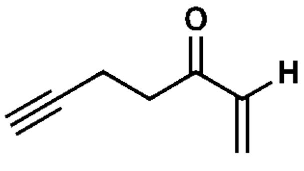

Hept-1-en-6-yn-3-one (**I**)

A solution of 1-morpholinopent-4-yn-1-one (4.0 g, 23.92 mmol) in THF (159 mL) was cooled to 0 °C and vinylmagnesium bromide 1 M in THF (60 mL, 59.8 mmol) was added dropwise. The solution was warmed to 23 °C and stirred overnight. The

resulting mixture was quenched with a saturated solution of NH_4Cl. The aqueous phase was extracted with Et_2O (3x), the combined organic phases were dried over $MgSO_4$ and concentrated at 25 °C. The crude was purified by flash chromatography at 10% Et_2O in hexanes to afford hept-1-en-6-yn-3-one **I** (0.7 g, 27%) as a clear oil. IR (neat, cm^{-1}) ν_{max} 3524, 3296, 3091, 2925, 2121 1934, 1688; ^{1}H NMR (400 MHz, $CDCl_3$) δ ppm 1.95 (t, J = 2.7 Hz, 1 H) 2.51 (m, 2 H) 2.85 (m, 2 H) 5.87 (dd, J = 10.4, 1.2 Hz, 1 H) 6.24 (dd, J=17.7, 1.1 Hz, 1 H) 6.35 (dd, J = 20.7, 7.3 Hz, 1 H); ^{13}C NMR (101 MHz, $CDCl_3$) δ ppm 12.9 (CH_2), 38.3 (CH_2), 68.7 (CH), 83.1 (C), 128.7 (CH_2), 136.2 (CH), 198.2 (C); HRMS (EI) *m/z* calcd for C_7H_8O [M+] 108.0575, found: 108.0545.

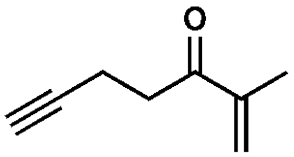

2-Methylhept-1-en-6-yn-3-one (**J**)

A solution of 1-morpholinopent-4-yn-1-one (4 g, 23.92 mmol) in THF (159 mL) was cooled to 0 °C and a solution of isopropenylmagnesium bromide 0.5 M in THF (120 mL, 59.8 mmol) was added dropwise. The solution was stirred overnight at rt. The resulting mixture was quenched with a saturated solution of NH_4Cl, extracted with Et_2O (3x), dried over $MgSO_4$ and concentrated. The crude was purified by flash chromatography (10% EtOAc/hexanes) to afford 2-methylhept-1-en-6-yn-3-one **J** (3 g, 85%) as a clear oil. IR (neat, cm^{-1}) ν_{max} 3303, 3098, 2963, 2925, 2114, 1688; ^{1}H NMR (400 MHz, $CDCl_3$) δ ppm 1.85 (dd, J = 1.4, 0.9 Hz, 3 H), 1.93 (t, J = 2.6 Hz, 1H), 2.47 (ddd, J = 8.2, 6.6, 2.6 Hz, 2 H), 2.93 (m, 2 H), 5.78 (d, J = 1.1 Hz, 1H), 5.96 (s, 1 H); ^{13}C NMR (101 MHz, $CDCl_3$) δ ppm 13.3 (CH_3), 17.4 (CH_2), 36.4 (CH_2), 68.6 (CH), 83.3(C), 124.9(CH_2), 144.1 (C), 199.3 (C); HRMS (EI) *m/z* calcd for $C_8H_{10}O$ [M+] 122.0732, found: 122.0695.

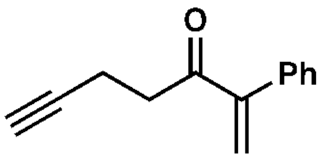

2-Phenylhept-1-en-6-yn-3-one (**G**)

A solution of (1-bromovinyl)benzene (90%, 3.83 g, 18.84 mmol) in THF (90 mL) was cooled to −78 °C followed by the addition of *t*-BuLi 1.7 M in pentane (21.2 mL, 37.7 mmol). The mixture was stirred for 30 min which after a solution of 1-morpholinopent-4-yn-1-one (3 g, 17.9 mmol) in THF (10 mL) was added through a cannula to the reaction mixture. After stirring for 2 h, the resulting mixture was quenched with a saturated solution of NH_4Cl and then extracted with Et_2O (3x). The combined organic phases were dried over $MgSO_4$ and concentrated. The residue was purified by flash chromatography (10% EtOAc/hexanes) to afford 2-phenylhept-1-en-6-yn-3-one **G** (2 g, 60%) as a pale yellow oil. IR (neat, cm^{-1}) ν_{max} 3295, 3057, 3026, 2921, 2119, 1686; ^1H NMR (400 MHz, $CDCl_3$) δ ppm 1.96 (t, *J* = 2.7 Hz, 1 H), 2.54 (m, 2 H), 3.00 (m, 2H), 5.92 (s, 1 H), 6.15 (s, 1 H), 7.25 (dt, *J* = 5.3, 2.1 Hz, 2 H), 7.34 (m, 3 H); ^{13}C NMR (101 MHz, $CDCl_3$) δ ppm 13.4 (CH_2), 38.5 (CH_2), 68.8 (CH), 83.1 (C), 125.0 (CH_2), 128.2 (CH), 128.3 (CH x 2), 128.4 (CH x 2), 136.9 (C), 149.0 (C), 199.4 (C); HRMS (EI) *m/z* calculated for $C_{13}H_{12}O$ [M+] 184.0888, found: 184.0845.

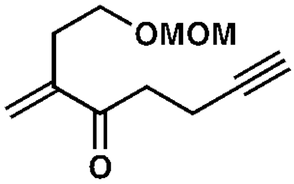

1-(Methoxymethoxy)-3-methyleneoct-7-yn-4-one (**H**)

A solution of 2-bromo-4-(methoxymethoxy)but-1-ene (0.392 g, 2 mmol) in THF (5 mL) was cooled to −78 °C followed by the addition of *t*-BuLi 1.7 M in pentane (2.41 mL, 4.11 mmol). After stirring for 30 min, a solution of 1-morpholinopent-4-yn-1-one (0.16 g, 0.957 mmol) in THF (3 mL) was added through a cannula and the mixture was stirred for 2 h. The reaction was quenched with a saturated solution of NH_4Cl. The mixture was extracted with Et_2O (3x), dried over $MgSO_4$ and then concentrated. The crude residue was purified by flash chromatography (10–15% EtOAc/hexanes) to afford 1-(methoxymethoxy)-3-methyleneoct-7-yn-4-one **H** (0.645 g, 34%) as a

clear oil. IR (neat, cm^{-1}) ν_{max} 3287, 2932, 2885, 2114, 1679; ^{1}H NMR (400 MHz, $CDCl_3$) δ ppm 1.94 (t, *J* = 2.7 Hz, 1 H), 2.50 (ddd, *J* = 8.2, 6.5, 2.7 Hz, 2 H), 2.59 (td, *J* = 6.5, 0.9 Hz, 2 H), 3.96 (m, 2 H), 3.33 (s, 3 H), 3.61 (t, *J*=6.6 Hz, 2 H), 4.58 (s, 2 H), 5.89 (t, *J*=1.2 Hz, 1 H), 6.09 (s, 1 H); ^{13}C NMR (101 MHz, $CDCl_3$) δ ppm 13.3 (CH_2), 31.3 (CH_2), 36.6 (CH_2), 55.2 (CH_3), 66.1 (CH_2), 68.7 (CH), 83.3 (C), 96.4 (CH_2), 126.0 (CH_2), 145.4 (C), 199.0 (C); HRMS (EI) *m/z* calcd for $C_9H_{11}O_2$ [M+ (-CH_2OCH_3)] 151.0759, found: 151.0758.

General procedure for the formation of dienes 16, 19–22 and 32–35

To a solution of enone (1 mmol) in DCM (5 mL) was added Et_3N (3 mmol) and TIPSOTf (2 mmol). The solution was then heated at reflux overnight. The reaction was quenched with a saturated solution of $NaHCO_3$. The aqueous phase was extracted with DCM (2x), the organic phases were combined and dried over $MgSO_4$. The solution was concentrated and the residue was purified by flash chromatography (1% EtOAc:hexanes or 5% Et_2O:hexanes) to give the corresponding diene as a mixture of *Z/E* isomers ranging from 4 to 9:1.

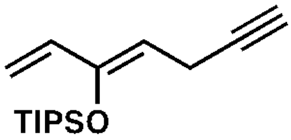

(*Z*)-(Hepta-1,3-dien-6-yn-3-yloxy)triisopropylsilane (**19**)

Yield 24%; IR (neat, cm^{-1}) ν_{max} 3313, 2946, 2868, 2121, 1647; ^{1}H NMR (400 MHz, $CDCl_3$) δ ppm 1.10 (d *J* = 6.66 Hz, 18 H), 1.20 (m, 3 H), 1.94 (t, *J* = 2.7 Hz, 1 H), 3.06 (dd, *J*=7.1, 2.7 Hz, 2 H), 4.82 (t, *J* = 7.1 Hz, 1 H), 5.03 (dd, *J* = 10.8, 1.0 Hz, 1 H), 5.38 (dd, *J* = 17.2, 1.0 Hz, 1 H), 6.14 (dd, *J* = 17.2, 10.8 Hz, 1 H); ^{13}C NMR (101 MHz, $CDCl_3$) δ ppm 13.7 (CH x 3), 15.9 (CH_2), 18.0 (CH_3 x 6), 67.7 (CH), 82.9 (C), 106.9 (CH), 113.7 (CH_2), 135.3 (CH), 150.5 (C); HRMS (EI) *m/z* calcd for $C_{16}H_{28}OSi$ [M+] 264.1909, found: 264.1921

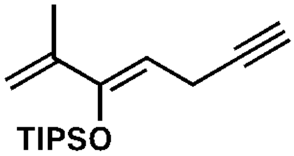

(Z)-Triisopropyl(2-methylhepta-1,3-dien-6-yn-3-yloxy)silane (**20**)

Yield 78%; IR (neat, cm^{-1}) ν_{max} 3314, 2947, 2866, 2124, 1612, 1464; ^{1}H NMR (400 MHz, $CDCl_3$) δ ppm 1.10 (d, J = 6.6 Hz, 18H), 1.20(m, 3H), 1.87(m, 3H), 1.95 (t, J = 2.7 Hz, 1H), 3.07 (dd, J = 6.9, 2.74 Hz, 2H), 4.89 (t, J = 6.9 Hz, 1H), 4.92 (m, 1H), 5.26 (m, 1H); ^{13}C NMR (101 MHz, $CDCl_3$) δ ppm 13.9 (CH x3), 15.9 (CH_2), 18.0 (CH_3 x6), 20.3 (CH_3), 67.7 (CH), 83.1 (C), 104.7 (CH), 113.0 (CH_2), 140.7 (C), 152.0 (C); HRMS (EI) m/z calcd for $C_{17}H_{30}OSi$ [M+] 278.2066, found: 278.2066.

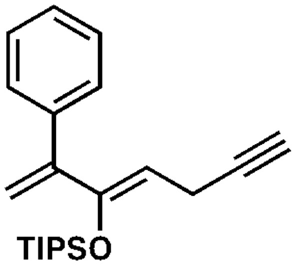

(Z)-Triisopropyl(2-phenylhepta-1,3-dien-6-yn-3-yloxy)silane (**22**)

Yield 18%; IR (neat, cm^{-1}) ν_{max} 3311, 3082, 3060, 3026, 2945, 2867, 2120, 1948, 1875, 1799, 1720; ^{1}H NMR (400 MHz, $CDCl_3$) δ ppm 1.00 (d, J = 6.1 Hz, 18 H), 1.09 (m, 3 H), 1.93 (t, J = 2.7 Hz, 1 H), 3.09 (dd, J = 6.9, 2.7 Hz, 2 H), 4.86 (t, J = 6.9 Hz, 1 H), 5.25 (d, J = 1.6 Hz, 1 H), 5.42 (d, J = 1.6 Hz, 1 H), 7.31 (m, 3 H), 7.37 (m, 2 H); ^{13}C NMR (101 MHz, $CDCl_3$) δ ppm 13.5 (CH x 3), 15.7 (CH_2), 17.9 (CH_3 x 6), 67.7 (CH), 83.0 (C), 108.3 (CH), 114.2 (CH_2), 127.7 (CH), 128.1 (CH x 2), 128.1 (CH x 2), 139.3 (C), 147.9 (C), 151.7 (C); HRMS (EI) m/z calcd for $C_{22}H_{32}OSi$ 340.2222 [M+], found: 340.2225

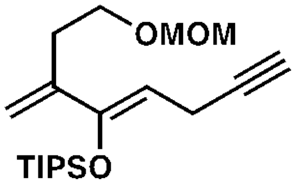

(*Z*)-8-(But-3-ynylidene)-10,10-diisopropyl-11-methyl-7-methylene-2,4,9-trioxa-10-siladodecane (**23**)

Yield 64%; IR (neat, cm^{-1}) ν_{max} 3400, 3311, 2945, 2868, 2100, 1698, 1618, 1464; ^{1}H NMR (300 MHz, $CDCl_3$) δ ppm 1.09 (d, *J* = 6.1 Hz, 18 H), 1.18 (m, 3 H), 1.94 (t, *J* = 2.7 Hz, 1 H), 2.52 (td, *J* = 7.1, 0.9 Hz, 2 H), 3.05 (dd, *J* = 6.9, 2.8 Hz, 2 H), 3.36 (s, 3 H), 3.65 (t, *J* = 7.2 Hz, 2 H), 4.62 (s, 2 H), 4.91 (t, *J* = 6.9 Hz, 1 H), 4.97 (d, *J* = 1.2 Hz, 1 H), 5.32 (d, *J* = 1.4 Hz, 1 H); ^{13}C NMR (101 MHz, $CDCl_3$) δ ppm 13.8 (CH x 3), 15.8 (CH_2), 18.0 (CH_3 x 6), 33.6 (CH_2), 55.2 (CH_3), 66.6 (CH_2), 67.8 (CH), 83.0 (C), 96.5 (CH_2), 104.8 (CH), 113.7 (CH_2), 142.5 (C), 151.3 (C); HRMS (EI) *m/z* calcd for $C_{20}H_{36}O_3Si$ [M+ ($CH(CH_3)_2$)] 309.1886, found: 309.1993.

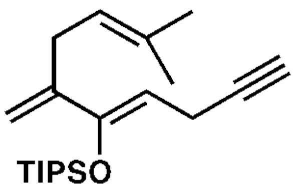

(*Z*)-Triisopropyl(9-methyl-6-methylenedeca-4,8-dien-1-yn-5-yloxy)silane (**21**)

To a solution of 9-methyl-6-methylenedec-8-en-1-yn-5-one **E** (560 mg, 3.18 mmol) in DCE (30 mL) at rt, under an argon atmosphere, was added Et_3N (2.23 mL, 15.9 mmol) and then TIPSOTf (2.56 mL, 9.53 mmol). The reaction mixture was stirred for 8h at reflux. The reaction was quenched with a saturated solution of $NaHCO_3$. The mixture was extracted two times with DCM, washed with water, dried over $MgSO_4$ and concentrated at reduced pressure to furnish a brown oil. Purification by flash chromatography over silica gel (hexanes/EtOAc in gradient 1 - 5% of EtOAc) gave **21** (962 mg, 91%) as yellow oil. IR (neat, cm^{-1}) ν_{max} 3315, 2962, 2945, 2868, 2121, 1549; ^{1}H NMR (300 MHz,$CDCl_3$) δ 5.29 (d, *J* = 1.6 Hz, 1 H), 5.23 - 5.14 (m, 1H), 4.95 - 4.86 (m, 1 H), 4.90 (t, *J* = 7.1 Hz, 1 H), 3.07 (dd, *J* = 2.7, 6.9 Hz, 2 H), 2.97 - 2.83 (m, 2 H), 1.95 (t, *J* = 2.7 Hz, 1 H), 1.72 (d, *J* = 1.1 Hz, 3 H), 1.62 (d, *J* = 0.9 Hz, 3 H); ^{13}C NMR (75 MHz, $CDCl_3$) δ 151.8, 144.6, 133.3, 121.8, 112.5, 104.7, 83.4, 67.8, 32.1, 25.9, 18.3 16.0; HRMS (EI) *m/z* calcd for $C_{18}H_{29}OSi$ [M+ (-C_3H_7)] 289.1988, found: 289.2169.

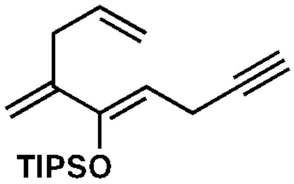

(Z)-Triisopropyl(6-methylenenona-4,8-dien-1-yn-5-yloxy)silane (**16**)

To a solution of 4-methylenenon-1-en-8-yn-5-one **B** (300 mg, 2.02 mmol) in DCE (20 mL) at rt, under an argon atmosphere, was added Et_3N (1.42 mL, 10.1 mmol) and then TIPSOTf (1.63 mL, 6.07 mmol). The reaction mixture was stirred for 8 h at reflux. The reaction was quenched with a saturation of solution of $NaHCO_3$. The mixture was extracted two times with DCM, washed with water, dried over $MgSO_4$ and concentrated at reduced pressure to furnish a brown oil. Purification by flash chromatography over silica gel (hexanes/EtOAc in gradient 1–5% of EtOAc) gave **16** (469 mg, 76%) as a yellow oil. IR (neat, cm^{-1}) ν_{max} 3314, 2945, 2868, 2121; ^{1}H NMR (400 MHz, $CDCl_3$) δ 5.85 (ddt, J = 16.9, 10.1, 6.9 Hz, 1 H), 5.34 (d, J = 1.3 Hz, 1 H), 5.12 - 5.03 (m, 2 H), 4.96 (d, J = 1.2 Hz, 1 H), 4.92 (t, J = 6.9 Hz, 1 H), 3.07 (dd, J = 6.9, 2.7 Hz, 2 H), 2.96 (dd, J = 6.8, 0.9 Hz, 2 H), 1.95 (t, J = 2.7 Hz, 1 H), 1.25 - 1.15 (m, 3 H), 1.12 - 1.09 (m, 18 H). ^{13}C NMR (75 MHz, $CDCl_3$) δ 151.5, 144.0, 136.2, 116.5, 113.2, 105.0, 83.2, 67.9, 37.8, 18.3, 18.2, 16.0, 14.0, 13.6; HRMS m/z calcd for $C_{16}H_{25}OSi$ [M+ (-C_3H_7)] 261.1675, found: 261.1695.

General procedure for the Diels–Alder reaction/gold cyclization process

Diene (1 equiv) was charged into a microwave quartz tube (the tube was washed in a base bath and dried). N-phenylmaleimide (2 equiv) was added and then toluene (0.1 M) was added. The mixture was heated to 150 °C at 300 W in the microwave for 2 h. After cooling down to rt, catalyst **6** (5 mol%) was added to the mixture with a minimal amount of acetone to solubilize the catalyst. After stirring overnight, the

solution was concentrated and purified by flash chromatography (25–40% EtOAc in hexanes) to afford the desired ketone.

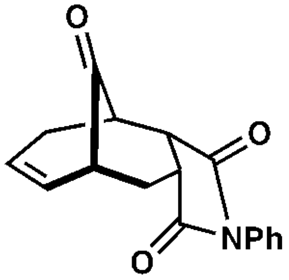

4-Phenyl-4-azatricyclo[6.3.1.$0^{2,6}$]dodec-9-ene- 3,5,12-trione (**24**)

Yield 93% (35 mg); IR (neat, cm^{-1}) ν_{max} 3483, 3074, 3036, 2930, 2864, 1711; ^{1}H NMR (400 MHz, $CDCl_3$) δ ppm 2.33 (ddd, *J* = 14.5, 7.9, 4.2 Hz, 1 H), 2.64 (t, *J* = 3.1 Hz, 2 H), 2.95 (m, *J* = 2.5 Hz, 1 H), 3.03 (m, 3 H), 3.68 (t, *J* = 8.9 Hz, 1 H), 5.72 (m, 1 H), 5.82 (dt, *J* = 9.6, 3.4 Hz, 1 H), 7.27 (m, 2 H), 7.39 (m, 1 H), 7.50 (m, 2 H); ^{13}C NMR (101 MHz, $CDCl_3$) δ ppm 31.0 (CH_2), 32.6 (CH_2), 36.3 (CH), 44.0 (CH), 45.7 (CH), 47.5 (CH), 126.1 (CH x2), 127.0 (CH), 128.8 (CH), 129.3 (CH x2), 130.2 (CH), 131.7 (C), 175.6 (C), 177.4 (C), 212.1 (C); HRMS (EI) *m/z* calcd for $C_{17}H_{15}NO_3$ [M+] 281.1052, found: 281.1061; mp = 155–160 °C.

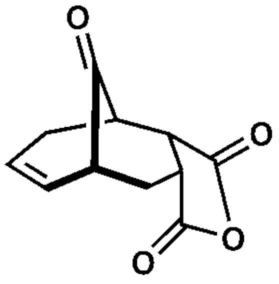

4-Oxatricyclo[6.3.1.$0^{2,6}$]dodec-9-ene-3,5,12- trione (**25**)

Yield 51% (11 mg); IR (neat, cm^{-1}) ν_{max} 2926, 2854, 1857, 1700, 1447; ^{1}H NMR (400 MHz, $CDCl_3$) δ ppm 2.36 (ddd, *J*=14.6, 7.5, 4.2 Hz, 1 H), 2.57 (m, 1 H), 2.70 (m, 1 H), 2.87 (dd, *J* = 14.6, 2.1 Hz, 1 H), 2.97 (m, 2 H), 3.18 (ddd, *J* = 9.9, 7.7, 1.0 Hz,

1H), 3.74 (t, 1 H), 5.69 (ddd, *J*=9.5, 4.9, 3.0 Hz, 1 H), 5.82 (m, *J* = 9.6, 4.9, 2.1 Hz, 1H); ^{13}C NMR (101 MHz, $CDCl_3$) δ ppm 30.9 (CH_2), 33.0 (CH_2), 36.5 (CH), 43.2 (CH), 45.2 (CH), 48.0 (CH), 127.7 (CH), 129.6 (CH), 170.1 (C), 172.5 (C), 210.1 (C); HRMS (EI) *m/z* calcd for $C_{11}H_{10}O_4$ [M+] 206.0579, found: 206.0547; mp = 132–135 °C.

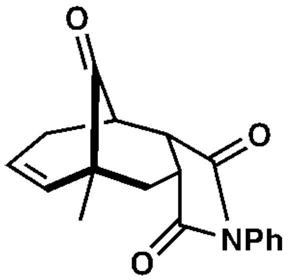

8-Methyl-4-phenyl-4-azatricyclo[6.3.1.0^{2,6}]dodec-9-ene-3,5,12-trione (**26**)

Yield 88% (75 mg); IR (neat, cm^{-1}) ν_{max} 2960, 2924, 2854, 1709, 1377; ^{1}H NMR (400 MHz, $CDCl_3$) δ ppm 1.17 (s, 3 H), 2.4 (dd, *J* = 14.4, 7.7 Hz, 1 H), 2.62 (m, 2 H), 2.90 (d, *J* = 14.3 Hz, 1 H), 3.00 (ddd, *J* = 9.0, 7.8, 0.9 Hz, 1 H), 3.11 (m, 1 H), 3.68 (t, *J* = 9.0 Hz, 1 H), 5.50 (ddd, *J* = 9.5, 2.1, 1.1 Hz, 1 H), 5.75 (m, 1 H), 7.26 (m, 2 H), 7.42 (m, 1 H), 7.50 (m, 2 H); ^{13}C NMR (101 MHz, $CDCl_3$) δ ppm 20.5 (CH_3), 32.6 (CH_2), 37.7 (CH), 38.4 (CH_2), 44.4 (CH), 46.1 (C), 47.4 (CH), 125.8 (CH), 126.1 (CH x2), 128.8 (CH), 129.3 (CH x2), 131.7 (C), 135.8 (CH), 175.7 (C), 177.4 (C), 212.8 (C); HRMS (EI) *m/z* calcd for $C_{18}H_{17}NO_3$ [M+] 295.1208, found: 295.1228; mp = 189–192 °C.

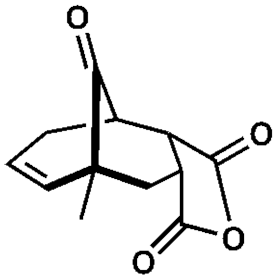

8-Methyl-4-oxatricyclo[6.3.1.0^{2,6}]dodec-9-ene- 3,5,12-trione (**27**)

Yield, 50% (30 mg); IR (neat, cm^{-1}) ν_{max} 2928, 2866, 2138, 1860, 1455; ^{1}H NMR (400 MHz, $CDCl_3$) δ ppm 1.13 (s, 3 H), 2.06 (dd, *J* = 14.5, 7.6 Hz, 1 H), 2.59 (ddd, *J* = 19.1, 4.9, 1.9 Hz, 1 H), 2.68 (m, 1 H,) 2.71 (d, *J* = 14.5 Hz, 1 H), 3.03 (m, 1 H), 3.19

(ddd, *J* = 9.9, 7.5, 1.0 Hz, 1 H), 3.73 (dd, *J* = 9.7, 8.9 Hz, 1 H), 5.46 (dd, *J* = 9.4, 2.9 Hz, 1 H), 5.76 (m, 1 H); ^{13}C NMR (101 MHz, $CDCl_3$) δ ppm 20.2 (CH_3), 33.0 (CH_2), 38.0 (CH), 38.2 (CH_2), 43.5 (CH), 45.9 (C), 47.9 (CH), 126.4 (CH), 135.3 (CH), 170.2 (C), 172.5 (C), 210.9 (C); HRMS (EI) *m/z* calcd for $C_{12}H_{12}O_4$ [M+] 220.0736, found: 220.0717; mp=101-105˚C.

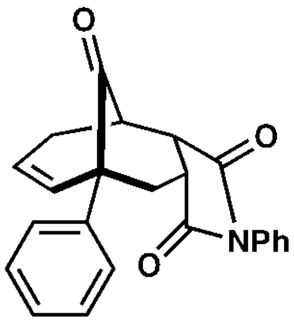

4,8-Diphenyl-4-azatricyclo[6.3.1.0^{2,6}]dodec-9-ene-3,5,12-trione (**30**)

Yield 77% (24 mg); IR (neat, cm^{-1}) ν_{max} 3061, 3030, 2926, 2855, 1781; 1H NMR (400 MHz, $CDCl_3$) δ ppm 2.80 (m, 3 H) 3.26 (m, 3 H) 3.80 (t, *J* = 9.2 Hz, 1 H) 5.71 (dd, *J* = 9.6, 2.5 Hz, 1 H) 5.98 (m, 1 H) 7.21 (m, 2 H) 7.32 (m, 3 H) 7.38 (m, 3 H) 7.52 (m, 2H); 13C NMR (101 MHz, $CDCl_3$) δ ppm 32.0 (CH_2) 34.9 (CH_2) 37.9 (CH) 45.3 (CH) 47.2 (CH) 54.5 (C) 126.1 (CHx2) 126.5 (CH) 127.4 (CHx2) 127.6 (CH) 128.4 (CHx2) 128.8 (CH) 129.4 (CHx2) 131.8 (C) 135.2 (CH) 138.7 (C) 175.5 (C) 177.3 (C) 210.5 (C); HRMS (EI) *m/z* calculated for $C_{23}H_{19}NO_3$ 357.1365, found 357.1358; mp = 221–224 °C

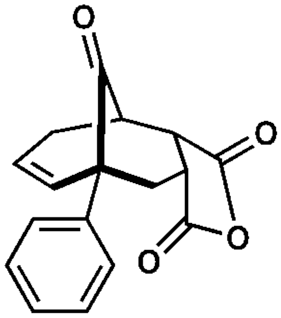

8-Phenyl-4-oxatricyclo[6.3.1.0^{2,6}]dodec-9-ene-3,5,12-trione (**31**)

Yield 48% (18 mg); IR (neat, cm^{-1}) ν_{max} 2980, 2928, 2901, 2859, 1851, 1778, 1724; ^{1}H NMR (400 MHz, $CDCl_3$) δ ppm 2.71 (ddd, *J* = 19.1, 5.3, 1.9 Hz, 1 H), 2.83 (dd, *J* =

14.2, 7.5 Hz, 1 H), 2.90 (m, 1H), 3.10 (d, *J* = 13.7 Hz, 1 H), 3.18 (m, 1 H), 3.40 (ddd, *J* = 10.1, 7.5, 0.8 Hz, 1 H), 3.85 (m, 1 H), 5.71 (dd, *J* = 9.6, 3.1 Hz, 1 H), 6.02 (m, 1H), 7.18 (m, 2 H), 7.33 (m, 1 H), 7.38 (m, 2 H); ^{13}C NMR (101 MHz, $CDCl_3$) δ ppm 32.4 (CH_2), 34.9 (CH_2), 38.3 (CH), 44.5 (CH), 47.7 (CH), 54.3 (C), 127.2 (CH), 127.3 (CH x2), 127.9 (CH), 128.5 (CH x2), 134.9 (CH), 137.9 (C), 170.1 (C), 172.5 (C), 208.8 (C); HRMS (EI) *m/z* calcd for $C_{17}H_{14}O_4$ [M+] 282.0892, found: 282.0892; mp = 205–208 °C.

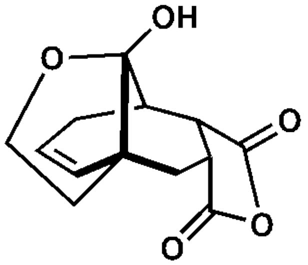

9-Hydroxy-5,10- dioxatetracyclo[6.4.3.0^{1,9}.0^{3,7}]pentadec-3-ene-4,6-dione (**32**)

Yield 56% (10 mg); IR (neat, cm^{-1}) ν_{max} 3493, 2927, 1852, 1774, 1716; ^{1}H NMR (400 MHz, $CDCl_3$) δ ppm 1.79 (dd, *J* = 11.9, 6.9 Hz, 1 H), 2.08 (m, 2 H), 2.23 (m, 2H), 2.45 (d, *J* = 13.7 Hz, 2 H), 2.66 (m, 1 H), 3.18 (m, 1 H), 3.60 (dd, *J* = 9.9, 8.3 Hz, 1H), 3.79 (ddd, *J* = 10.1, 8.0, 7.2 Hz, 1 H), 4.06 (m, 1 H), 5.22 (dd, *J* = 9.6, 2.9 Hz, 1 H), 5.65 (m, 1 H); ^{13}C NMR (101 MHz, $CDCl_3$) δ ppm 27.1 (CH_2), 29.4 (CH_2), 32.1 (CH_2), 37.4 (CH), 39.0 (CH), 45.4 (CH), 45.5 (C), 66.9 (CH_2), 102.7 (C), 126.8 (CH), 132.3 (CH), 172.9 (C), 173.9 (C); HRMS (EI) *m/z* calcd for $C_{13}H_{14}O_5$ [M+] 250.0841, found: 250.0831; mp=63-65˚C.

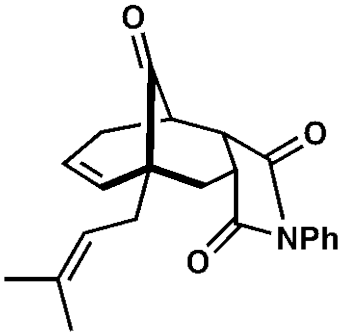

8-(2-Methyl-2-butene)-allyl-4-phenyl-4-azatricyclo[6.3.1.0^{2,6}]dodec-9-ene-3,5,12-trione (**28**)

Yield: 81% (17 mg); IR (neat, cm^{-1}) ν_{max} 2967, 2941, 1713, 1529; ^1H NMR (300 MHz, $CDCl_3$) δ 7.56 - 7.36 (m, 3 H), 7.30 - 7.25 (m, 2 H), 5.85 - 5.75 (m, 1 H), 5.54 (dd, *J* = 1.6, 9.4 Hz, 1 H), 5.14 - 5.04 (m, 1 H), 3.69 (t, *J* = 9.0 Hz, 1 H), 3.14 - 3.00 (m, 2 H), 2.93 (d, *J* = 14.4 Hz, 1 H), 2.70 - 2.52 (m, 2 H), 2.38 (dd, *J* = 7.6, 14.8 Hz, 1 H), 2.19 (dd, *J* = 7.5, 14.7 Hz, 1 H), 2.04 (dd, *J* = 7.9, 14.4 Hz, 1 H), 1.71 (d, *J* = 0.9 Hz, 3 H), 1.63 (s, 3 H). ^{13}C NMR (75 MHz, $CDCl_3$) δ 212.8, 177.6, 175.9, 135.0, 134.2, 131.9, 129.4, 128.9, 126.4, 126.2, 118.6, 49.9, 47.7, 44.9, 37.7, 37.0, 32.5, 32.4, 26.2, 18.1; HRMS (ESI) *m/z* calcd for $C_{22}H_{23}NO_3$ [M+] 349.1678, found: 349.1678.

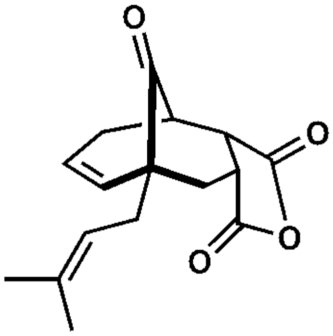

8-(2-Methylbutenyl)-4-oxatricyclo[6.3.1.$0^{2,6}$]dodec-9-ene-3,5,12-trione (**29**)

Yield: 78% (67 mg); IR (neat, cm^{-1}) ν_{max} 2946, 2869, 1751, 1731, 1715, 1464; ^1H NMR (500 MHz, $CDCl_3$) δ 6.27 (ddd, *J* = 1.2, 7.3, 8.4 Hz, 1 H), 6.20 (dd, *J* = 7.7, 7.9 Hz, 1 H), 4.98 (dd, *J* = 1.4, 7.2 Hz, 1 H), 3.48 (dd, *J* = 5.8, 7.0 Hz, 1 H), 3.34 (dd, *J* = 3.7, 5.5 Hz, 1 H), 3.21 (dd, *J* = 3.7, 5.7 Hz, 1 H), 2.86 (d, *J* = 7.3 Hz, 1 H), 2.54 (dd, *J* = 6.2, 14.9 Hz, 1 H), 2.26 (dd, *J* = 8.0, 14.8 Hz, 1 H), 2.04 (dd, *J* = 4.4, 13.1 Hz, 1 H), 1.85 (d, *J* = 13.1 Hz, 1 H), 1.67 (s, 3 H), 1.63 (s, 3 H). ^{13}C NMR (75 MHz, $CDCl_3$) □= 209.9, 169.6, 135.6, 133.1, 131.5, 118.5, 55.9, 55.2, 51.6, 51.4, 49.4, 38.0, 30.7, 26.1, 17.9, 17.8. HRMS (EI) *m/z* calcd for $C_{15}H_{18}O_3$[M+ (-CO)] 246.1256, found: 246.1378.

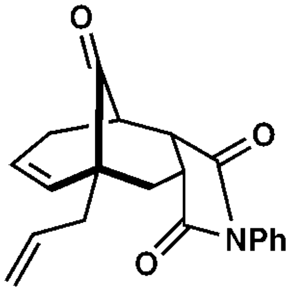

8-Allyl-4-phenyl-4-azatricyclo[6.3.1.0^{2,6}]dodec-9-ene-3,5,12-trione (**18**)

Yield: 80% (211 mg); IR (neat, cm^{-1}) ν_{max} 3078, 2921, 1712; ^{1}H NMR (300 MHz, $CDCl_3$) δ 7.54 - 7.35 (m, 3 H), 7.30 - 7.22 (m, 2 H), 5.87 - 5.64 (m, 2 H), 5.53 (d, *J* = 9.8 Hz, 1 H), 5.11 (d, *J* = 3.9 Hz, 1 H), 5.06 (s, 1 H), 3.66 (t, *J* = 8.9 Hz, 1 H), 3.14 - 2.97 (m, 2 H), 2.87 (d, *J* = 14.3 Hz, 1 H), 2.59 (br. s., 2 H), 2.47 (dd, *J* = 6.9, 14.0 Hz, 1 H), 2.20 (dd, *J* = 7.6, 14.0 Hz, 1 H), 2.03 (dd, *J* = 7.8, 14.4 Hz, 1 H). ^{13}C NMR (75 MHz, $CDCl_3$) δ 212.1, 177.5, 175.8, 133.8, 133.2, 131.8, 129.4, 128.8, 126.6, 126.2, 118.7, 49.1, 47.6, 44.8, 38.5, 37.6, 37.0, 32.3; HRMS (EI) *m/z* calcd $C_{20}H_{19}NO_3$ [M+] 321.1365, found: 321.1364.

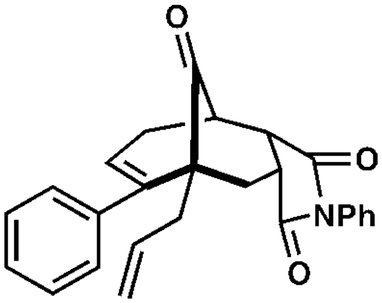

4,9-Diphenyl-8-allyl-4-azatricyclo[6.3.1.0^{2,6}]dodec-9-ene-3,5,12-trione (**37**)

Yield: 68% (178 mg); IR (neat, cm^{-1}) ν_{max} 3072, 2931, 1777, 1713, 1636, 1500, 1445. ^{1}H NMR (500 MHz, $CDCl_3$) δ 7.49 - 7.40 (m, 4 H), 7.40 - 7.34 (m, 1 H), 7.34 - 7.27 (m, 3 H), 7.23 - 7.16 (m, 2 H), 5.86 (dd, *J* = 2.7, 4.4 Hz, 1 H), 5.56 (tdd, *J* = 6.8, 10.3, 17.1 Hz, 1 H), 4.85 (d, *J* = 10.3 Hz, 1 H), 4.63 (dd, *J* = 2.0, 17.1 Hz, 1 H), 3.76 (t, *J* = 9.0 Hz, 1 H), 3.39 (d, *J* = 14.9 Hz, 1 H), 3.28 - 3.13 (m, 2 H), 2.79 - 2.65 (m, 2 H), 2.30 - 2.12 (m, 3 H). ^{13}C NMR (101MHz, $CDCl_3$) δ 211.4, 176.7, 175.8, 143.8, 139.9, 133.6, 131.8, 129.4, 129.1, 128.9, 128.1, 127.8, 127.6, 126.3, 118.4, 51.2, 47.6,

44.0, 37.6, 37.6, 36.5, 31.5; HRMS *m/z* calcd for $C_{26}H_{23}NO_3$ [M+] 397.1678, found: 397.1685.

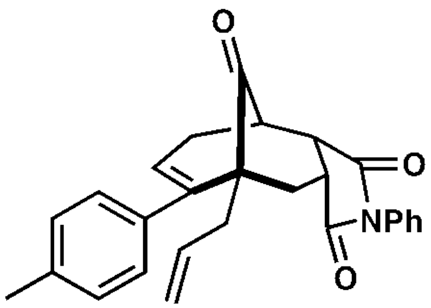

8-Allyl-9-(4-methylphenyl)-4-phenyl-4-azatricyclo[6.3.1.0^{2,6}]dodec-9-ene-3,5,12-trione (**38**)

Yield: 91% (237 mg); IR (neat, cm^{-1}) ν_{max} 3072, 2924, 1714, 1593, 1500. ^{1}H NMR (500 MHz, $CDCl_3$) δ 7.47 - 7.39 (m, 2 H), 7.39 - 7.29 (m, 3 H), 7.19 (d, *J* = 7.6 Hz, 2H), 7.11 (d, *J* = 7.8 Hz, 2 H), 5.84 (t, *J* = 3.9 Hz, 1 H), 5.56 (ddt, *J* = 7.1, 10.3, 16.9 Hz, 1 H), 4.86 (dd, *J* = 1.2, 10.3 Hz, 1 H), 4.67 (dd, *J* = 1.2, 17.1 Hz, 1 H), 3.74 (t, *J* = 9.0 Hz, 1 H), 3.37 (d, *J* = 15.1 Hz, 1 H), 3.25 - 3.12 (m, 2 H), 2.78 - 2.63 (m, 2H), 2.34 (s, 3 H), 2.30 - 2.24 (m, 2 H), 2.20 (dd, *J* = 7.8, 14.9 Hz, 1 H); ^{13}C NMR (126 MHz, $CDCl_3$) δ 211.5, 176.6, 175.9, 143.6, 137.4, 137.0, 133.7, 131.7, 129.4, 128.9, 128.8, 128.7, 127.2, 126.2, 118.3, 51.3, 47.5, 44.0, 37.6, 37.5, 36.4, 31.4, 21.3; HRMS (EI) m/z calcd for $C_{27}H_{25}NO_3$ [M+] 411.1834, found: 411.1807.

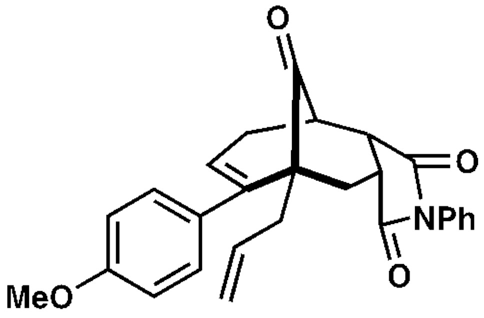

8-Allyl-9-(4-methoxyphenyl)-4-phenyl-4-azatricyclo[6.3.1.0^{2,6}]dodec-9-ene-3,5,12-trione (**39**)

Yield: 74% (192 mg); IR (neat, cm^{-1}) ν_{max} 2939, 2254, 1780, 1714, 1605, 1512; ^{1}H NMR (400 MHz, $CDCl_3$) δ 7.46 - 7.40 (m, 2 H), 7.39 - 7.33 (m, 3 H), 7.24 - 7.15

(m, 2 H), 6.84 (d, *J* = 8.8 Hz, 2 H), 5.83 (t, *J* = 3.2 Hz, 1 H), 5.55 (ddt, *J* = 6.9, 10.3, 17.1 Hz, 1 H), 4.85 (dd, *J* = 2.0, 10.2 Hz, 1 H), 4.66 (dd, *J* = 2.0, 17.1 Hz, 1 H), 3.80 (s, 3 H), 3.75 (t, *J* = 8.8 Hz, 1 H), 3.36 (d, *J* = 14.9 Hz, 1 H), 3.26 - 3.13 (m, 2 H), 2.78 - 2.59 (m, 2 H), 2.34 - 2.14 (m, 3 H). ^{13}C NMR (101 MHz, $CDCl_3$) δ 211.5, 176.7, 175.9, 159.2, 143.4, 133.7, 132.5, 131.8, 130.2, 129.4, 128.8, 127.2, 126.3, 118.3, 113.4, 55.3, 51.4, 47.6, 44.1, 37.7, 37.5, 36.5, 31.4; HRMS (EI) *m/z* calcd for $C_{27}H_{25}NO_4$ [M+] 427.1784, found: 427.1795.

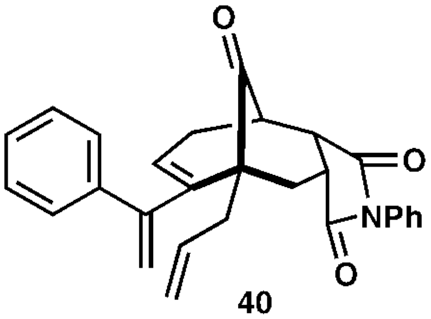

40

8-Allyl-9-(1-phenylethenyl)-4-phenyl-4-azatricyclo[6.3.1.0^{2,6}]dodec-9-ene-3,5,12-trione (**37**)

Yield: 79% (259 mg); IR (neat, cm^{-1}) ν_{max} 2957, 2922, 2855, 1712; ^{1}H NMR (400 MHz, $CDCl_3$) δ 7.50 - 7.38 (m, 4 H), 7.33 - 7.27 (m, 4 H), 7.25 - 7.22 (m, 2 H), 5.78 (tdd, *J* = 7.4, 10.5, 18.4 Hz, 1 H), 5.70 (dd, *J* = 2.1, 4.9 Hz, 1 H), 5.68 (s, 1 H), 5.53 (s, 1 H), 5.02 - 4.92 (m, 2 H), 3.70 (t, *J* = 8.8 Hz, 1 H), 3.24 - 3.12 (m, 2 H), 2.76 (ddd, *J* = 2.3, 5.9, 19.5 Hz, 1 H), 2.59 (ddd, *J* = 1.2, 4.7, 19.1 Hz, 1 H), 2.48 (dd, *J* = 6.6, 14.1 Hz, 1 H), 2.24 (dt, *J* = 8.2, 15.2 Hz, 3 H); ^{13}C NMR (101 MHz, $CDCl_3$) δ 211.5, 176.6, 175.9, 146.5, 142.7, 141.9, 134.2, 131.8, 129.5, 129.3, 129.0, 128.4, 127.8, 127.5, 126.4, 118.8, 117.0, 51.1, 47.4, 43.9, 37.3, 37.0, 34.6, 31.8; HRMS (EI) *m/z* calcd for $C_{28}H_{25}NO_3$ [M+] 423.1834, found: 423.1840.

References

1. Ahmad, N. M.; Rodeschini, V.; Simpkins, N. S.; Ward, S. E.; Blake, A. J. *J. Org Chem.* **2007,** *72,* 4803. doi:10.1021/jo070388h
2. Savignac, P.; Bréque, A. *Synth. Comm.* **1979,** *9,* 487. doi:10.1080/00397917908060952

^{1}H NMR (400 MHz, $CDCl_3$)

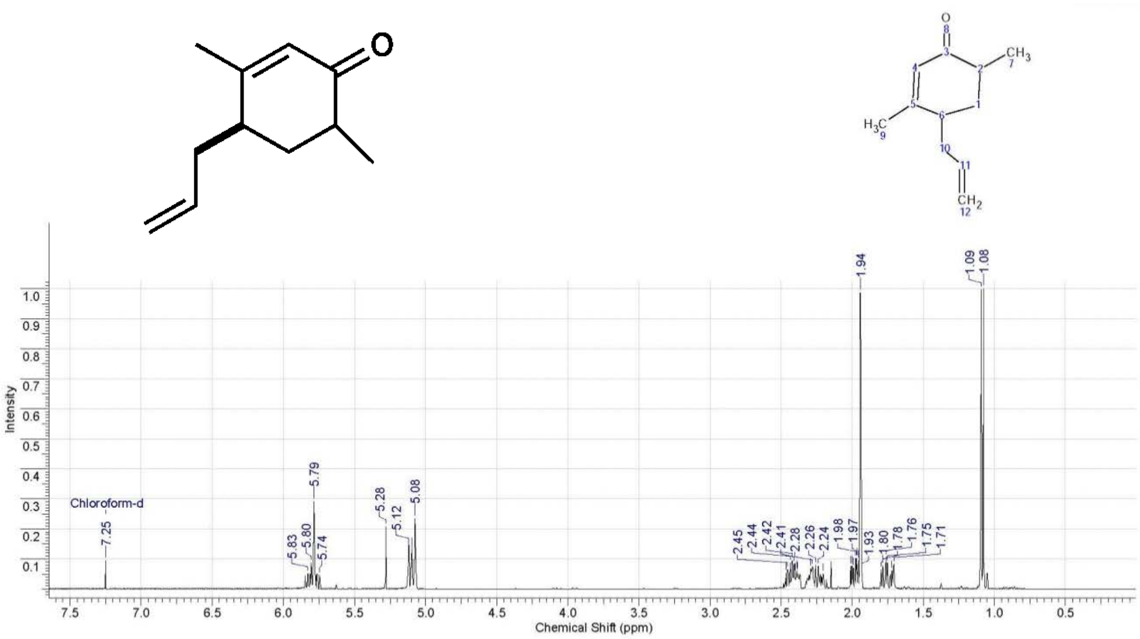

^{13}C NMR (101 MHz, $CDCl_3$)

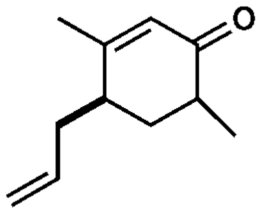

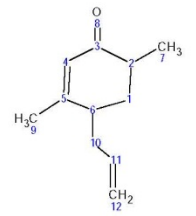

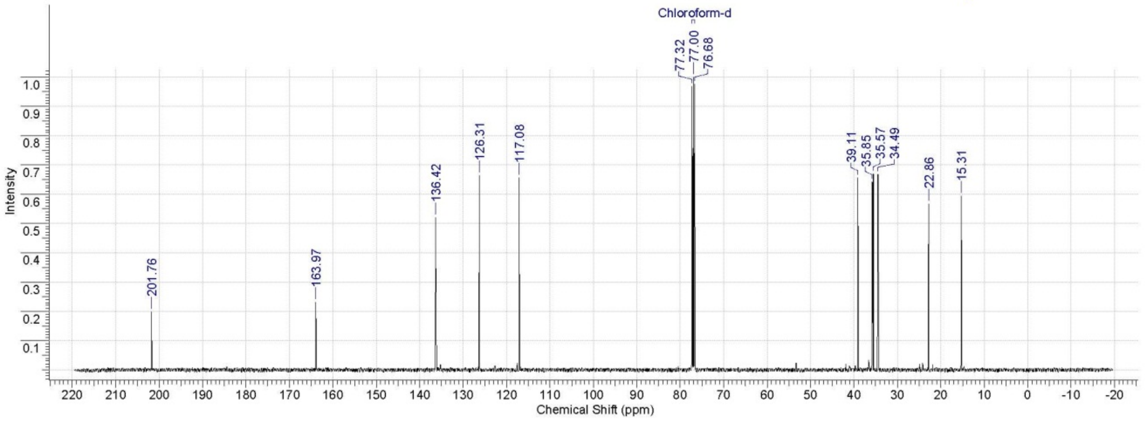

^{1}H NMR (400 MHz, $CDCl_3$)

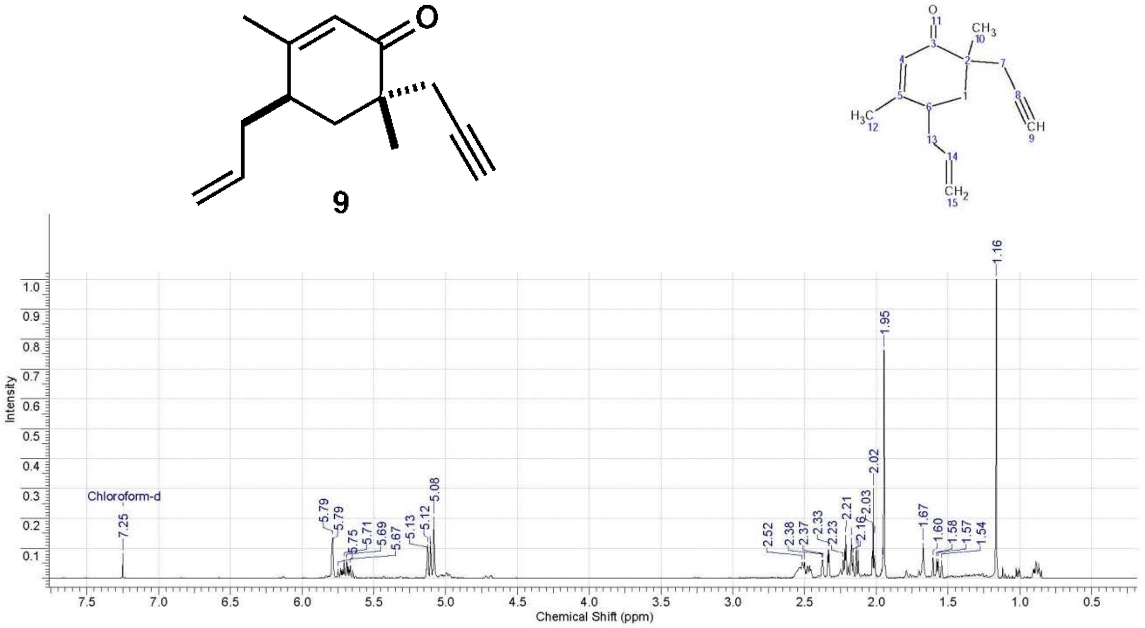

^{13}C NMR (101 MHz, $CDCl_3$)

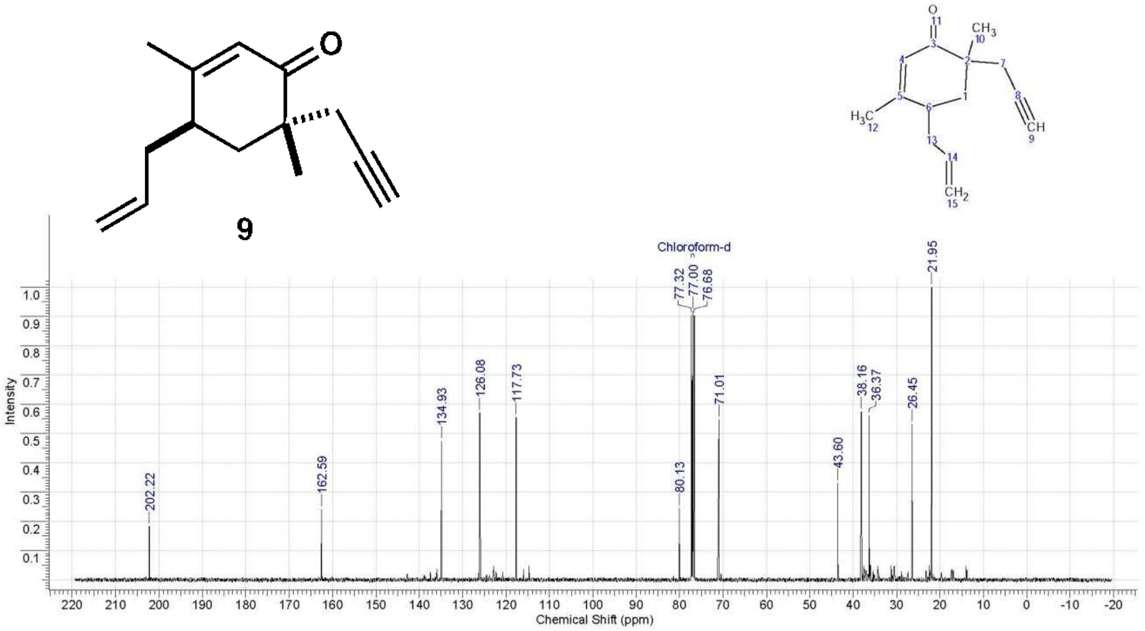

^{1}H NMR (400 MHz, $CDCl_3$)

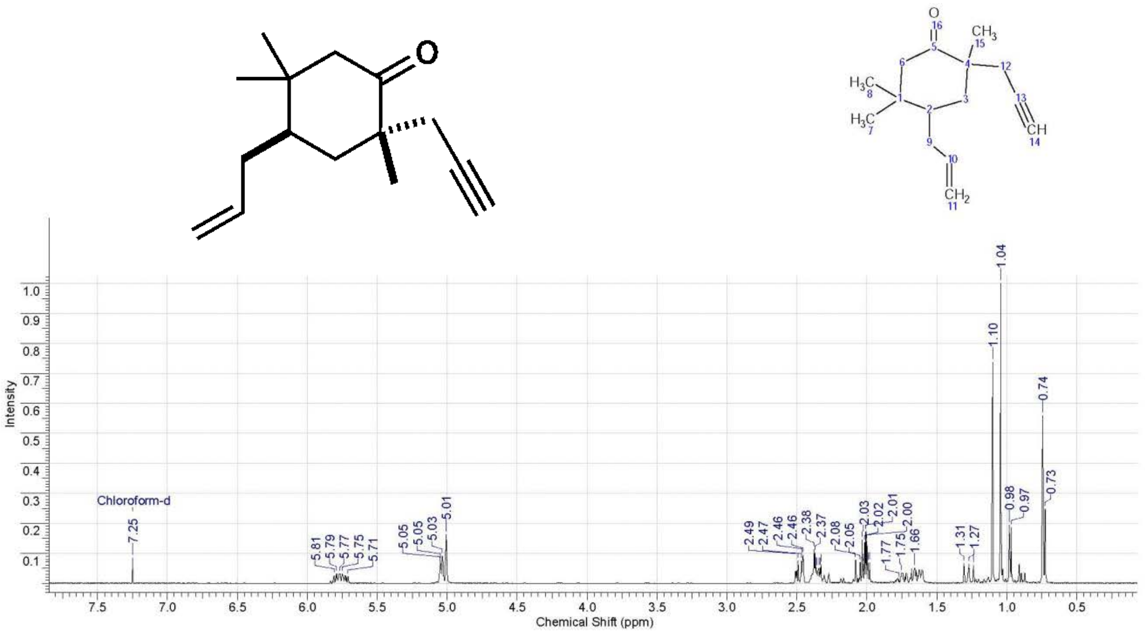

^{13}C NMR (101 MHz, $CDCl_3$)

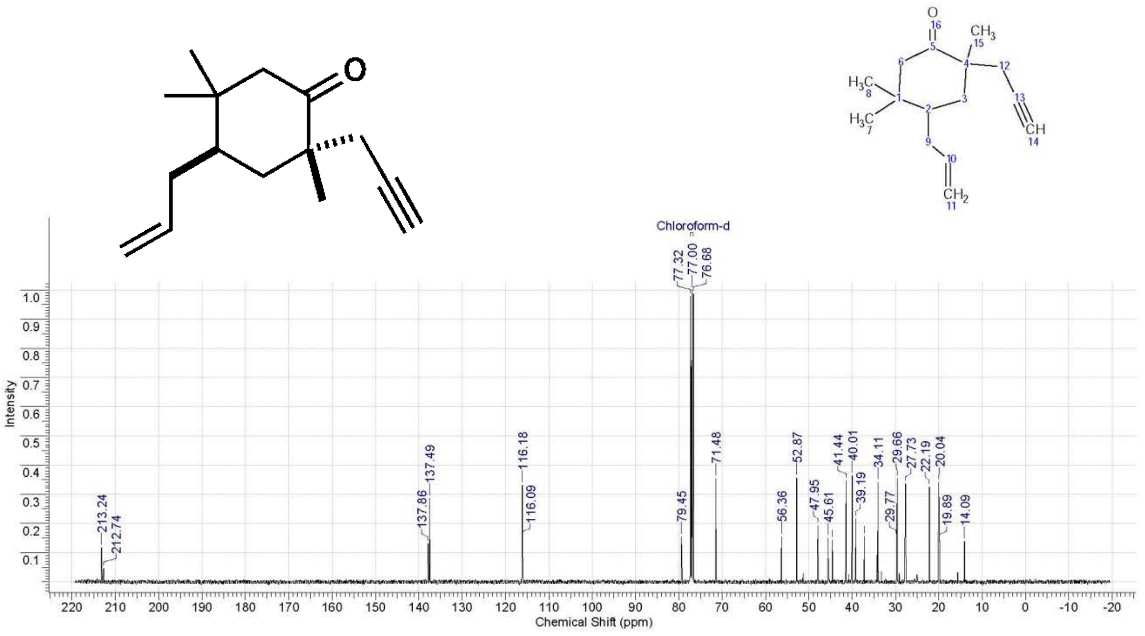

^{1}H NMR (400 MHz, $CDCl_3$)

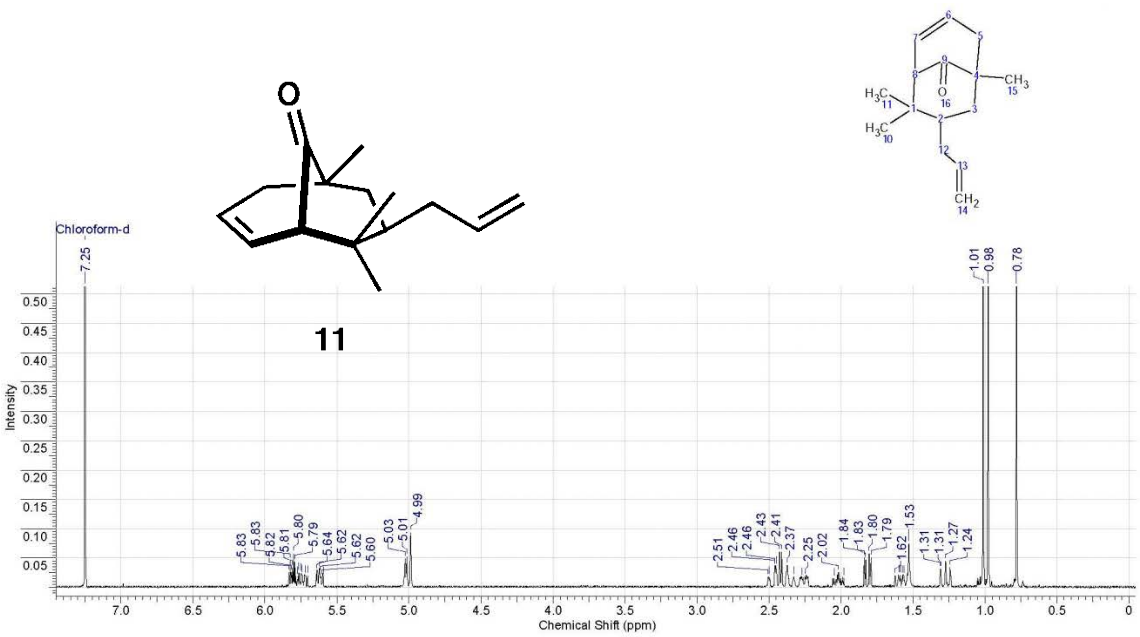

^{13}C NMR (101 MHz, $CDCl_3$)

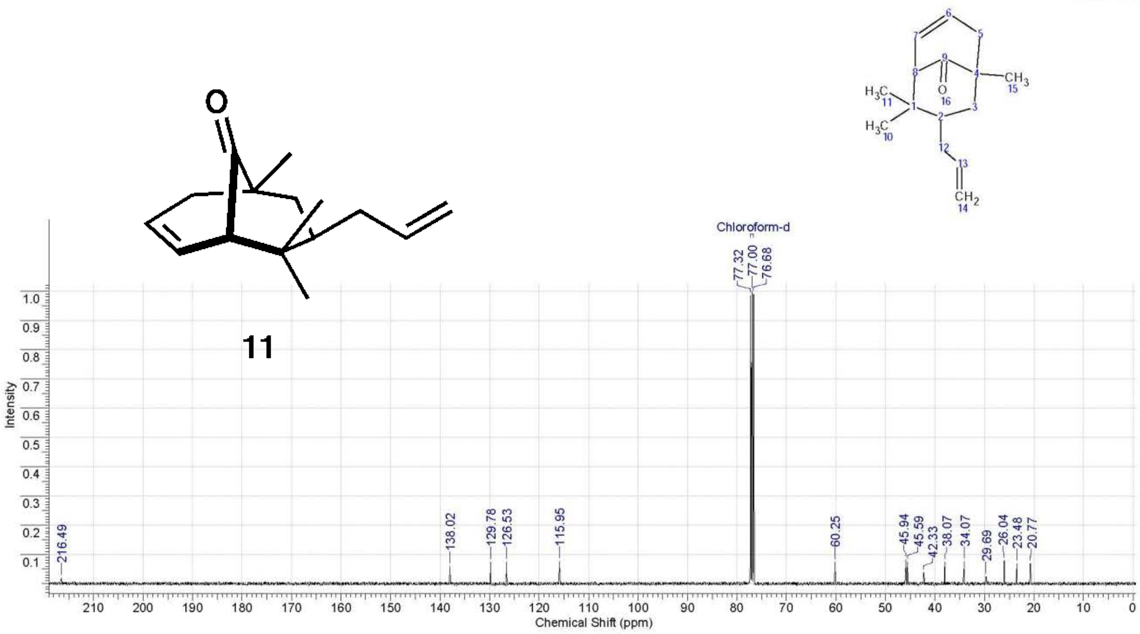

^{1}H NMR (400 MHz, $CDCl_3$)

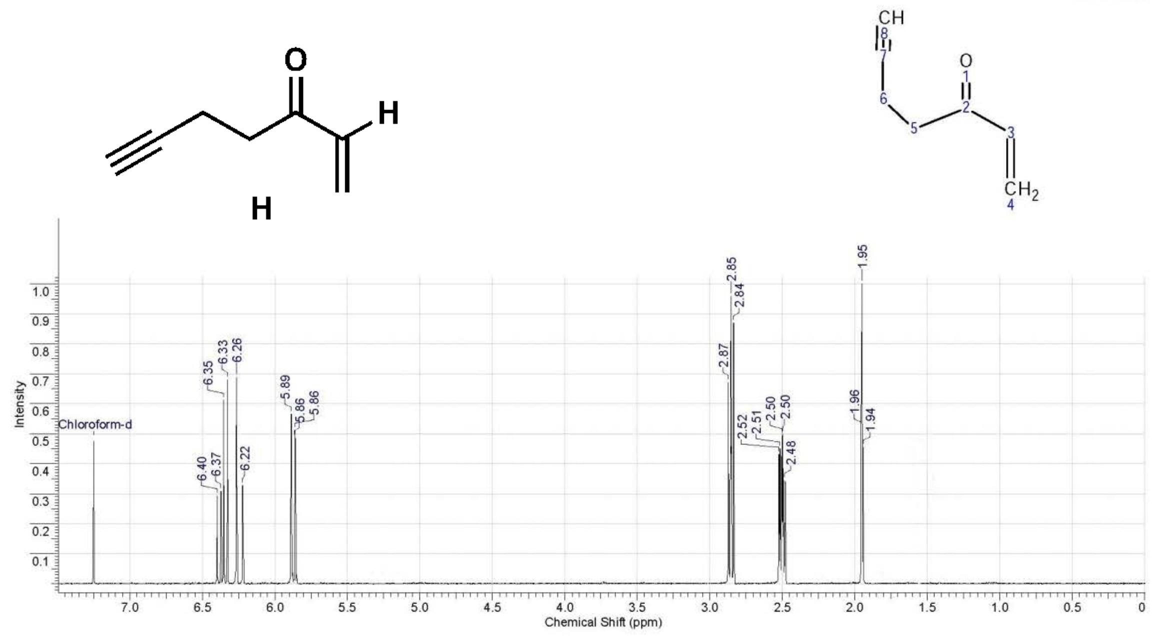

^{13}C NMR (101 MHz, $CDCl_3$)

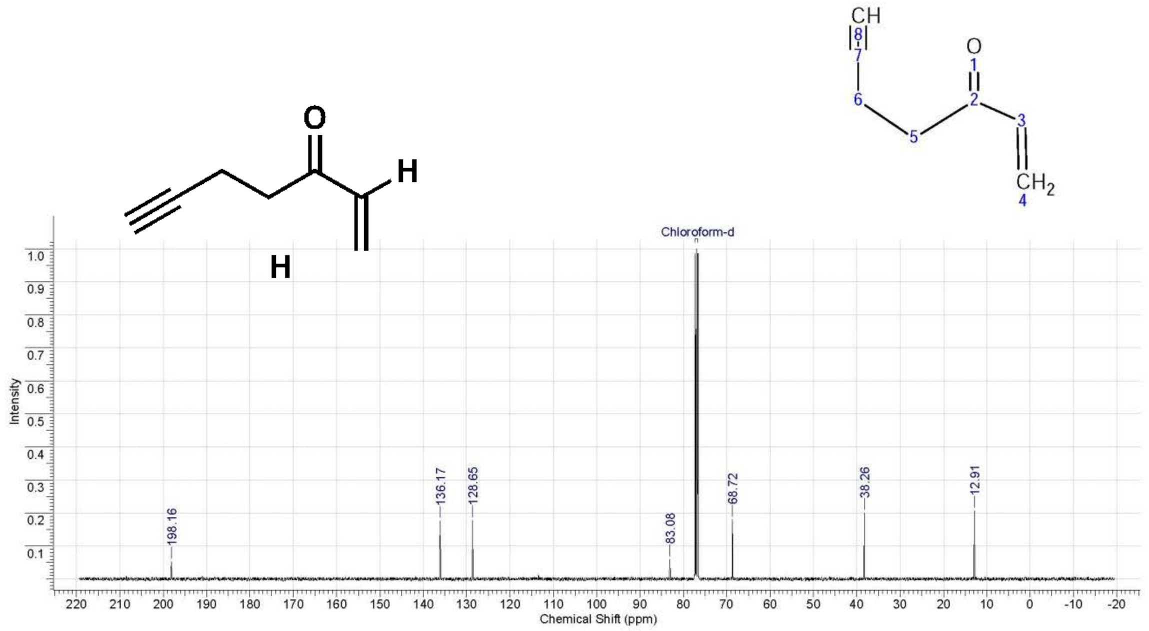

^{1}H NMR (400 MHz, $CDCl_3$)

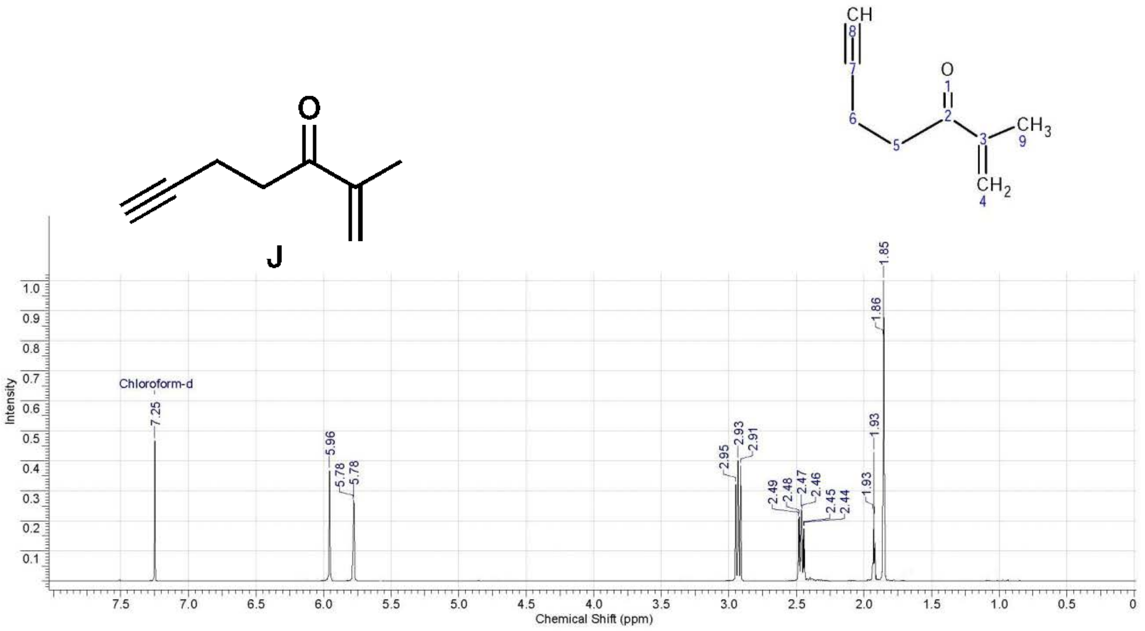

^{13}C NMR (101 MHz, $CDCl_3$)

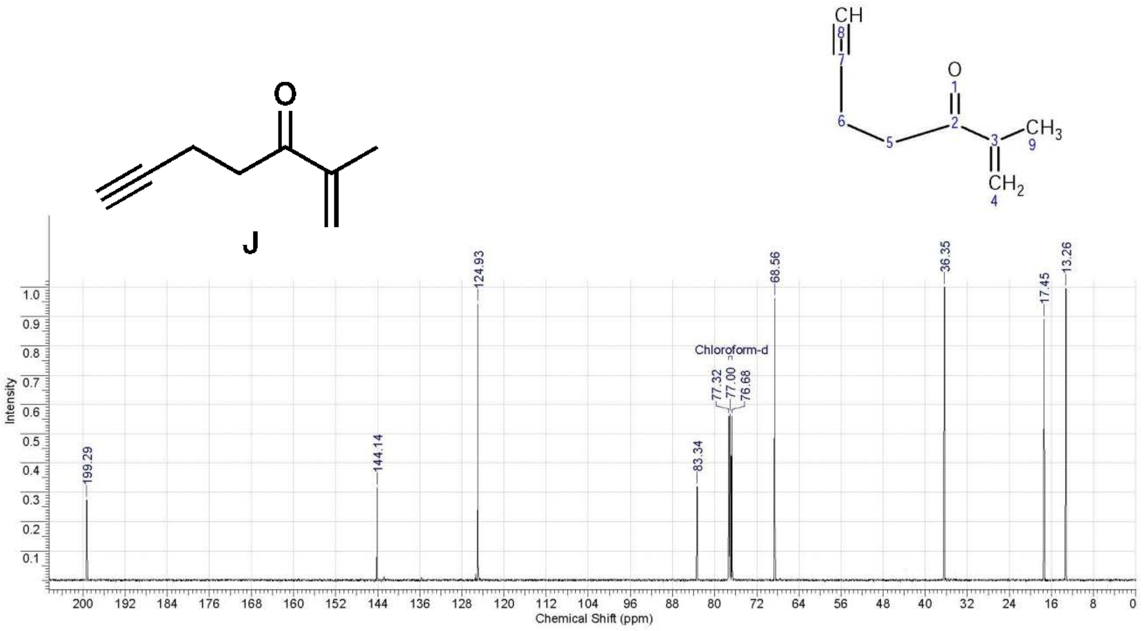

^{1}H NMR (400 MHz, $CDCl_3$)

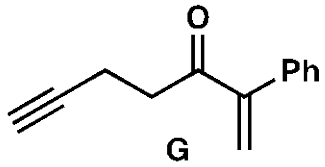

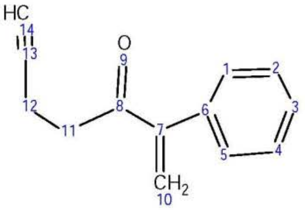

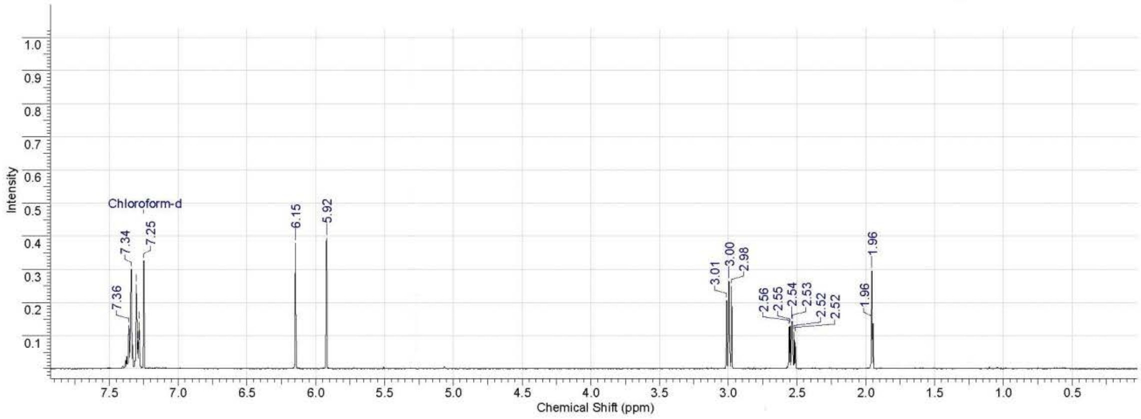

^{13}C NMR (101 MHz, $CDCl_3$)

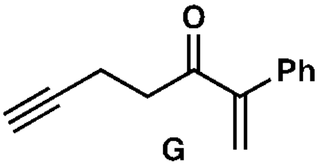

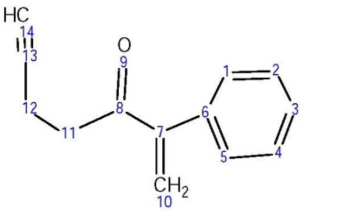

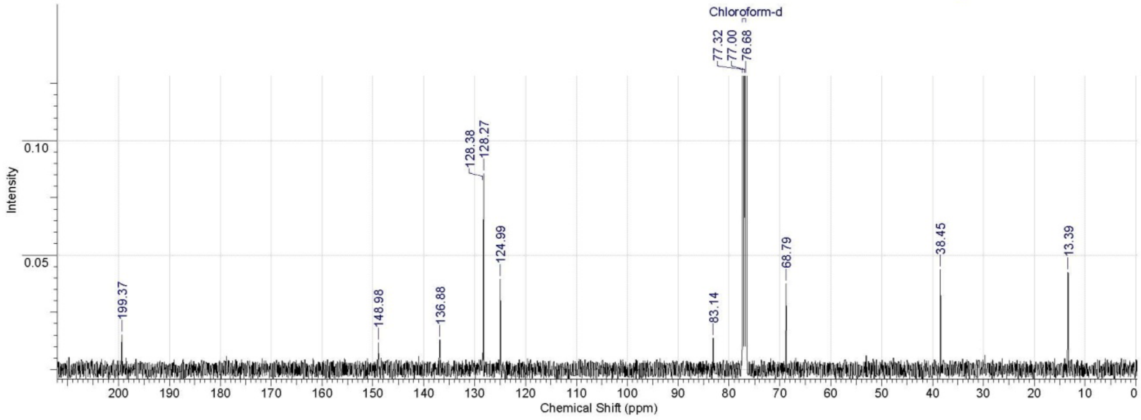

^{1}H NMR (400 MHz, $CDCl_3$)

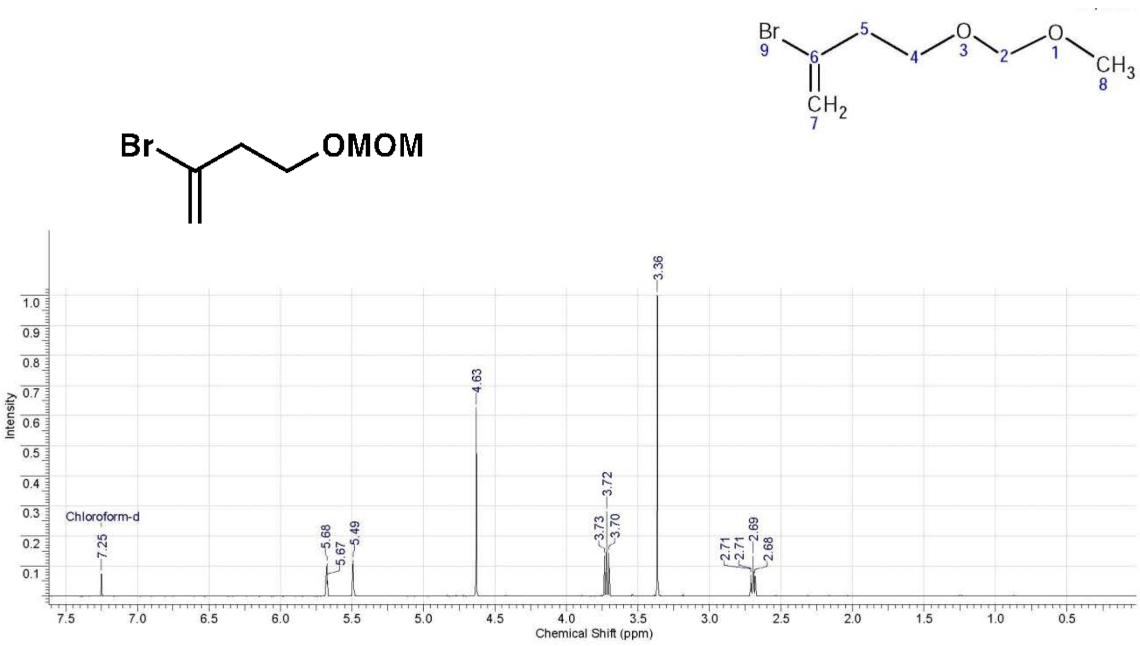

^{13}C NMR (101 MHz, $CDCl_3$)

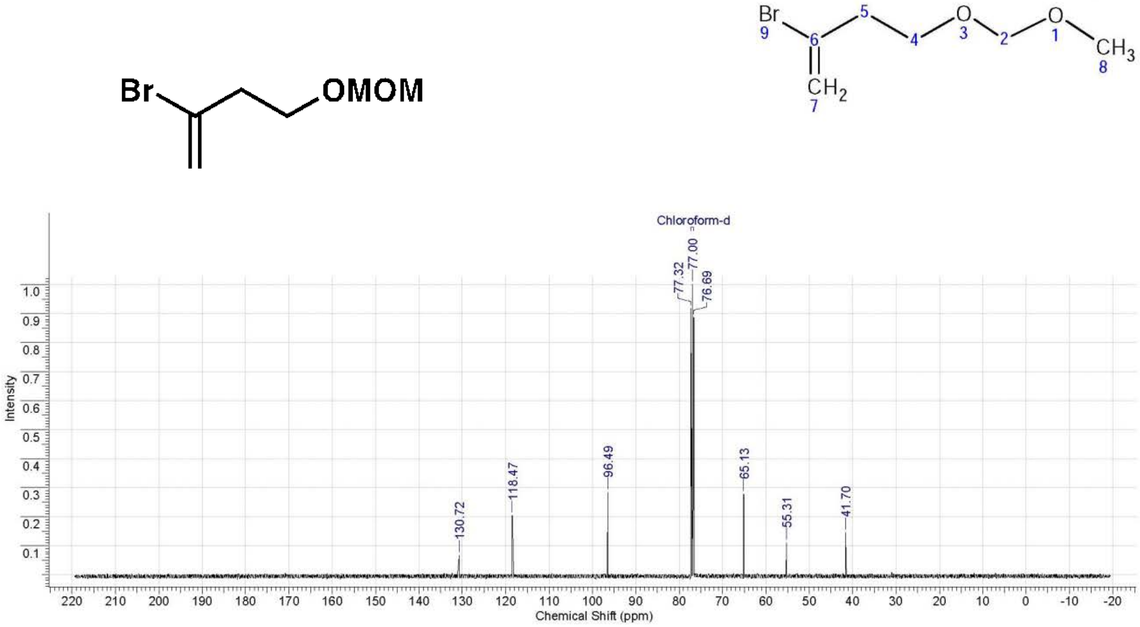

^{1}H NMR (400 MHz, $CDCl_3$)

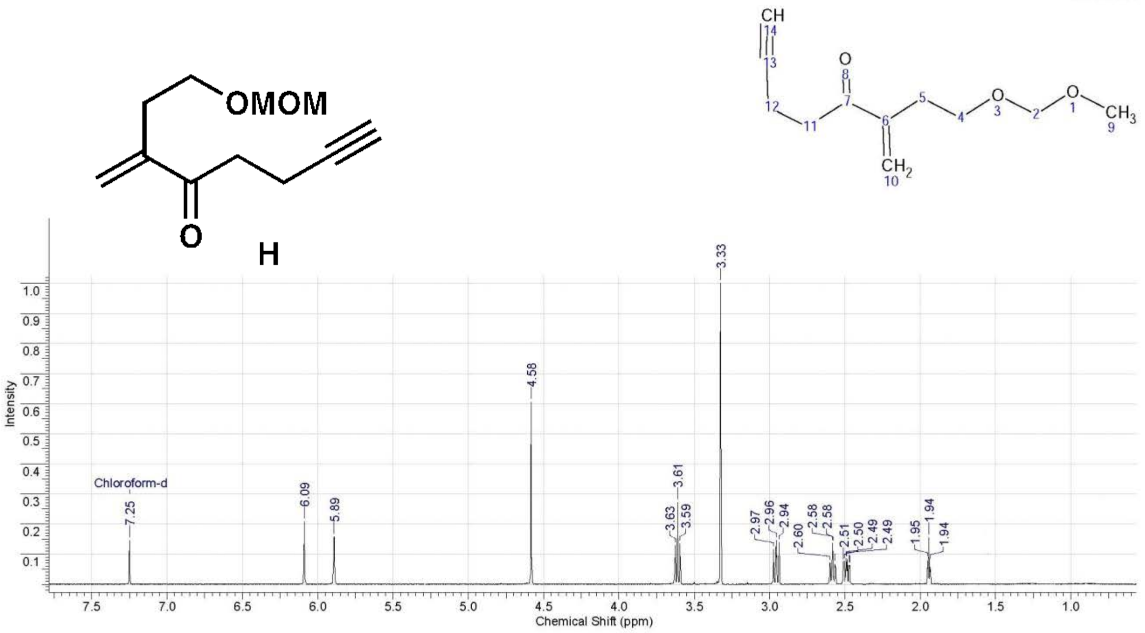

^{13}C NMR (101 MHz, $CDCl_3$)

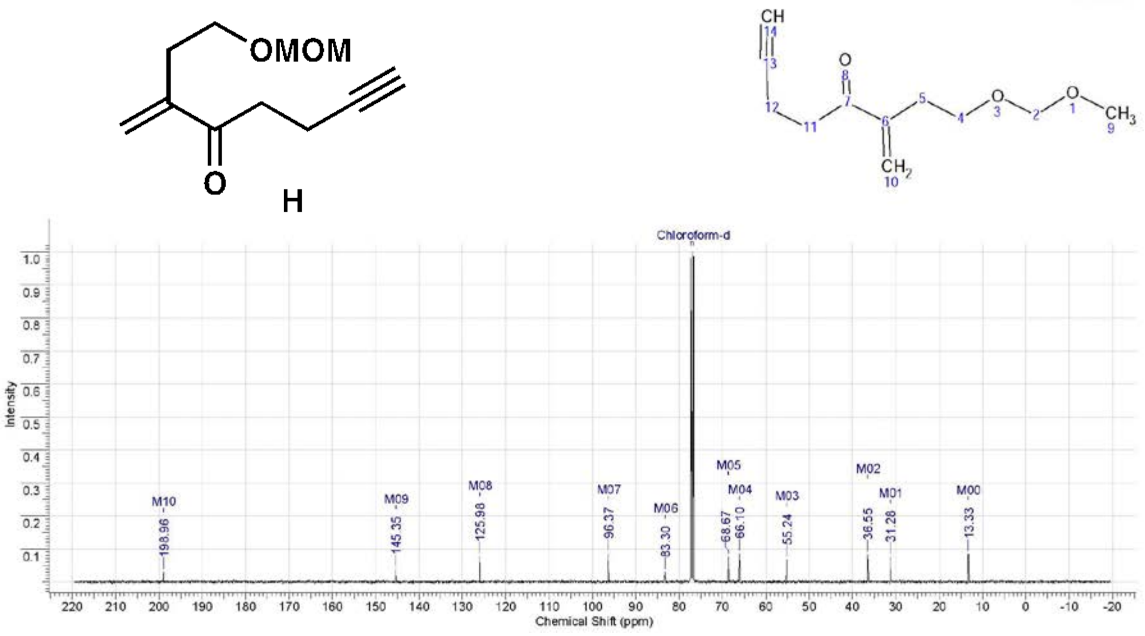

^{1}H NMR (400 MHz, $CDCl_3$)

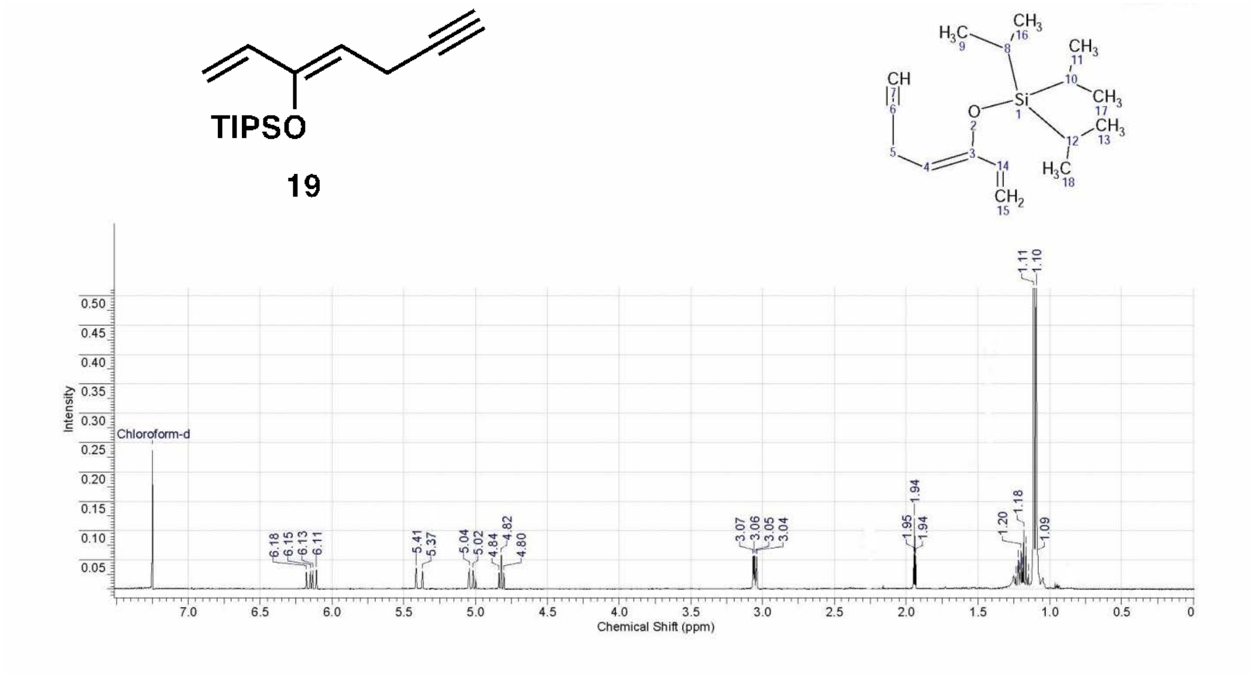

^{13}C NMR (101 MHz, $CDCl_3$)

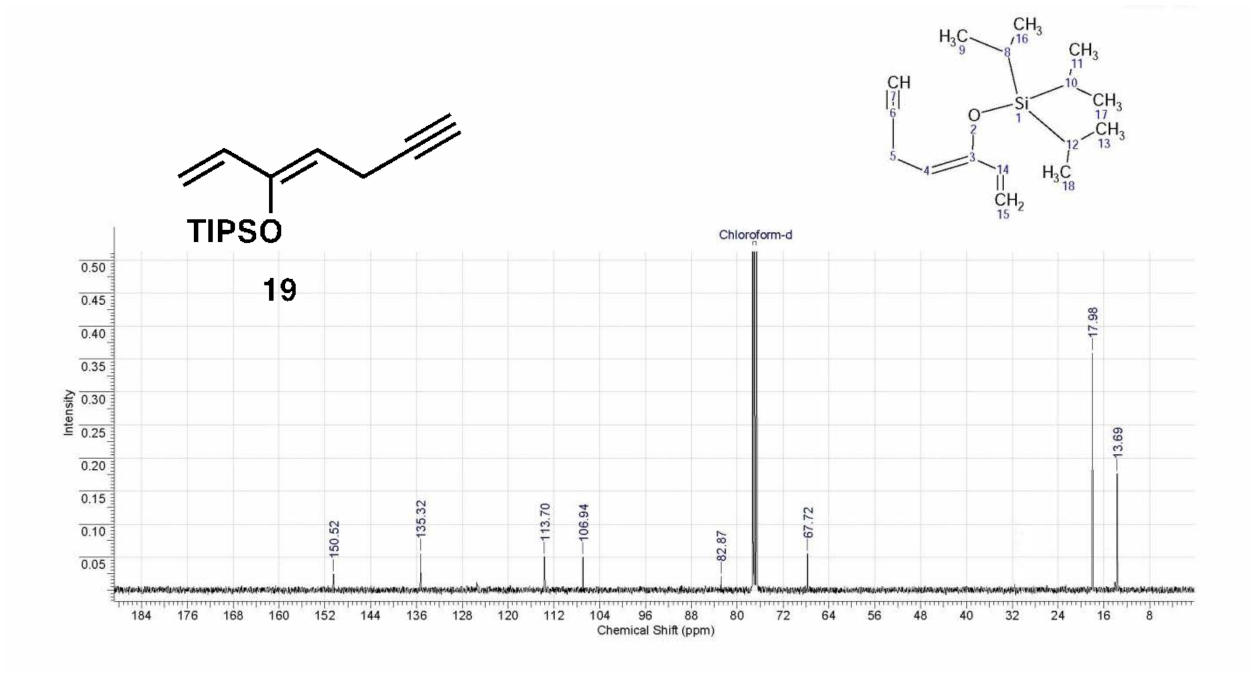

^{1}H NMR (400 MHz, $CDCl_3$)

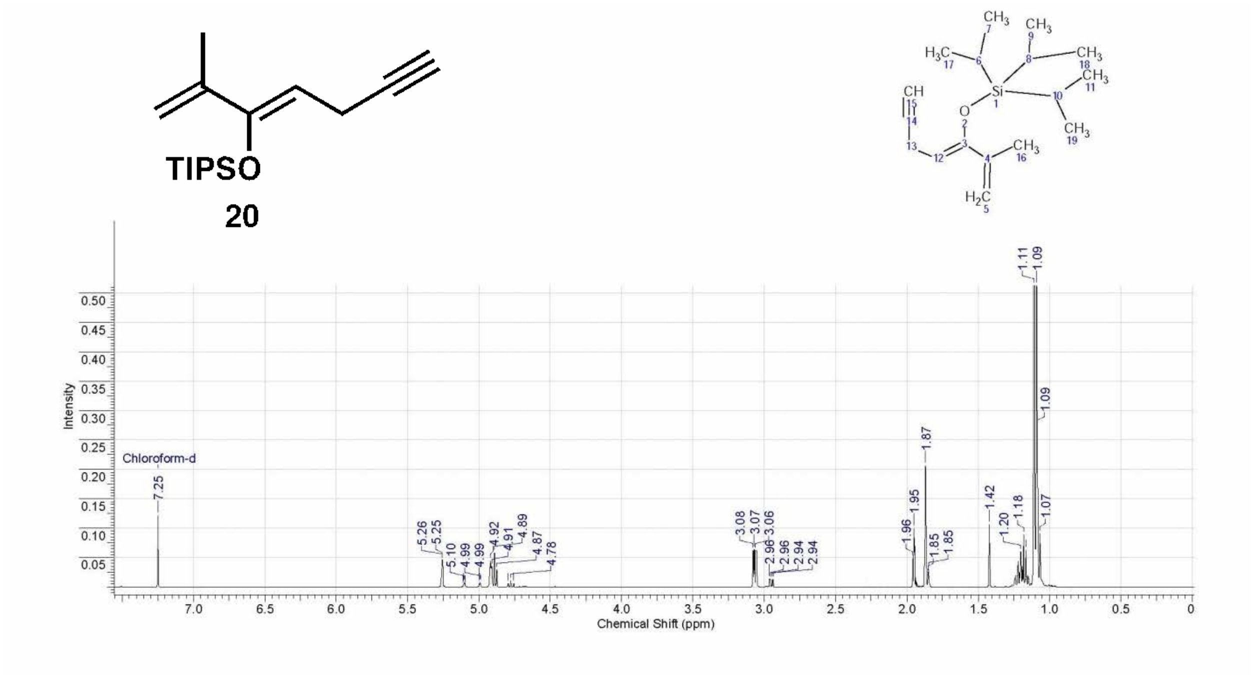

^{13}C NMR (101 MHz, $CDCl_3$)

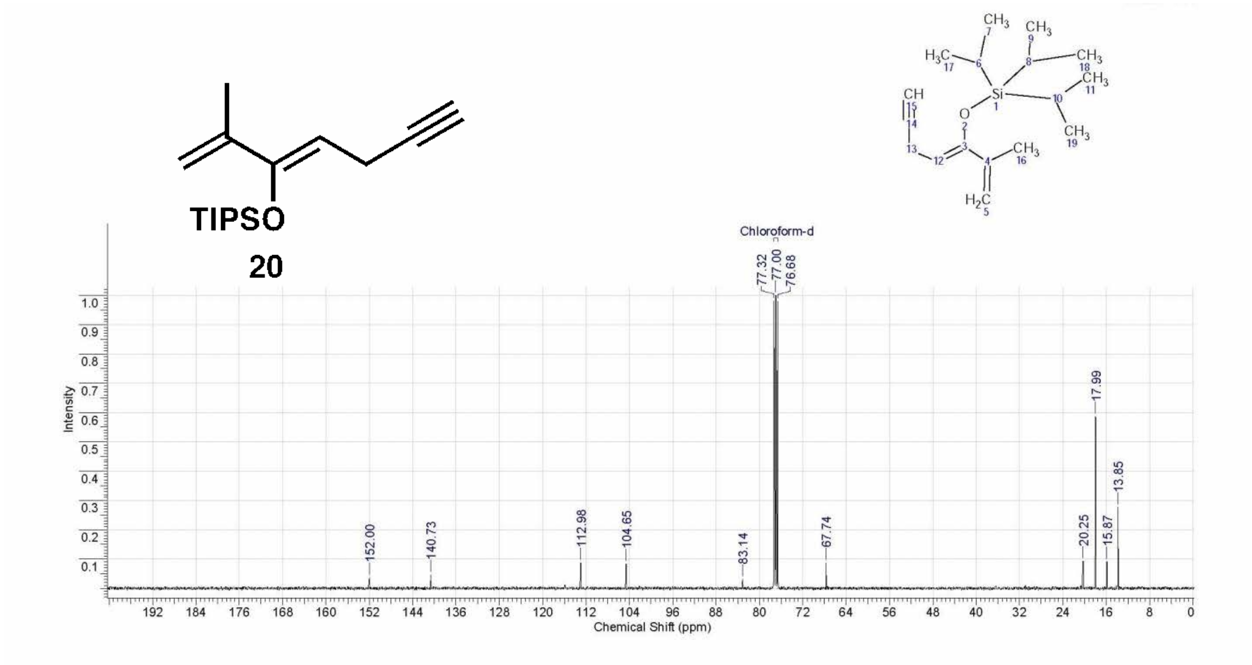

^{1}H NMR (400 MHz, $CDCl_3$)

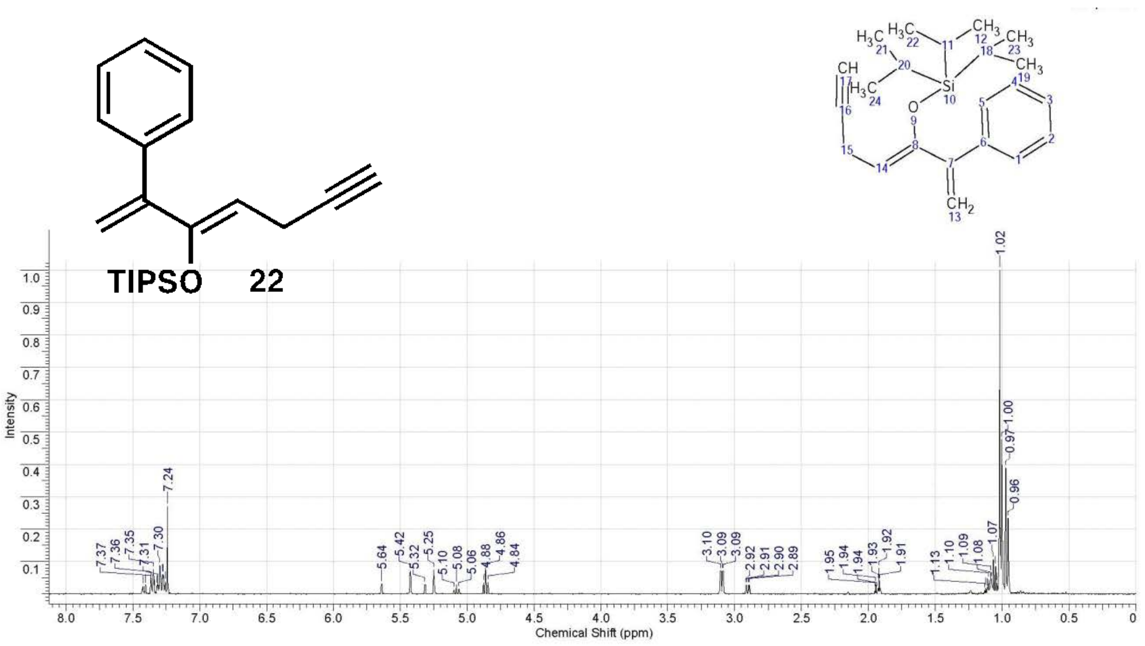

^{13}C NMR (101 MHz, $CDCl_3$)

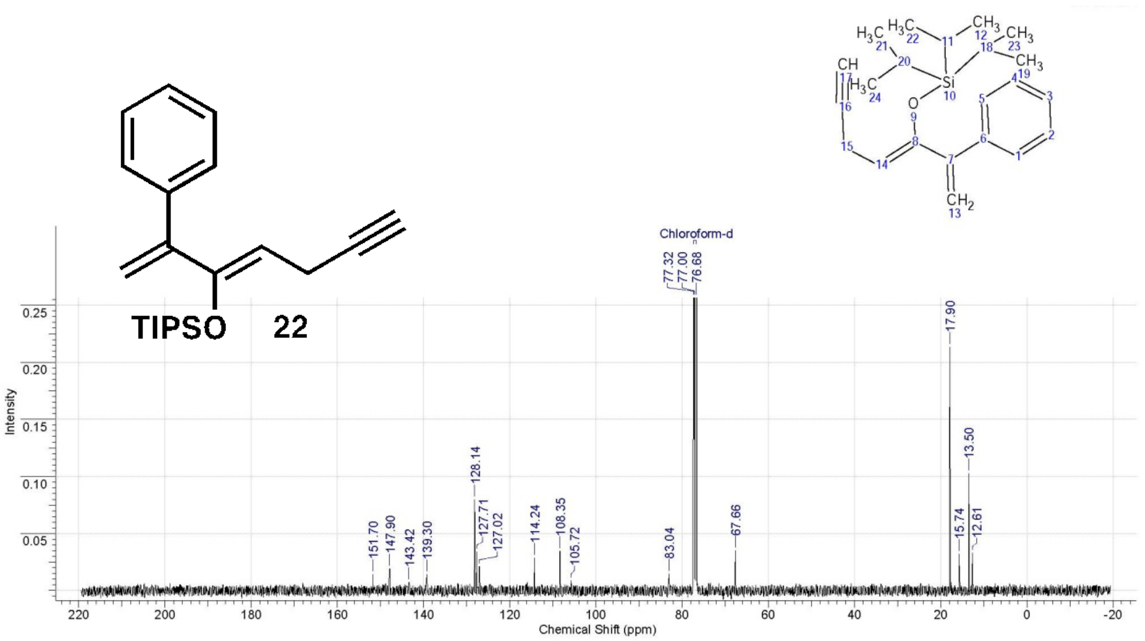

^{1}H NMR (400 MHz, $CDCl_3$)

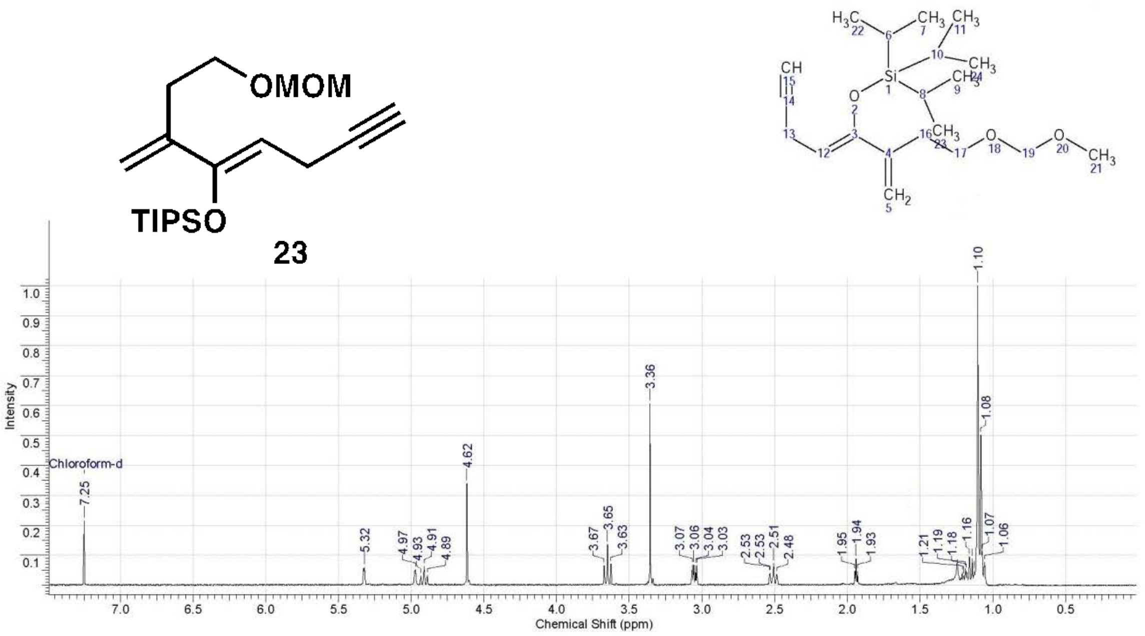

^{13}C NMR (101 MHz, $CDCl_3$)

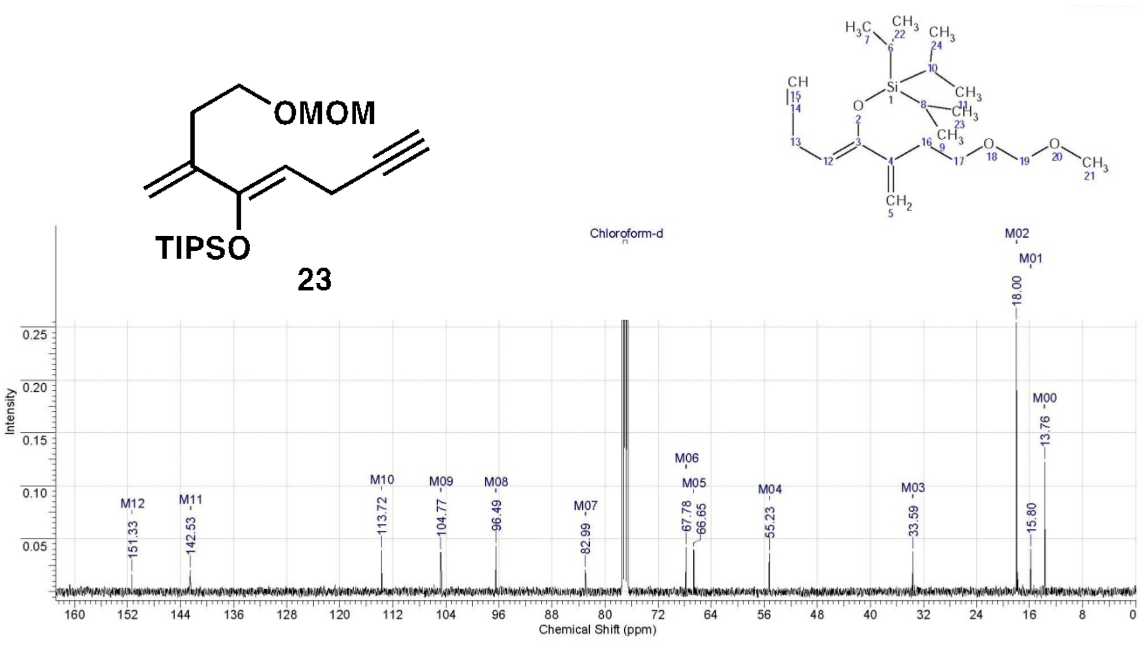

^{1}H NMR (400 MHz, $CDCl_3$)

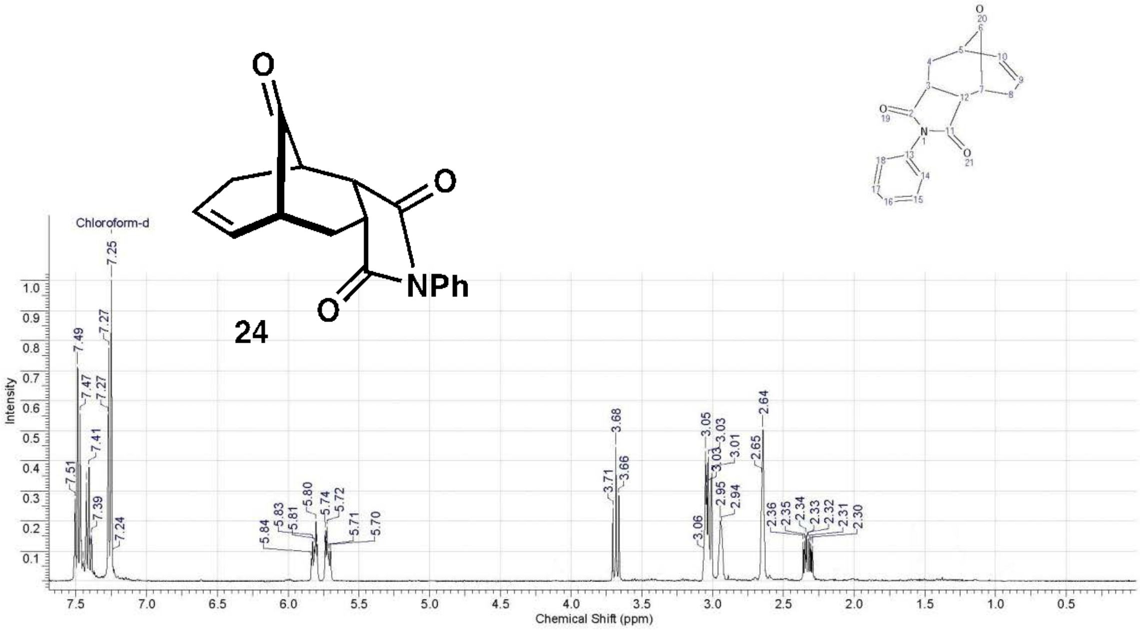

^{13}C NMR (101 MHz, $CDCl_3$)

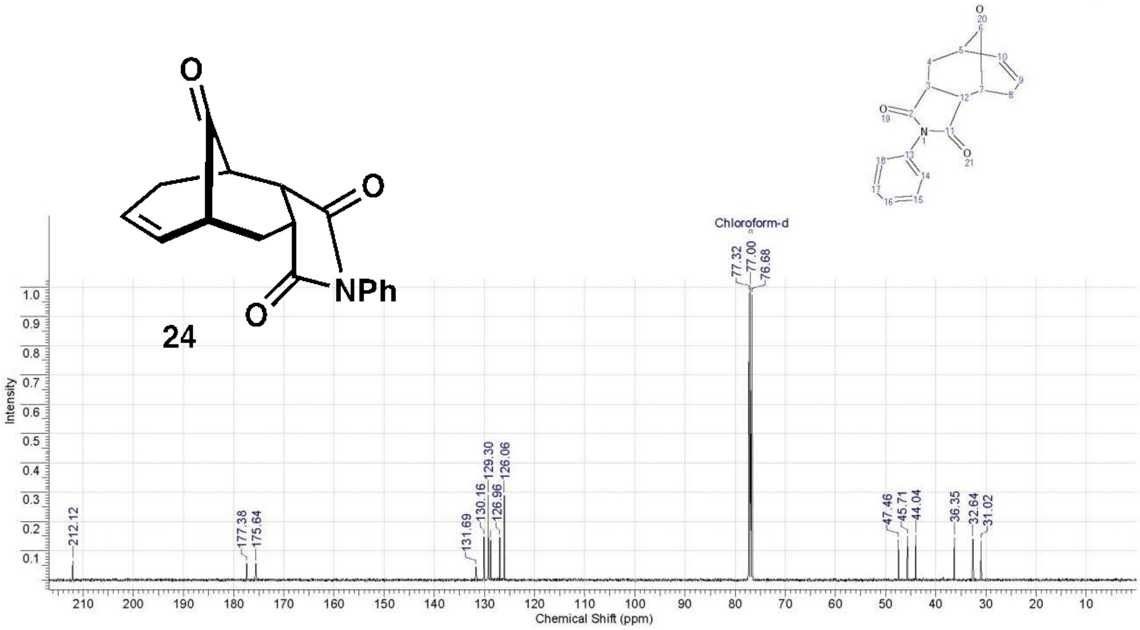

^{1}H NMR (400 MHz, $CDCl_3$)

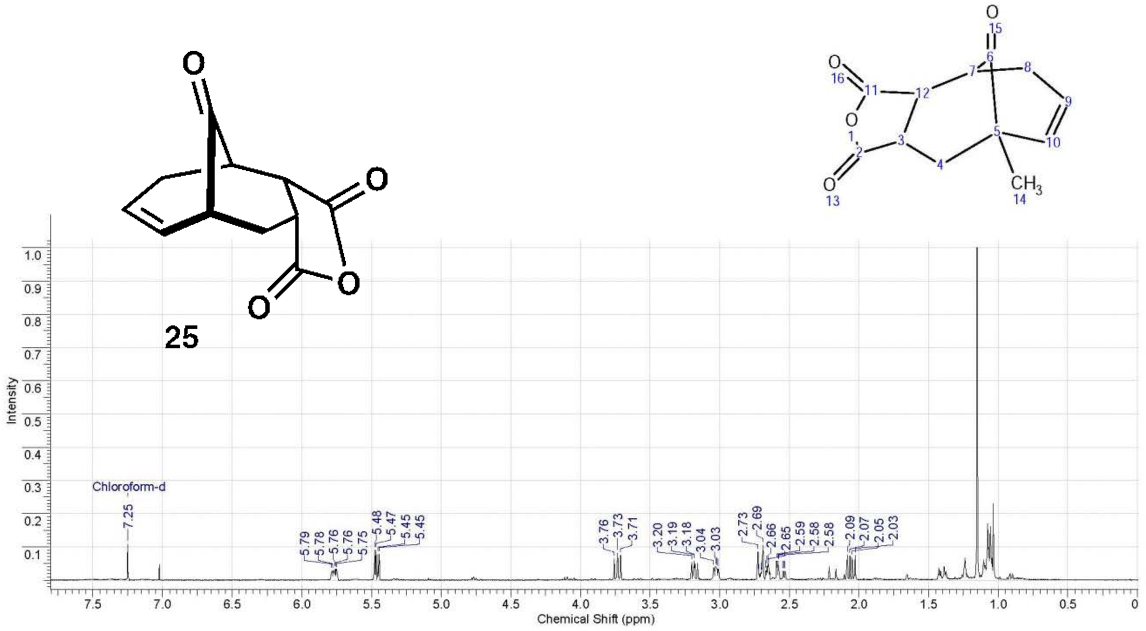

^{13}C NMR (101 MHz, $CDCl_3$)

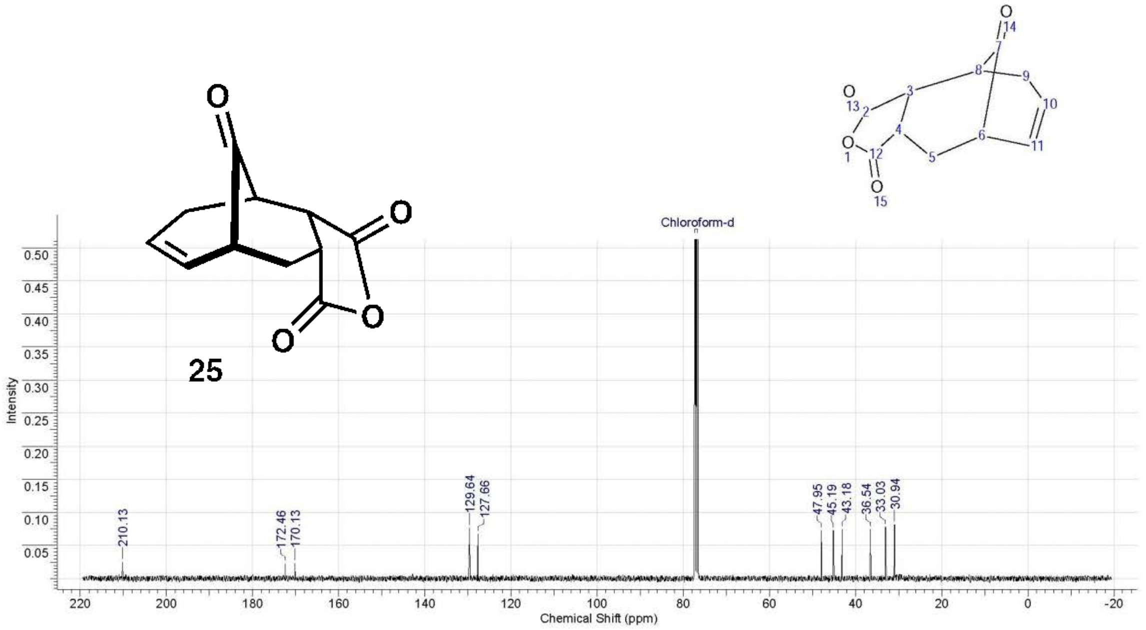

^{1}H NMR (400 MHz, $CDCl_3$)

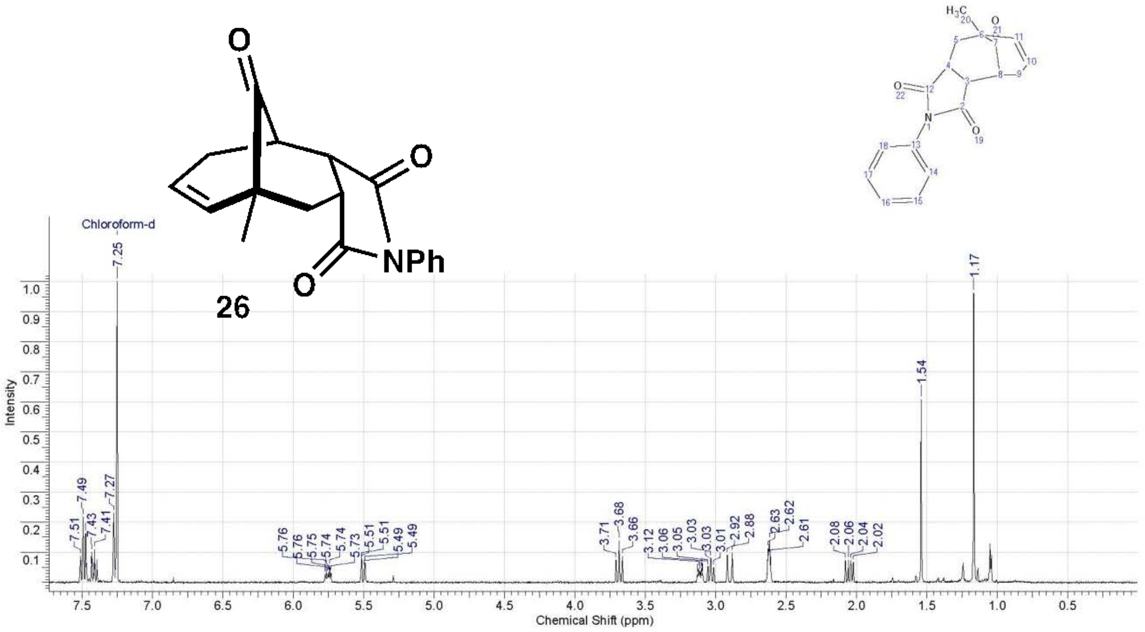

^{13}C NMR (101 MHz, $CDCl_3$)

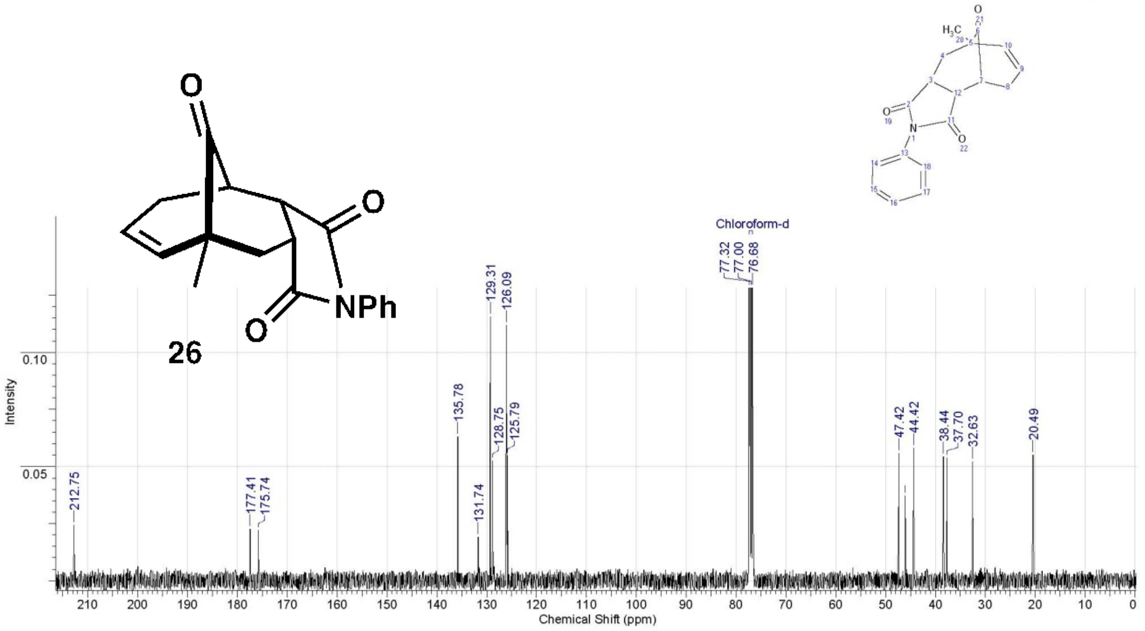

^{1}H NMR (400 MHz, $CDCl_3$)

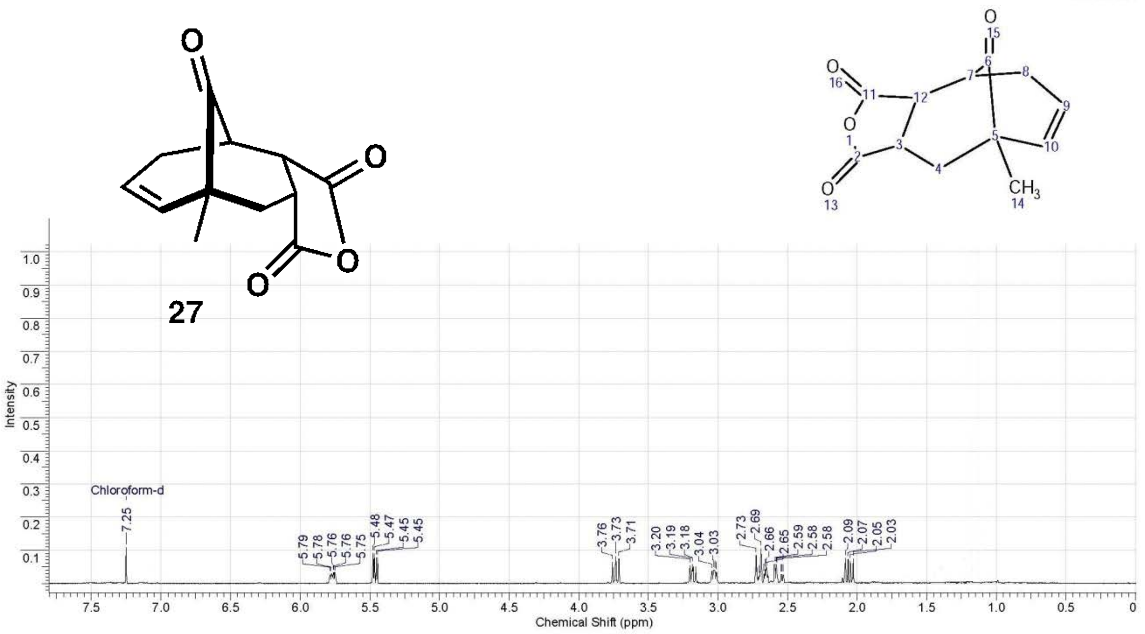

^{13}C NMR (101 MHz, $CDCl_3$)

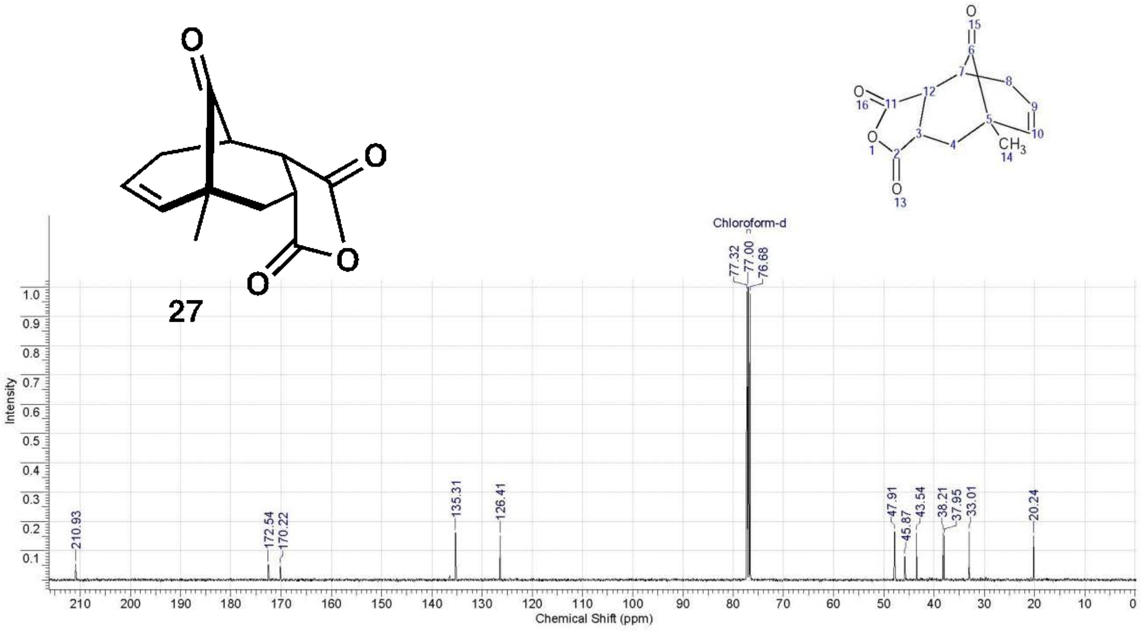

^{1}H NMR (400 MHz, $CDCl_3$)

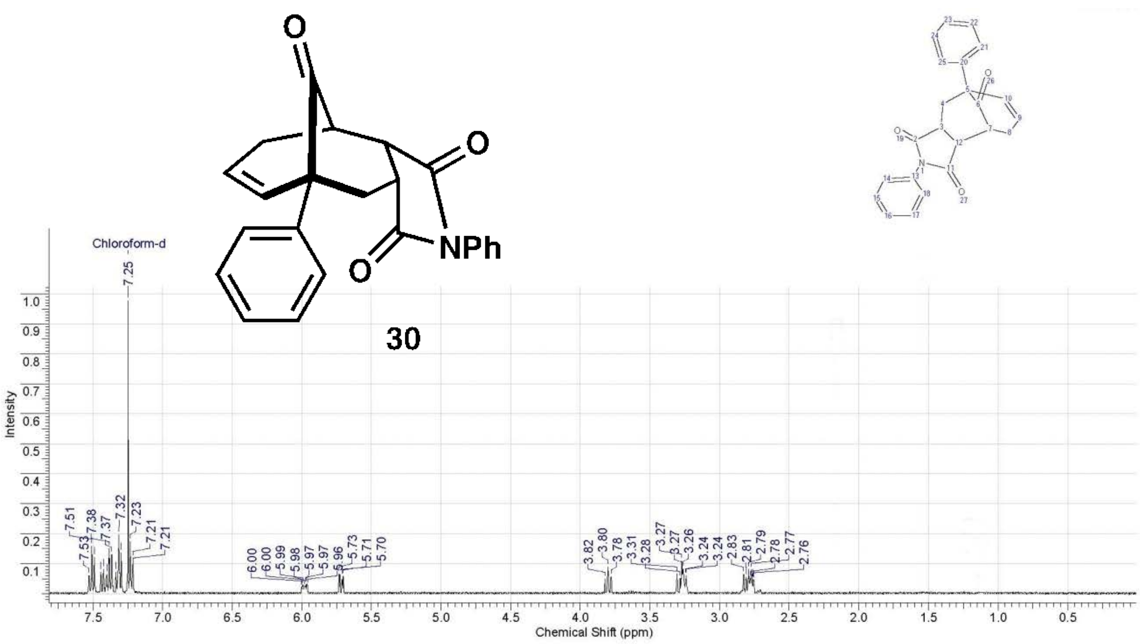

^{13}C NMR (101 MHz, $CDCl_3$)

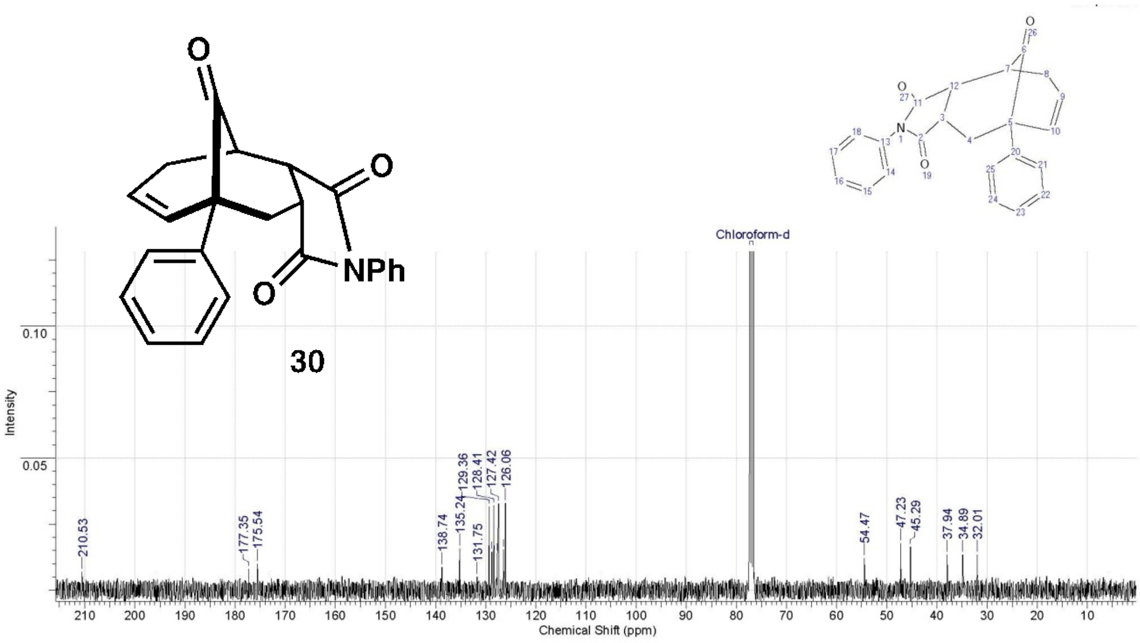

^{1}H NMR (400 MHz, $CDCl_3$)

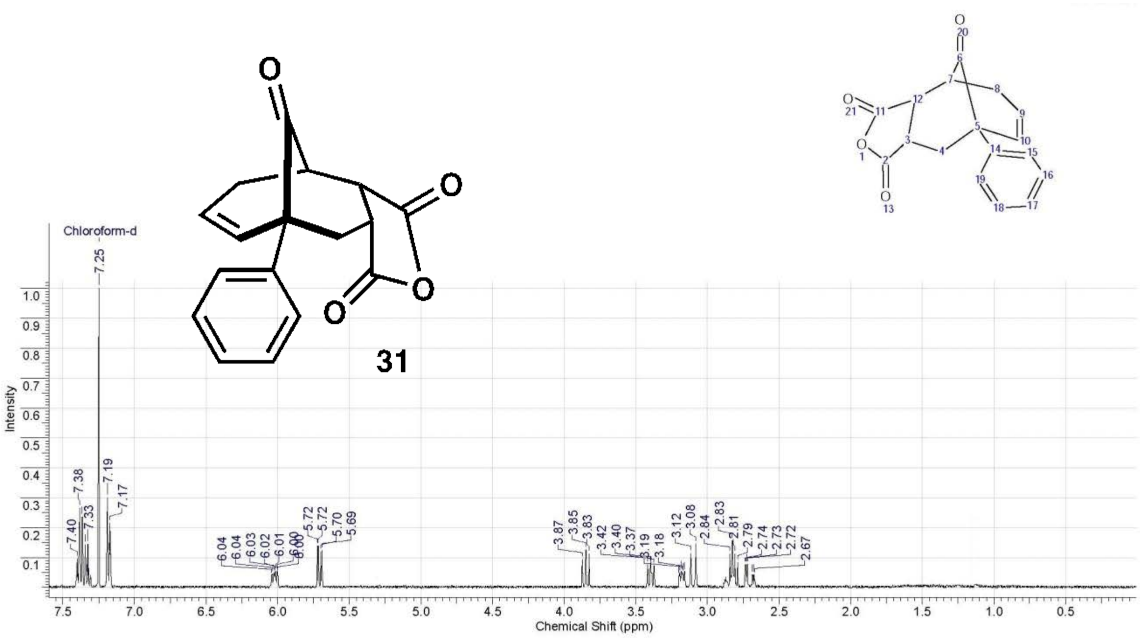

^{13}C NMR (101 MHz, $CDCl_3$)

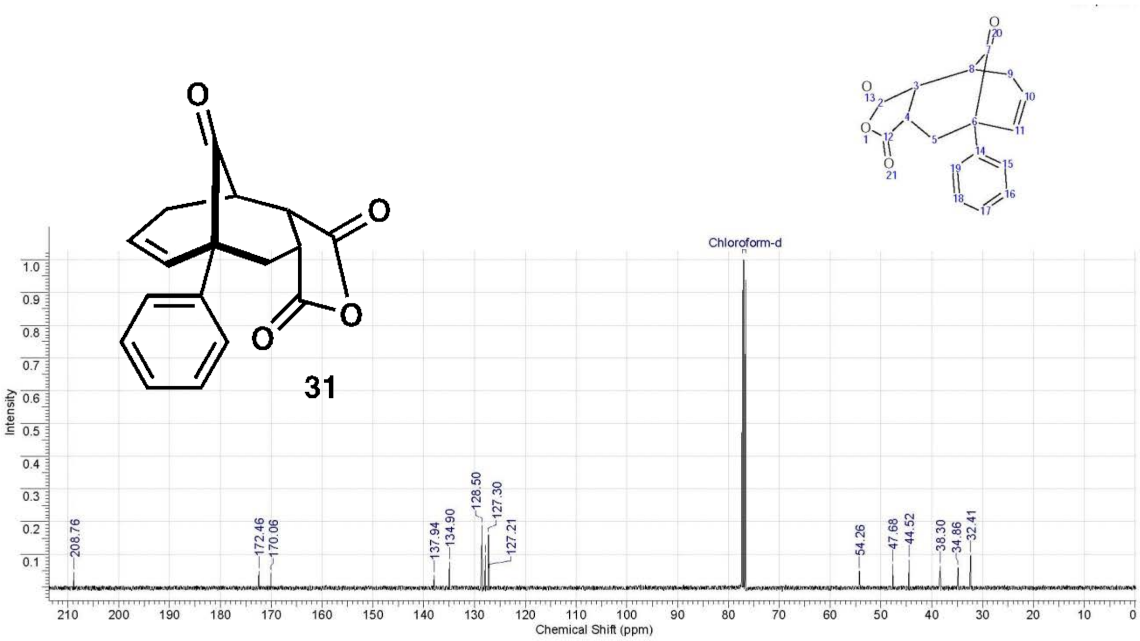

^{1}H NMR (400 MHz, $CDCl_3$)

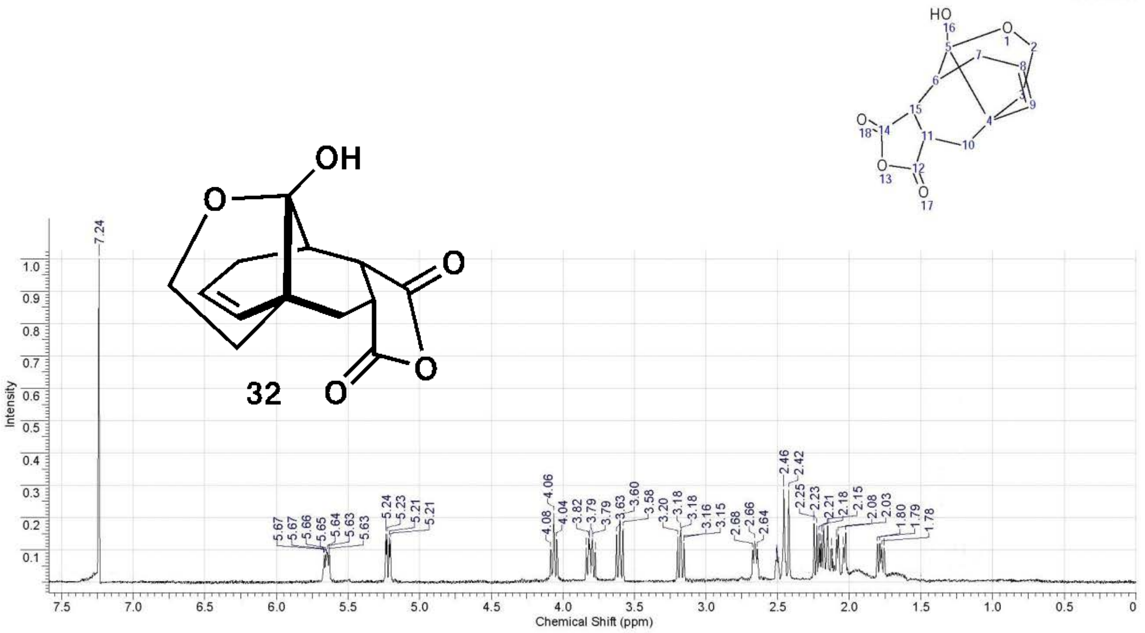

^{13}C NMR (101 MHz, $CDCl_3$)

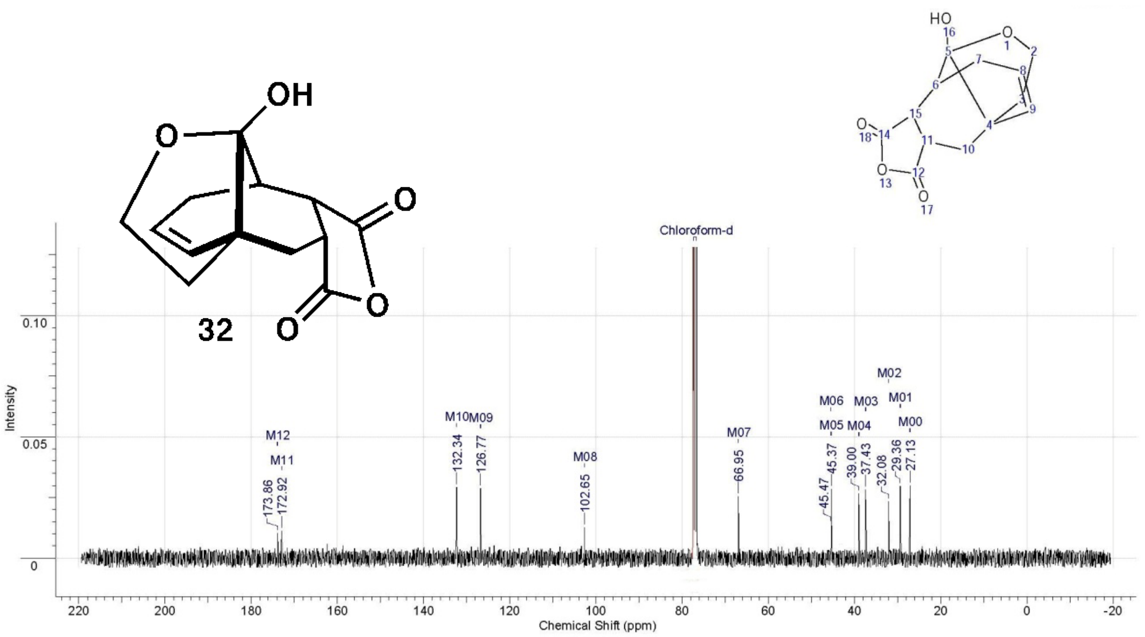

Compound **F** – ^{1}H NMR (400 MHz, $CDCl_3$)

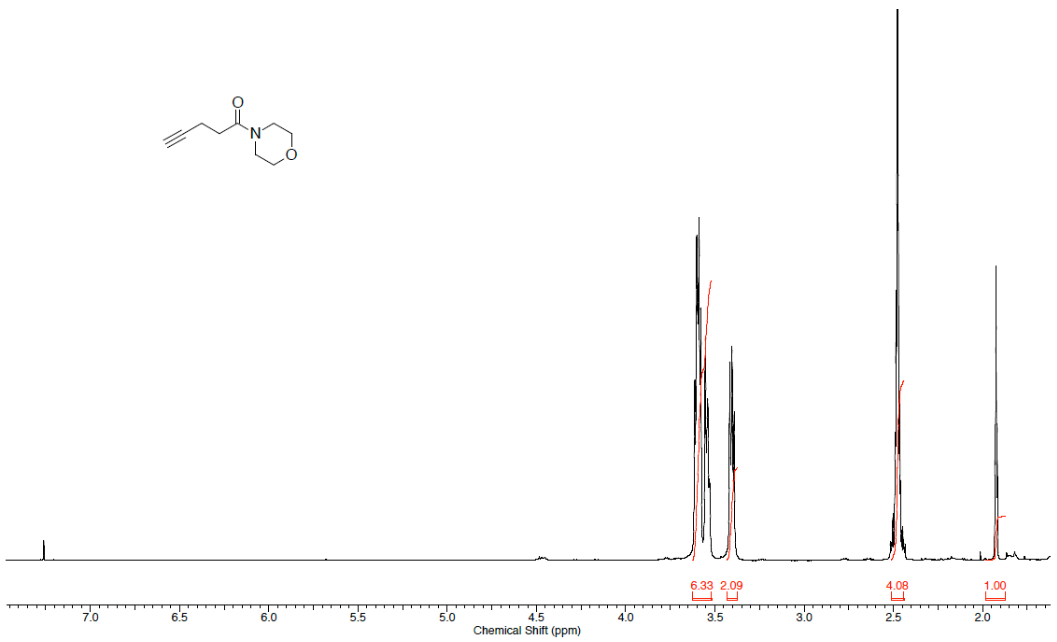

^{13}C NMR (101 MHz, $CDCl_3$)

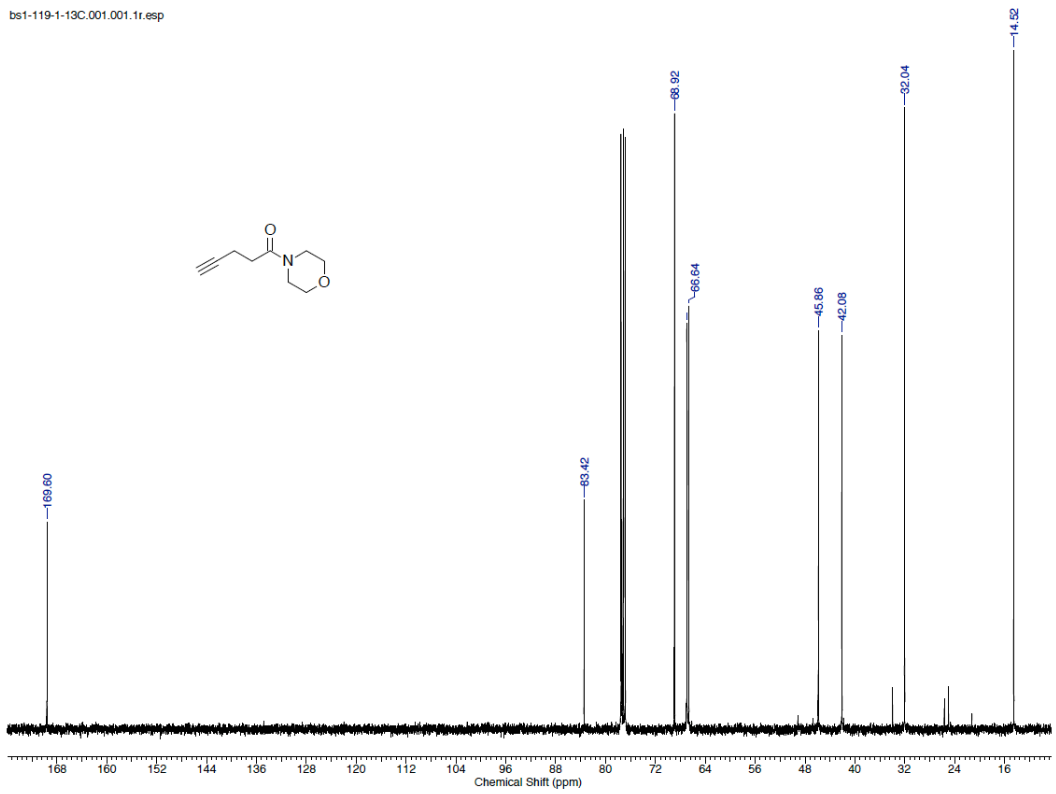

Compound **B** – ^{1}H NMR (400 MHz, $CDCl_3$)

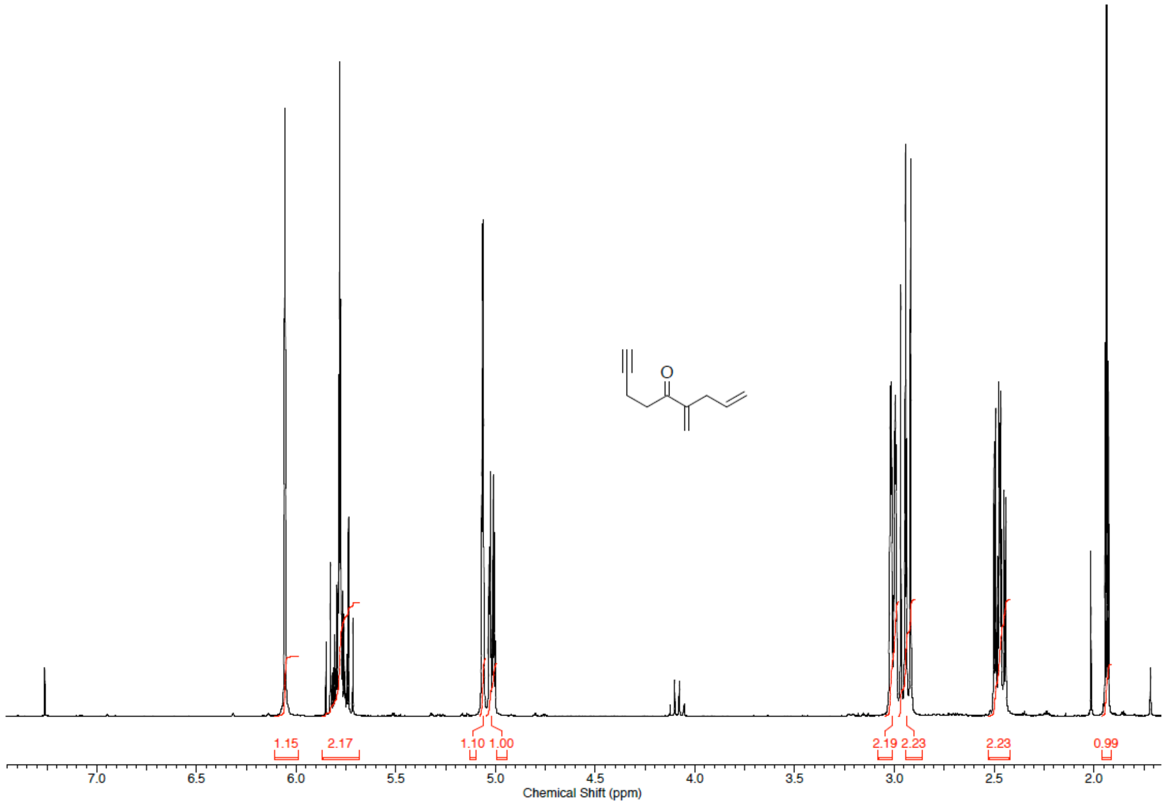

^{13}C NMR (101 MHz, $CDCl_3$)

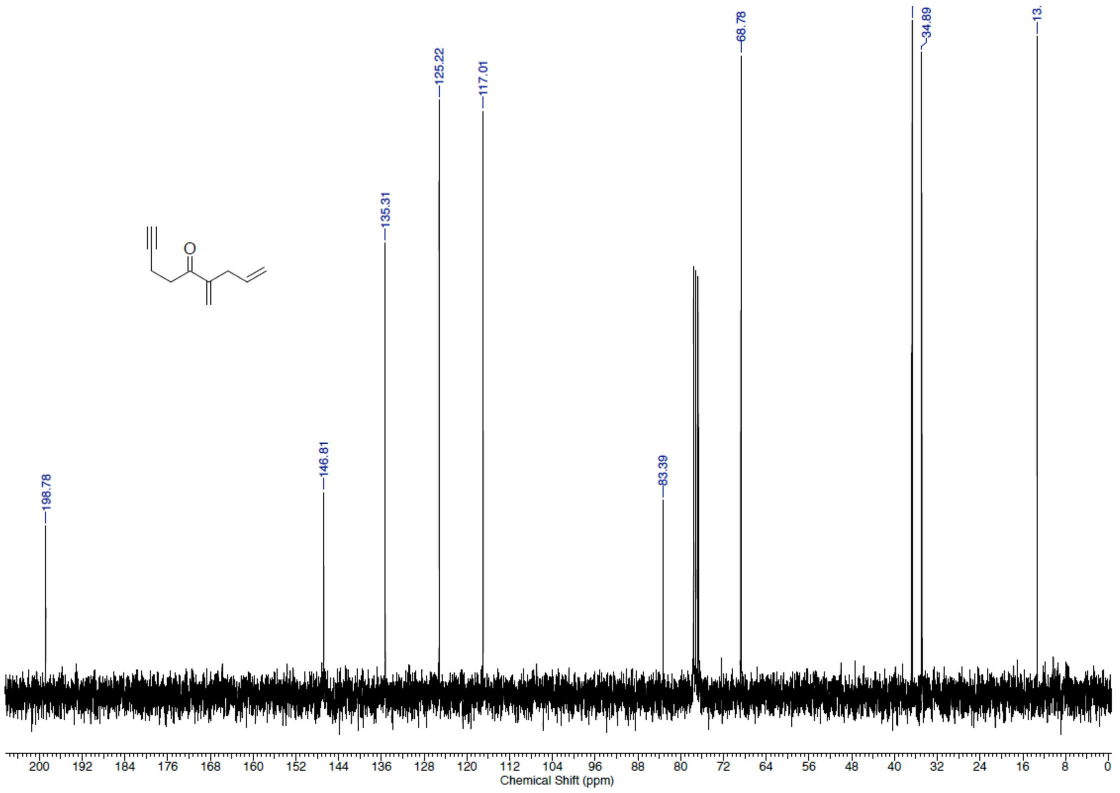

Compound **E** – ^{1}H NMR (400 MHz, $CDCl_3$)

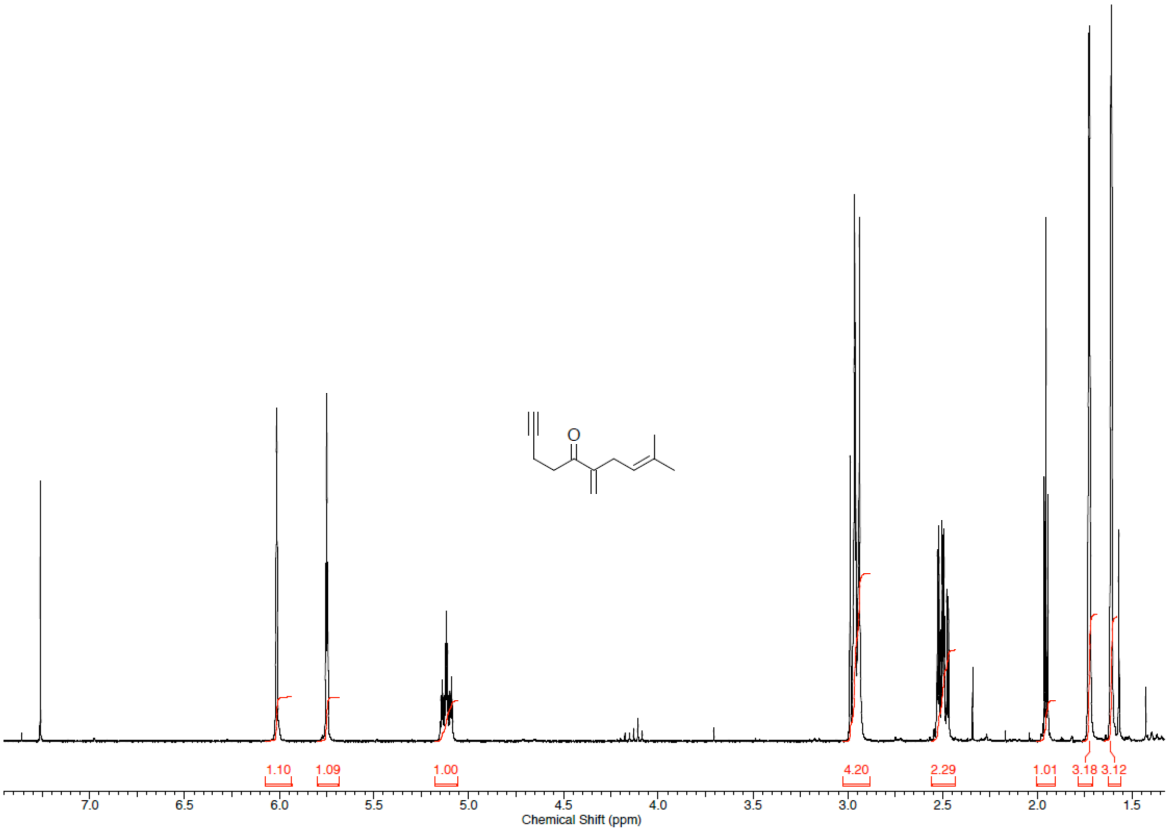

^{13}C NMR (101 MHz, $CDCl_3$)

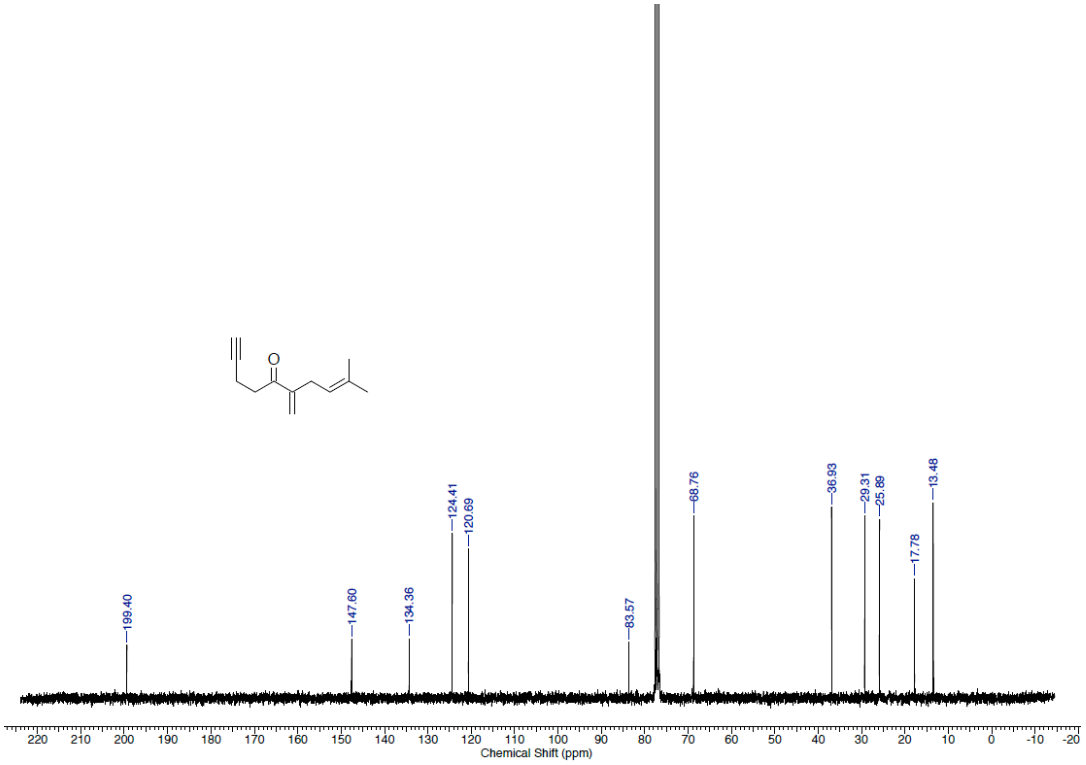

Compound **21** – ^{1}H NMR (400 MHz, $CDCl_3$)

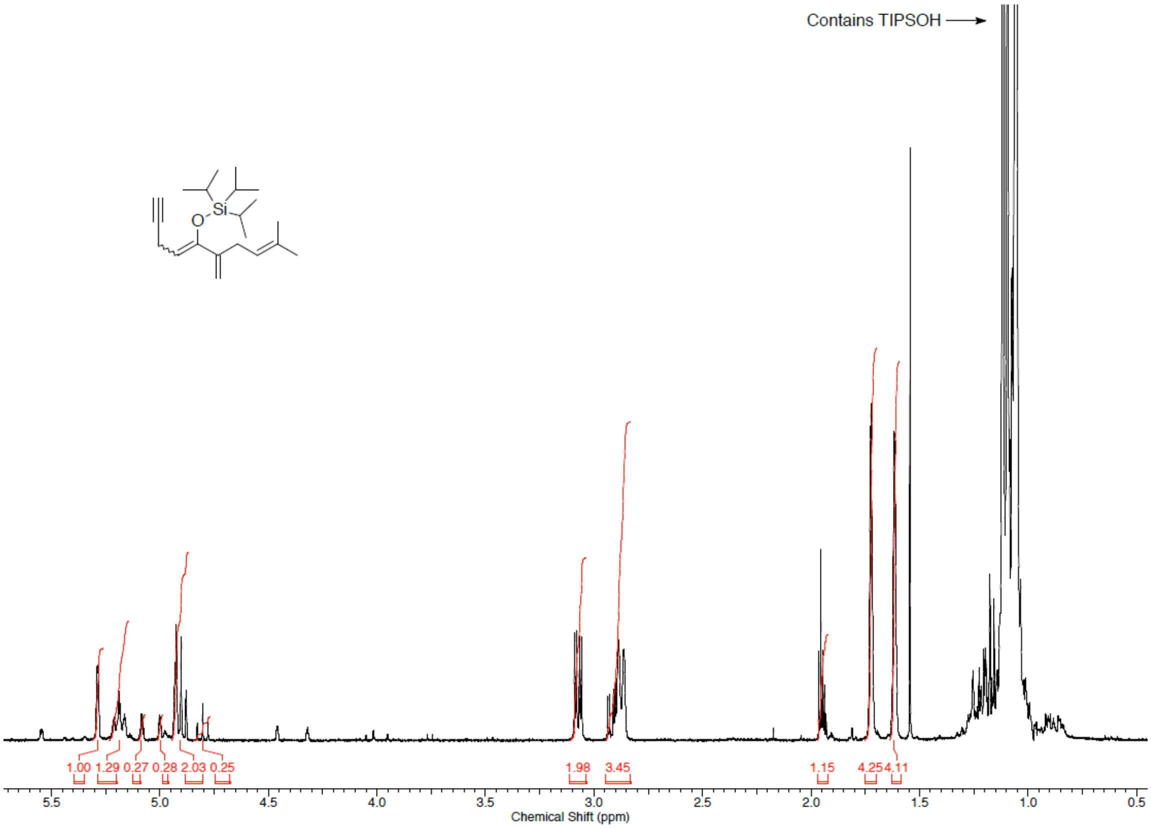

^{13}C NMR (101 MHz, $CDCl_3$)

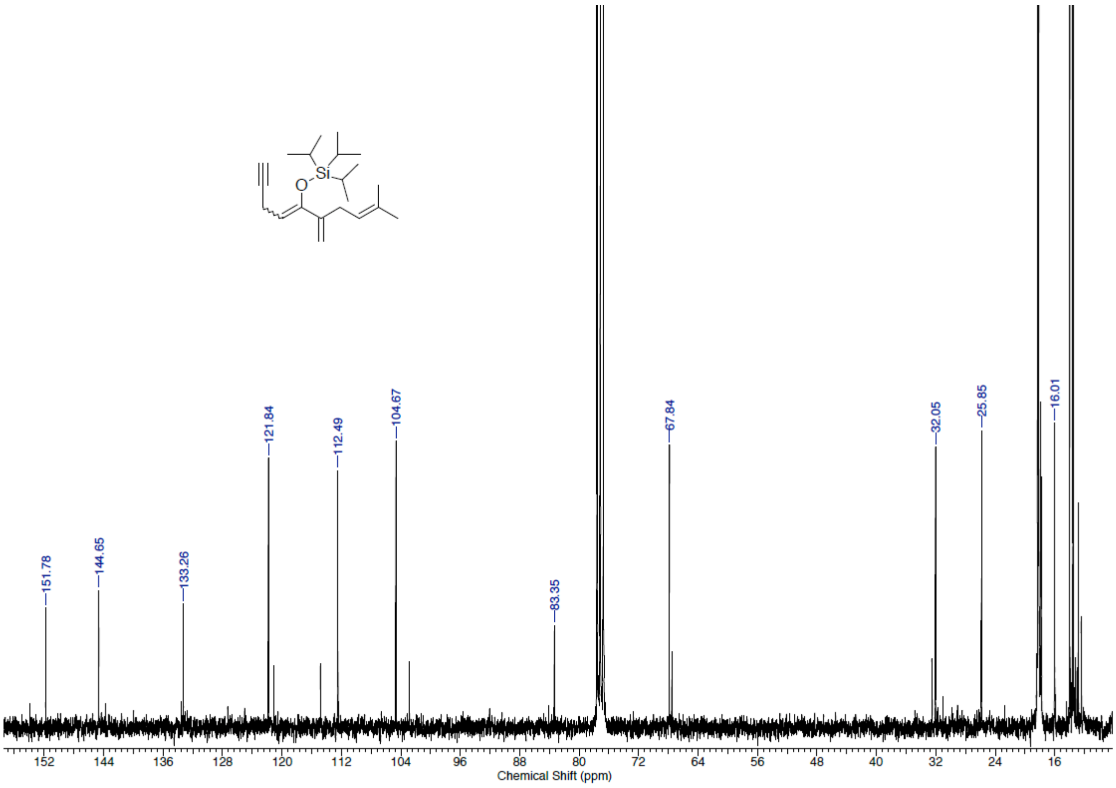

Compound **16** – ^{1}H NMR (400 MHz, $CDCl_3$)

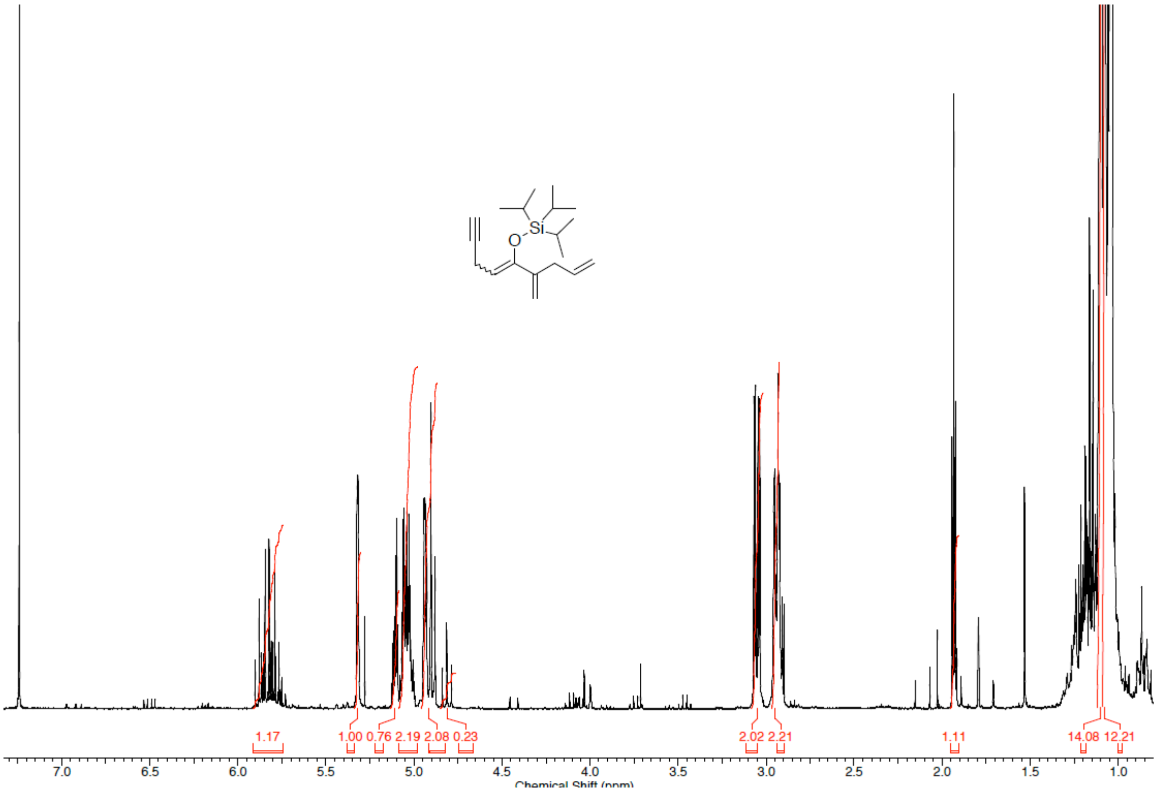

^{13}C NMR (101 MHz, $CDCl_3$)

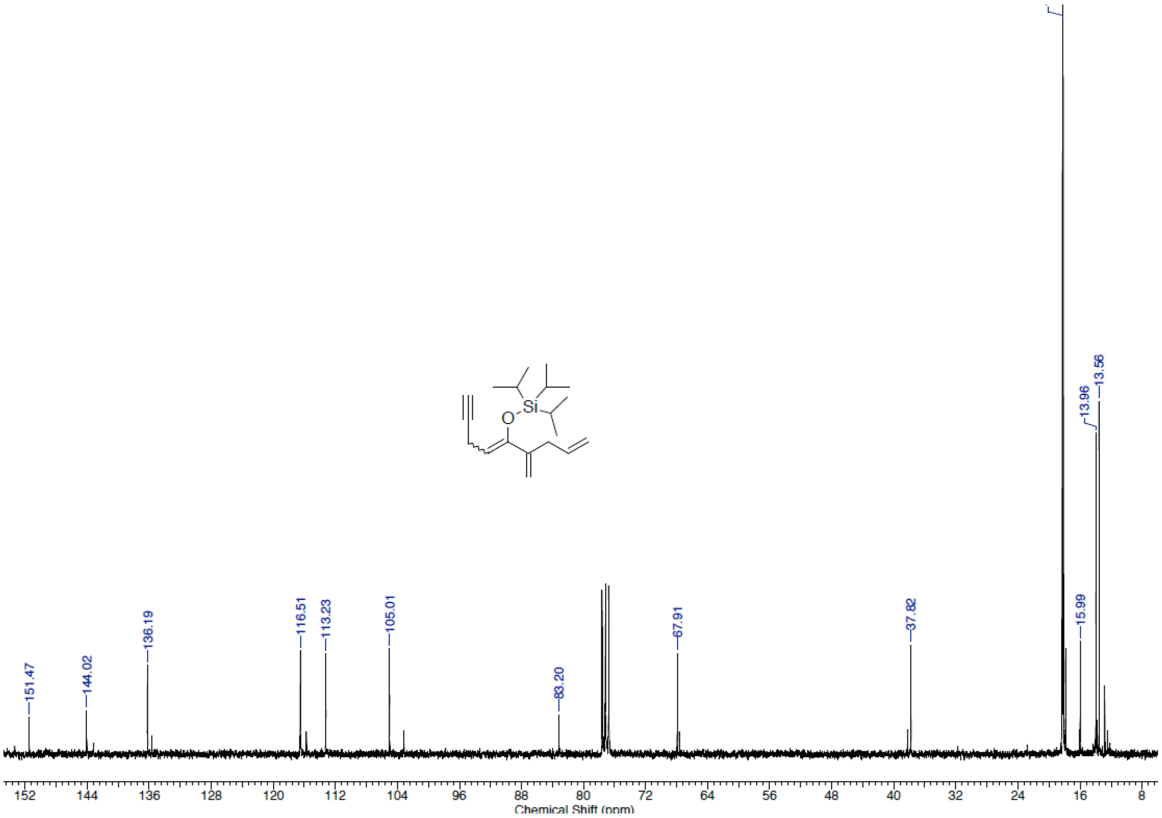

Compound **28** – ^{1}H NMR (400 MHz, $CDCl_3$)

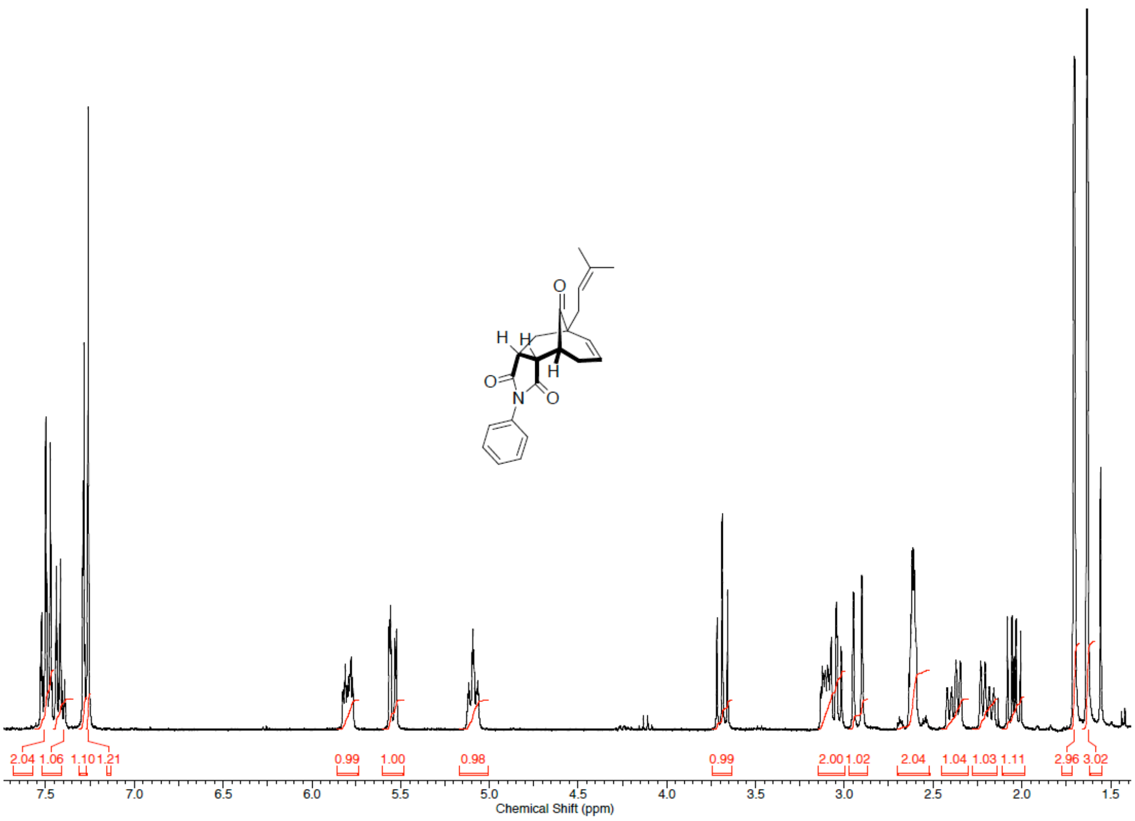

^{13}C NMR (101 MHz, $CDCl_3$)

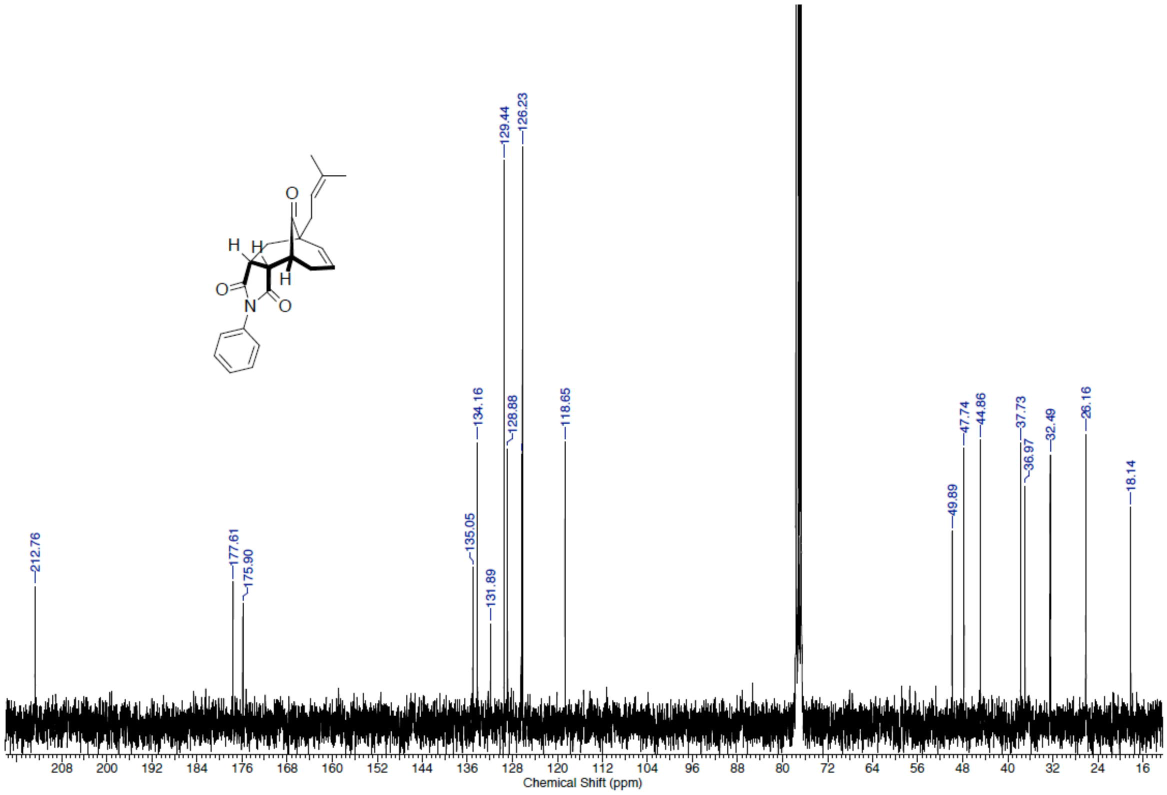

Compound **29** – ^{1}H NMR (400 MHz, $CDCl_3$)

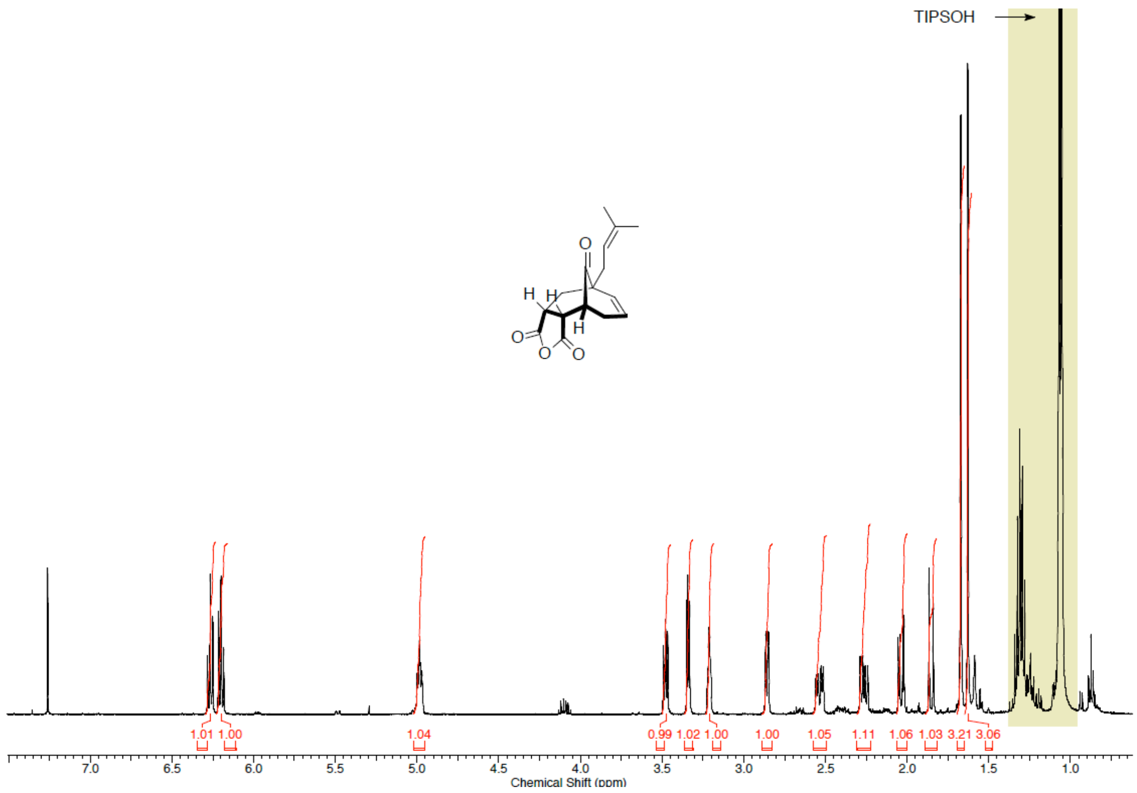

^{13}C NMR (101 MHz, $CDCl_3$)

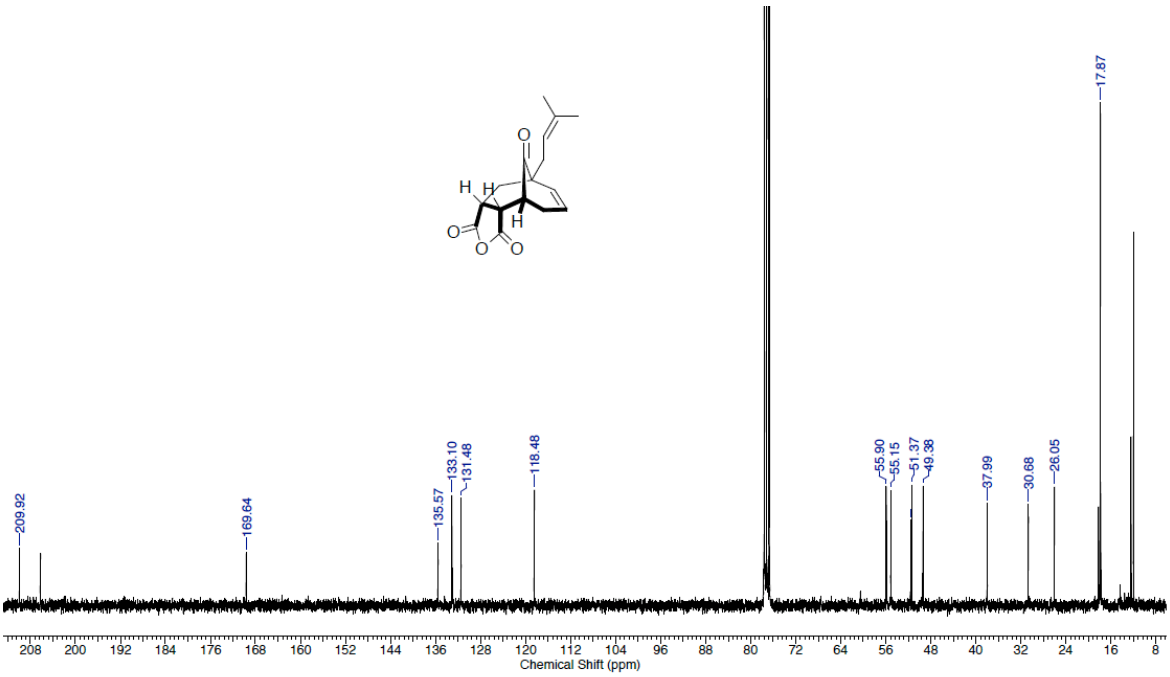

Compound **18** – ^{1}H NMR (400 MHz, $CDCl_3$)

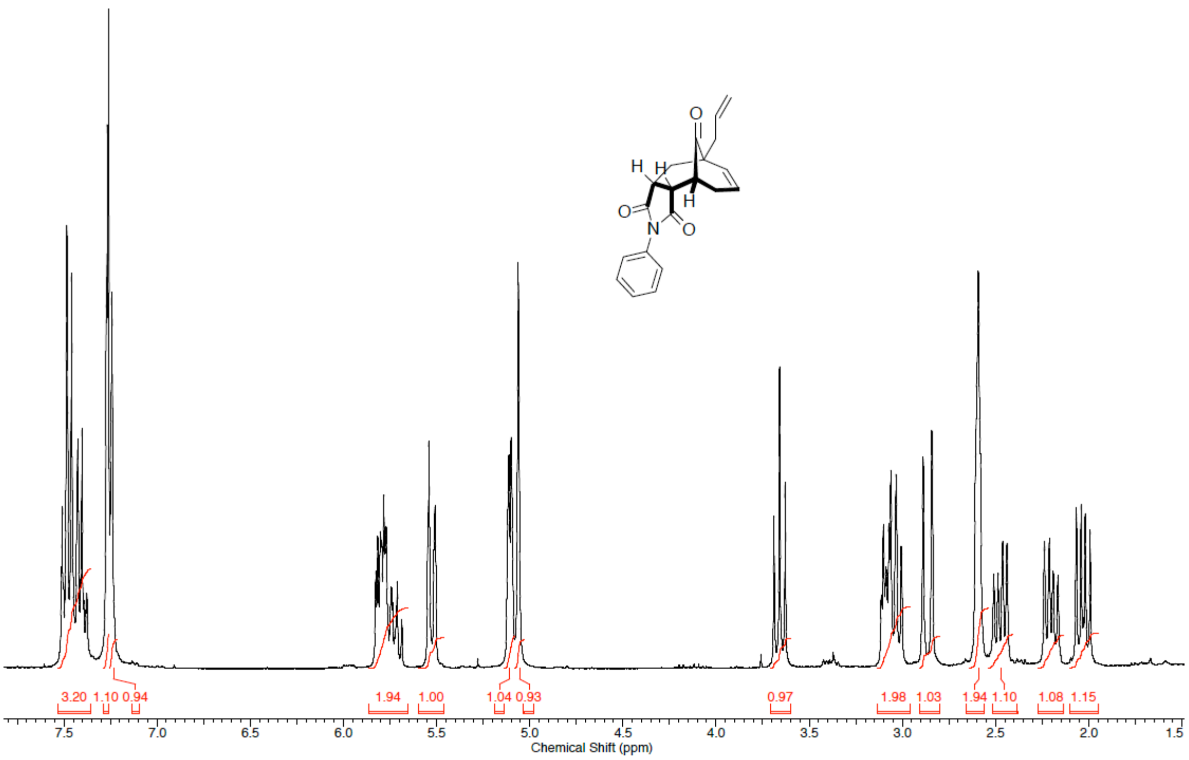

^{13}C NMR (101 MHz, $CDCl_3$)

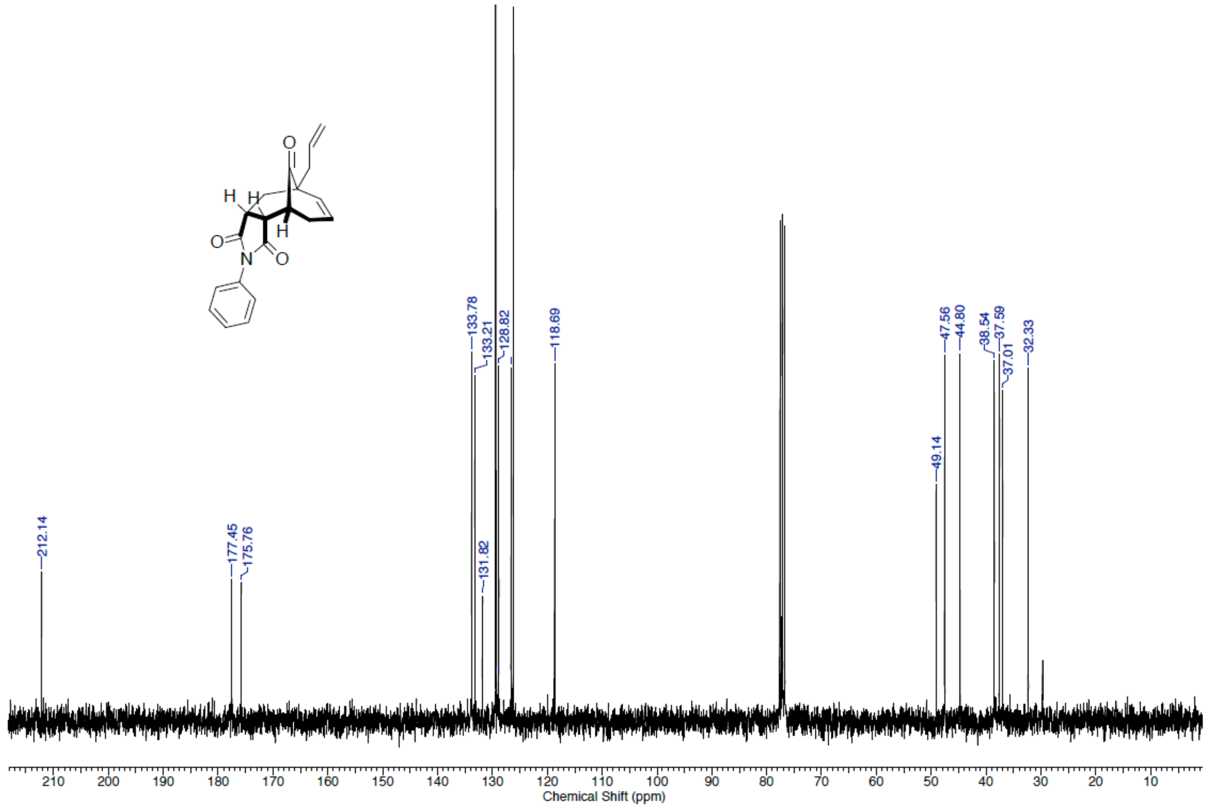

Compound **37** – ^{1}H NMR (400 MHz, $CDCl_3$)

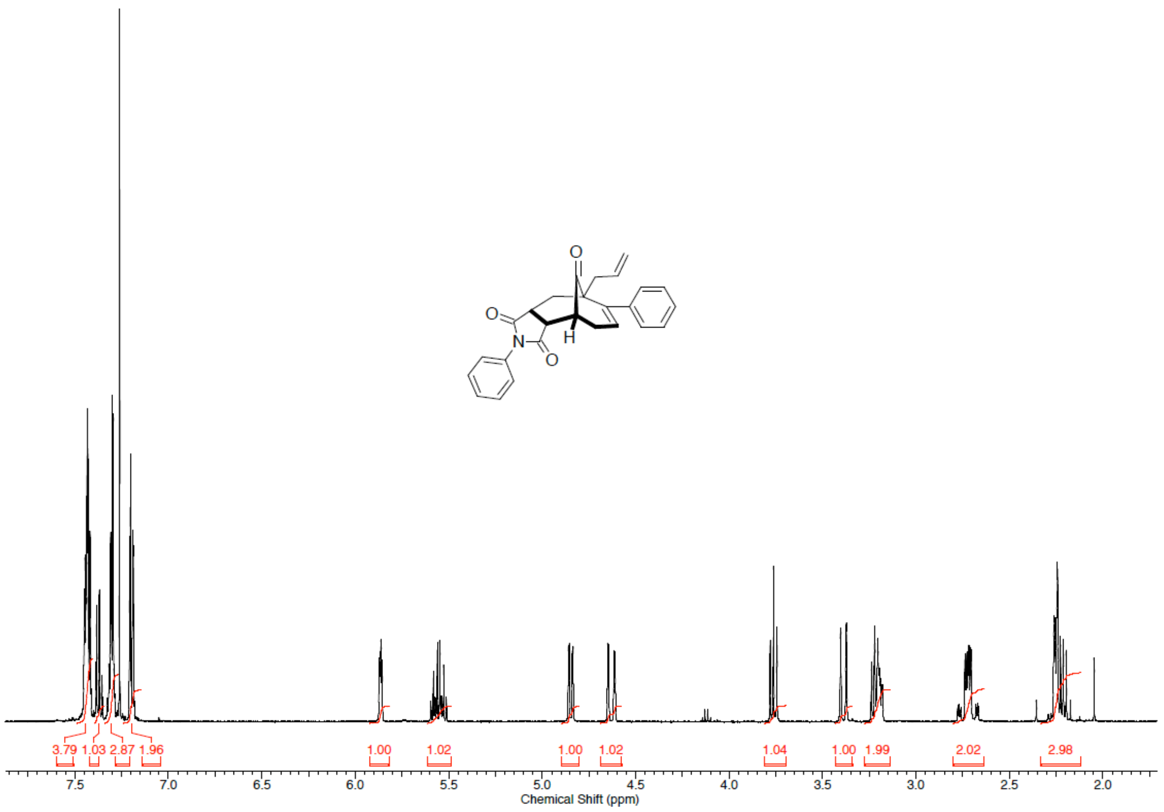

^{13}C NMR (101 MHz, $CDCl_3$)

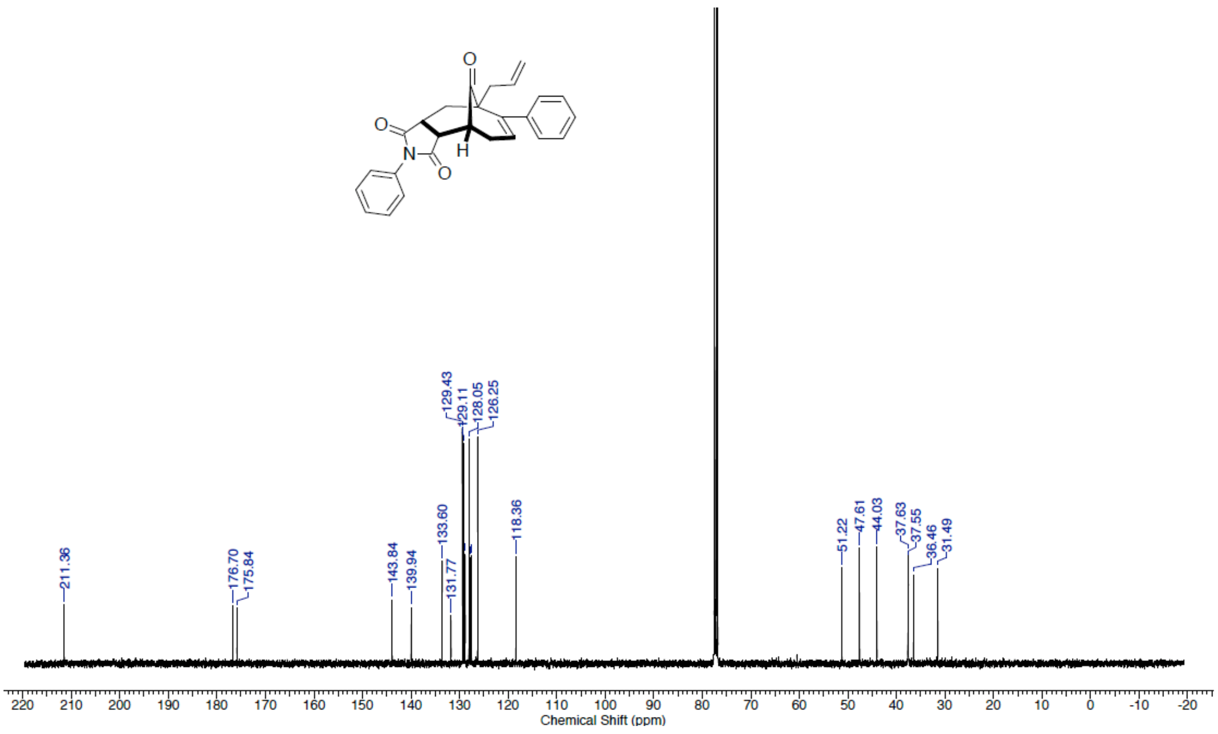

Compound **38** – ^{1}H NMR (400 MHz, $CDCl_3$)

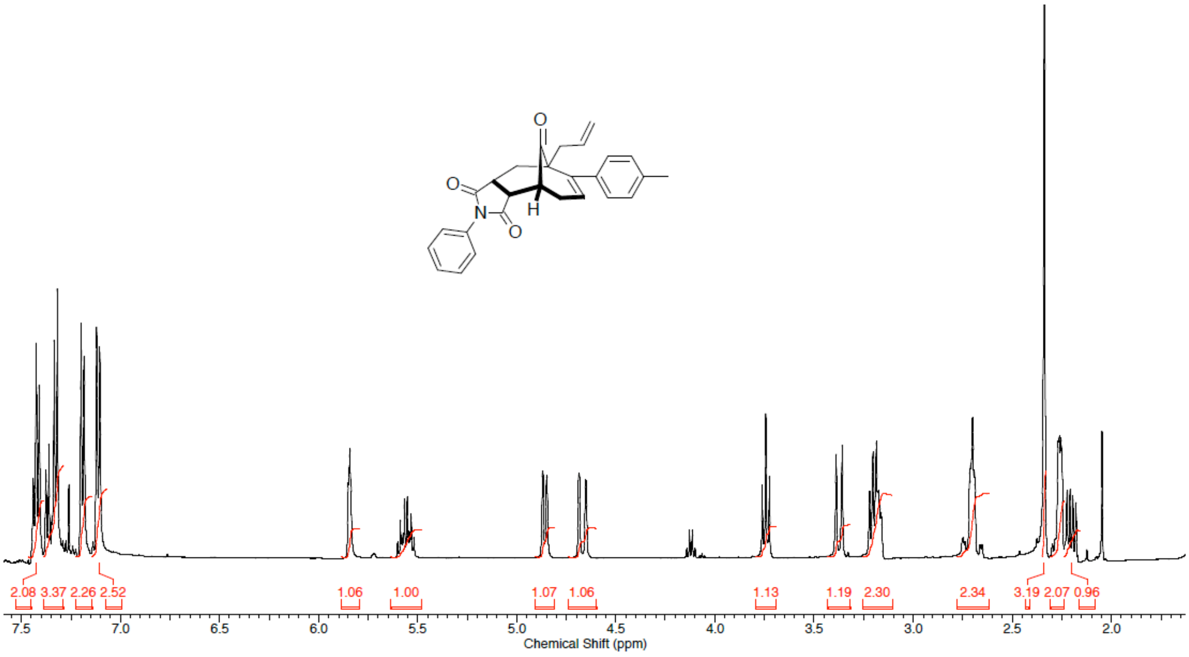

^{1}H NMR (400 MHz, $CDCl_3$)

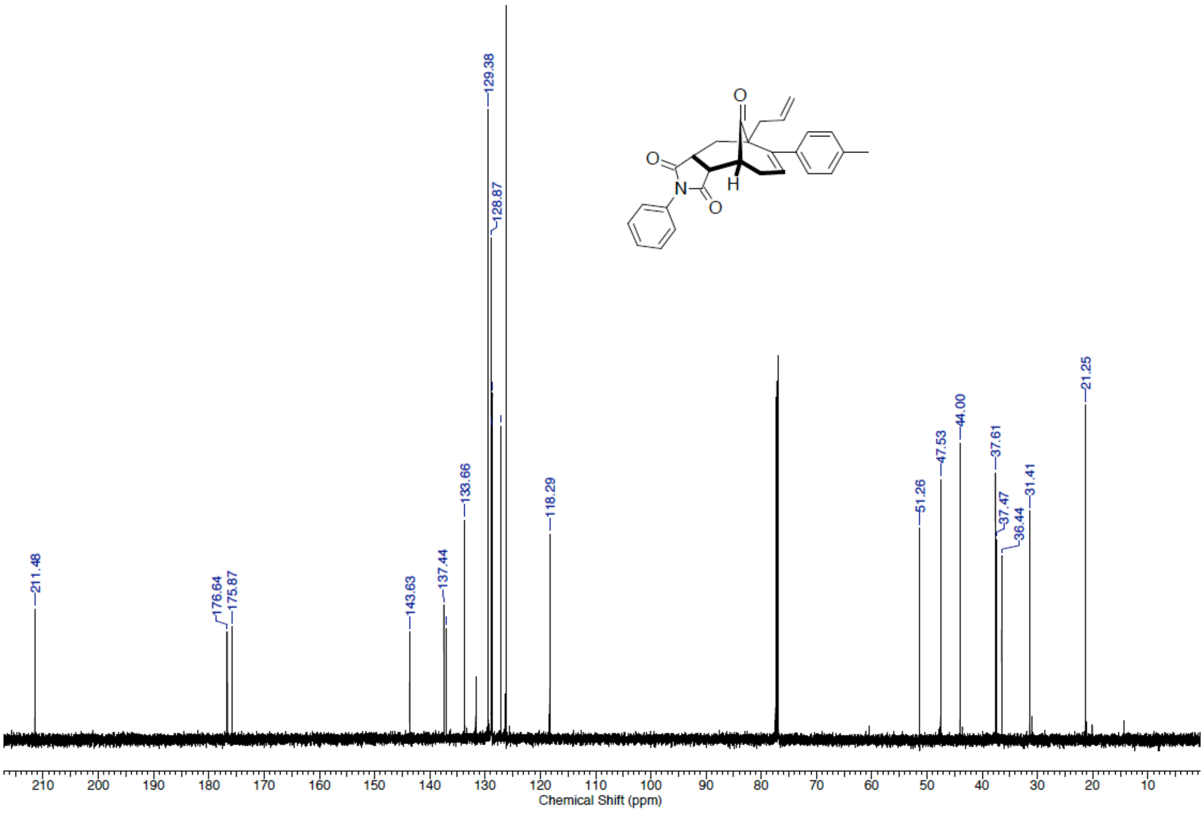

Compound **39** – ^{1}H NMR (400 MHz, $CDCl_3$)

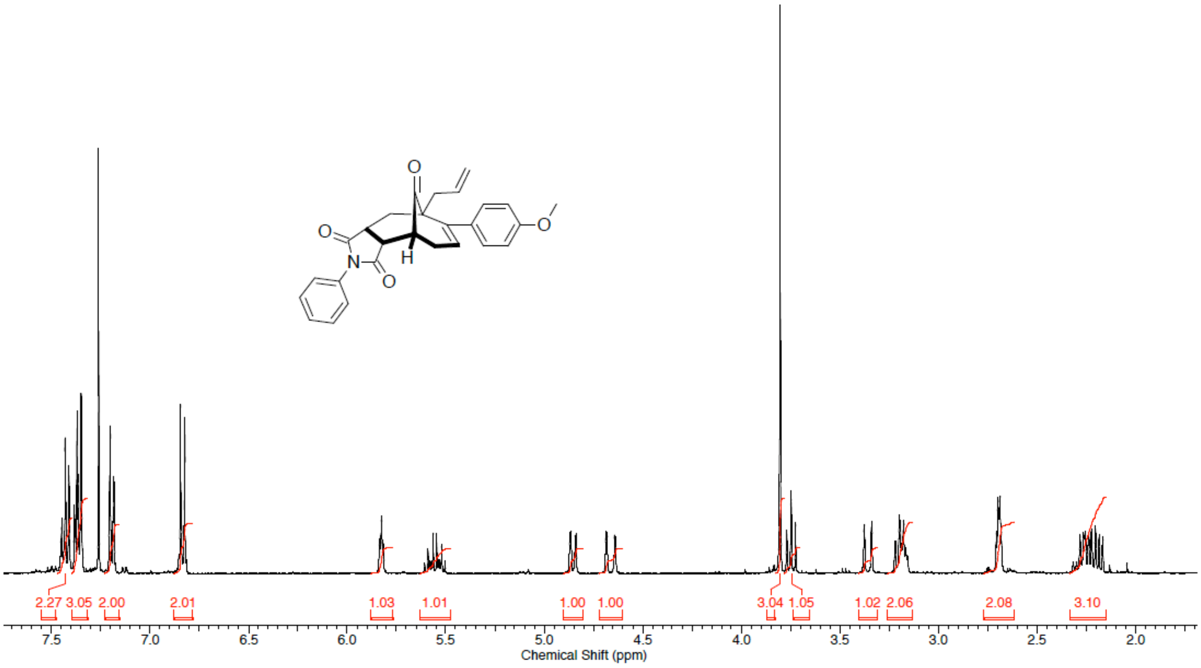

^{13}C NMR (101 MHz, $CDCl_3$)

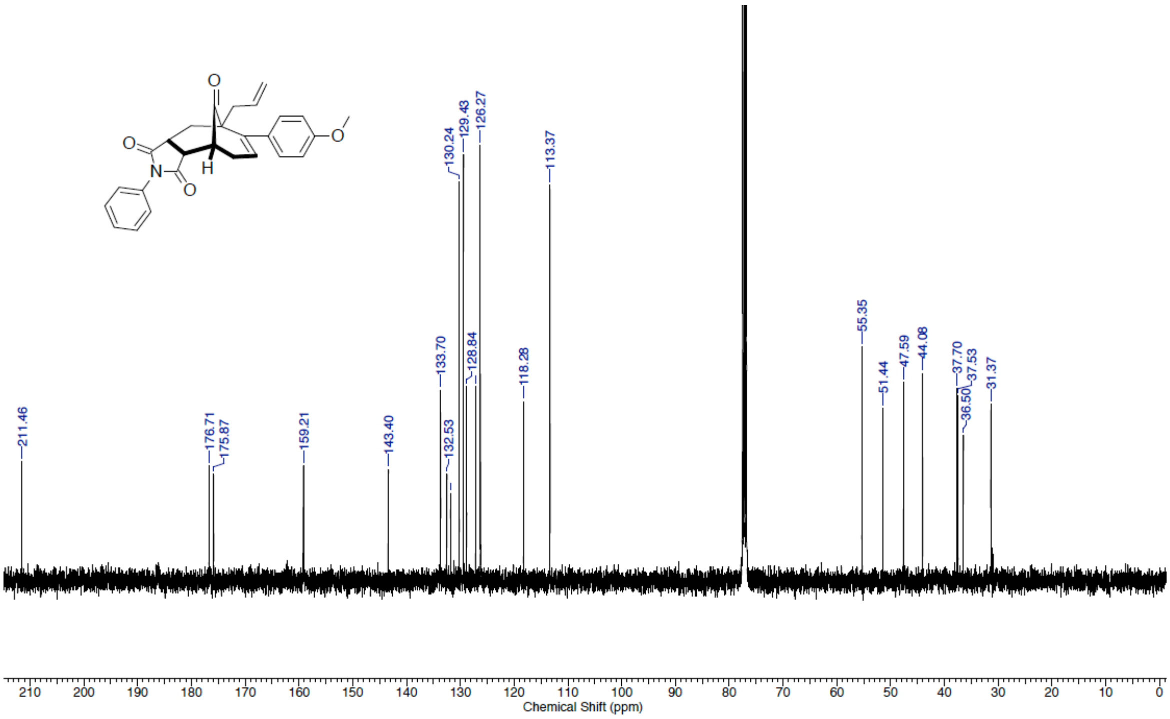

^{1}H NMR (400 MHz, $CDCl_3$) – Compound **40**

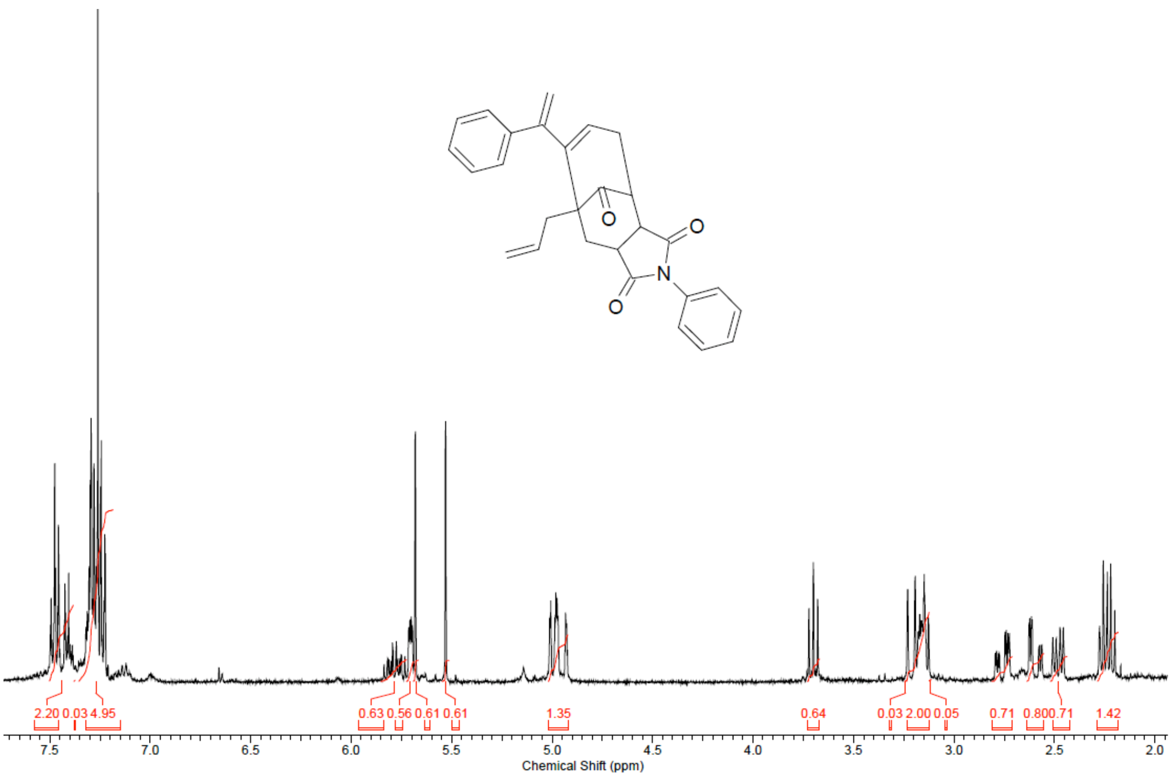

^{13}C NMR (101 MHz, $CDCl_3$)

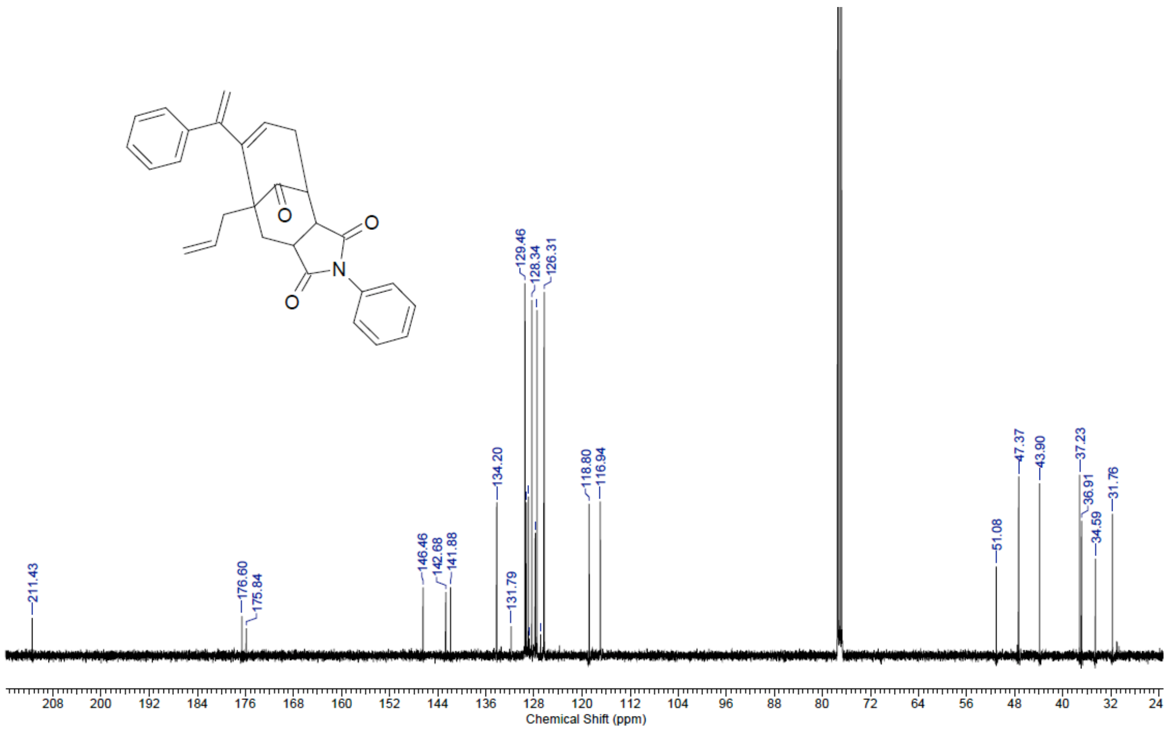

Supplement: File 1 — Experimental procedures, characterization data, 1H NMR and 13C NMR spectra. [file Beilstein_J_Org_Chem-07-1007-s001.pdf]
